# Alternative Presentations of Overall and Statistical Uncertainty for Adults’ Understanding of the Results of a Randomized Trial of a Public Health Intervention: Parallel Web-Based Randomized Trials

**DOI:** 10.2196/62828

**Published:** 2025-03-18

**Authors:** Christine Holst, Steven Woloshin, Andrew D Oxman, Christopher Rose, Sarah Rosenbaum, Heather Menzies Munthe-Kaas

**Affiliations:** 1 Centre for Epidemic Interventions Research Norwegian Institute of Public Health Oslo Norway; 2 Lisa Schwartz Foundation for Truth in Medicine Norwich, VT United States; 3 Dartmouth Institute for Health Policy and Clinical Practice Geisel Medical School West Lebanon, NH United States; 4 Cluster for Reviews and Health Technology Assessments, Division for Health Services Norwegian Institute of Public Health Oslo Norway

**Keywords:** communication, Grading of Recommendations Assessment, Development, and Evaluation language, GRADE language, statistical uncertainty, overall uncertainty, randomized trial

## Abstract

**Background:**

Well-designed public health messages can help people make informed choices, while poorly designed messages or persuasive messages can confuse, lead to poorly informed decisions, and diminish trust in health authorities and research. Communicating uncertainties to the public about the results of health research is challenging, necessitating research on effective ways to disseminate this important aspect of randomized trials.

**Objective:**

This study aimed to evaluate people’s understanding of overall and statistical uncertainty when presented with alternative ways of expressing randomized trial results.

**Methods:**

Two parallel, web-based, individually randomized trials (3×2 factorial designs) were conducted in the United States and Norway. Participants were randomized to 1 of 6 versions of a text (summary) communicating results from a study examining the effects of wearing glasses to prevent COVID-19 infection. The summaries varied in how *overall uncertainty* (“Grading of Recommendations Assessment, Development and Evaluation [GRADE] language,” “plain language,” or “no explicit language”) and *statistical uncertainty* (whether a margin of error was shown or not) were presented. Participants completed a web-based questionnaire exploring 4 coprimary outcomes: 3 to measure understanding of overall uncertainty (benefits, harms, and sufficiency of evidence), and one to measure statistical uncertainty. Participants were adults who do not wear glasses recruited from web-based research panels in the United States and Norway. Results of the trials were analyzed separately and combined in a meta-analysis.

**Results:**

In the US and Norwegian trials, 730 and 497 individuals were randomized, respectively; data for 543 (74.4%) and 452 (90.9%) were analyzed. More participants had a correct understanding of uncertainty when presented with plain language (United States: 37/99, 37% and Norway: 40/76, 53%) than no explicit language (United States: 18/86, 21% and Norway: 34/80, 42%). Similar positive effect was seen for the GRADE language in the United States (26/79, 33%) but not in Norway (30/71, 42%). There were only small differences between groups for understanding the uncertainty of harms. Plain language improved correct understanding of evidence sufficiency (odds ratio 2.05, 95% CI 1.17-3.57), compared to no explicit language. The effect of GRADE language was inconclusive (odds ratio 1.34, 95% CI 0.79-2.28). The understanding of statistical uncertainty was improved when the participants were shown the margin of error compared to not being shown: Norway: 16/75, 21% to 24/71, 34% vs 1/71, 1% to 2/76, 3% and the United States: 21/101, 21% to 32/90, 36% vs 0/86, 0% to 3/79, 4%).

**Conclusions:**

Plain language, but not GRADE language, was better than no explicit language in helping people understand overall uncertainty of benefits and harms. Reporting margin of error improved understanding of statistical uncertainty around the effect of wearing glasses, but only for a minority of participants.

**Trial Registration:**

ClinicalTrials.gov NCT05642754; https://tinyurl.com/4mhjsm7s

## Introduction

### Background

Public health messaging matters—it shapes how people understand important health risks and what can be done to mitigate them. Ideally, such messages are based on findings from robust research, but even important messages based on solid research can fail to properly reach the target audience when poorly communicated, as was seen during the COVID-19 pandemic [1]. Decision scientists have articulated basic principles for effective health communication, such as using simple and familiar wording, using clear visual design, presenting structured comparisons of alternatives, and careful testing in the target audiences [2-5].

A recent randomized trial assessed the effect of these principles on communication effectiveness in the context of COVID-19 home test kit instructions using a real example [6]. The trial showed that individuals randomized to instructions that did not follow best decision science principles (ie, the actual instructions authorized by the Food and Drug Administration), compared to those that did (ie, carefully pretested intervention instructions), were more likely to fail to quarantine when quarantine was the right choice (33% vs 14% failed; 95% CI for the 19% difference being 6%-31%).

Evidence from randomized trials documents the importance of both the format and content of health messaging. Formatting examples include how the use of percentages (eg, 10%) versus frequency formats (eg, 10 in 100) can improve comprehension [7] and how absolute versus relative risk measures for communicating treatment effects are better understood [3,8] and help people make decisions more consistent with their values [9].

Content examples include the importance of presenting both benefits and harms when describing interventions [10] and highlighting study limitations, such as how simple nondirective explanations about surrogate outcomes and newly approved drugs enhance evidence-based decision-making about prescription drugs [11,12].

Furthermore, there is evidence supporting the importance of communication about the uncertainty of research findings in both research summaries aimed at professionals [13-16] and plain language summaries for the public [17]. This includes both *statistical uncertainty* (ie, imprecision) [16] and the *overall uncertainty*, due to the risk of bias, inconsistency, indirectness, and publication bias [13,15]. However, there are still open questions about the best formats and language for presenting both kinds of uncertainty, and how people understand and react to such information.

A recent pair of trials [18] found that including “quality cues” in communications tempered the publics’ tendency to assume that the quality of evidence presented without such cues is high (when it was not), and reporting that evidence quality was low decreased trust, perception of intervention efficacy, and the likelihood of adopting it.

The certainty of the evidence can affect the decisions that people make. If the purpose of a message is to inform people rather than to persuade them [19], it is necessary to include information about the degree and source of uncertainty related to the effect estimate. Not doing so can be misleading.

This study is the first of several planned studies to evaluate strategies to improve communication of research findings (Message Lab). The goal of Message Lab is to develop and promote best practices in message development (ie, attending to the foregoing communication principles and evidence); facilitate user testing; and conduct randomized trials assessing the effects of public health messages on the public’s understanding of the messages, beliefs, decisions, and behaviors. Therefore, this study is also designed as a proof-of-concept exercise to develop and test a method to efficiently and effectively evaluate communication strategies intended to summarize the results of randomized trials using web-based trial platforms.

### Objectives

This study aimed to evaluate the effect of alternative formats for communicating overall and statistical uncertainty on the public’s understanding of uncertainty and the sufficiency of evidence. The objectives of the study were (1) to compare the effects of 3 ways of communicating the overall uncertainty of the effect of wearing glasses on reducing the chance of acquiring COVID-19, and (2) to compare the effects of including the margin of error (MOE; also called CI) compared to not including it.

## Methods

### Design

**Figure 1:**
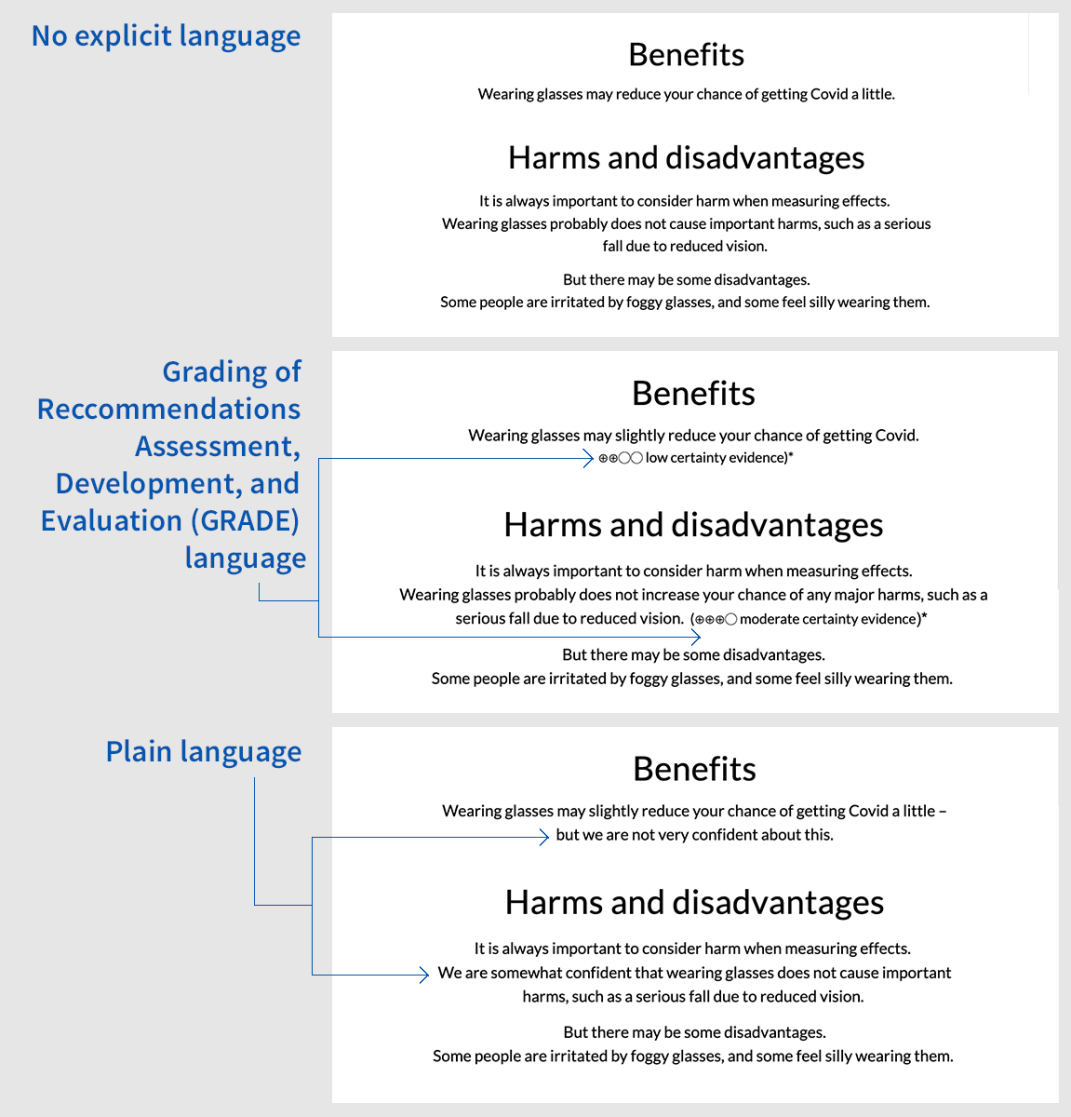
Three alternative versions presented to participants in the US trial communicating overall uncertainty about the possible benefits and harms of wearing glasses to reduce the chances of acquiring COVID-19.

**Figure 2:**
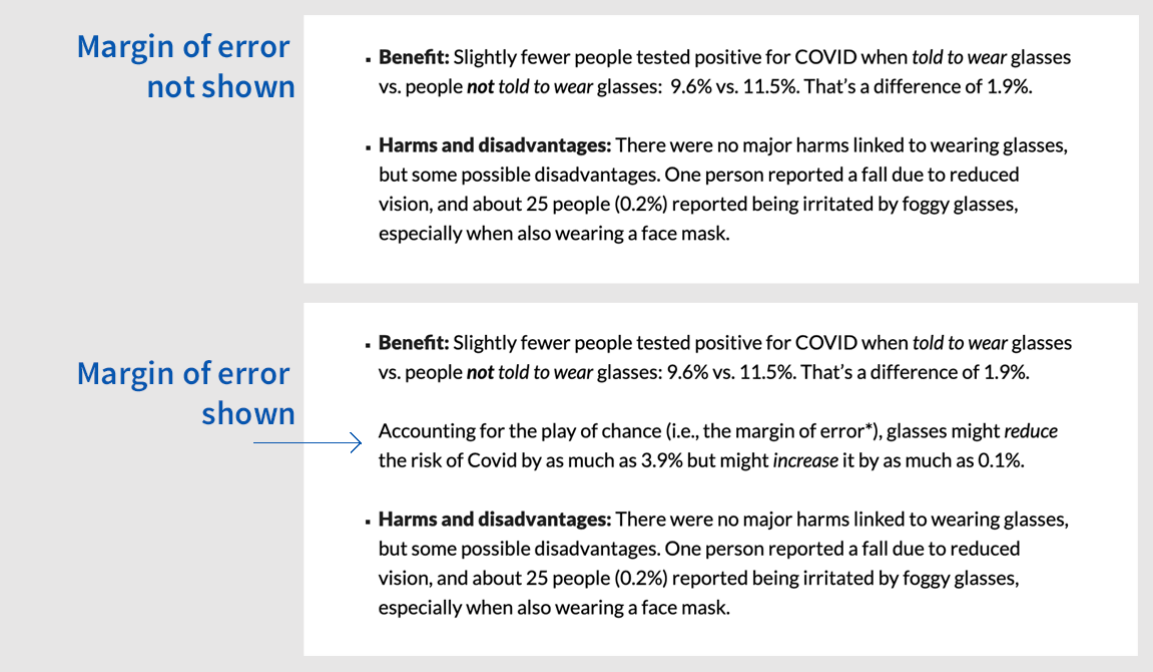
Two alternative versions presented to participants in the US trial communicating statistical uncertainty (margin of error).

We designed a web-based, parallel-group, individually randomized, pragmatic trial to compare the effects of different ways of communicating uncertainty when reporting the results of a randomized trial to the public. The trial was prespecified, registered at ClinicalTrials.gov (NCT05642754) Zenodo (7428981), and, except as noted, conducted following a published protocol [20]. We used a published trial assessing the effect of wearing glasses on the risk of being infected with COVID-19 as our example [21]. We used a 3×2 factorial trial design because we were interested in the effects of presenting overall uncertainty in each of 3 ways (ie, Grading of Recommendations Assessment, Development and Evaluation [GRADE] language, plain language, or no explicit language) combined with the effects of presenting or not presenting statistical uncertainty (in the form of an MOE). This resulted in 6 (3×2) groups differing in how the COVID-19 study was summarized (Figures 1 and 2).

### Recruitment of Participants

We implemented the design in 2 nearly identical trials in Norway and the United States in April 2023 and May 2023. For each trial, we used quota sampling from a web-based platform (Prolific; Prolific Academic Ltd) of volunteer research participants in the US trial [22] and from an independent commercial research agency (Opinion) [23] with a panel of 120,000 people living in Norway. All participants were thus part of established research panels, which provide recruitment and management of participants for online research. Participants were eligible if they were aged ≥18 years and said that they did not regularly wear glasses (based on a prescreen feature in Prolific and a screener question in Opinion). In Norway, 2667 persons received an invitation from Opinion to participate, while the study was open to 34,242 eligible persons in the Prolific platform (see the Results section for more details). Participants were literate in English (used in the US version) or Norwegian (used in the Norwegian version). Both platforms applied processes to prevent bots and fraudulent participants [24], and to further increase data quality, we built in attention and comprehension checks [25].

### Randomization

Eligible participants were randomized in a 1:1:1 ratio to 1 of the 6 comparison groups (described above). In the United States, participants were randomized via the web-based questionnaire after clicking on the link to the summary. In Norway, the participants were randomized to each summary by the research agency.

### Interventions and Control

#### Overview

The alternative versions of the presentation of overall and statistical uncertainty included in the 6 summaries are provided in Figures 1 and 2, and the full summaries are provided in Multimedia Appendix 1. Each version included one of the alternatives for communicating overall uncertainty about the benefits and harms (ie, GRADE, plain, or no explicit language) and either included or did not include a presentation of the MOE:

- Version 1: no explicit language and MOE not shown (reference or control version)
- Version 2: no explicit language and MOE shown
- Version 3: plain language and MOE not shown
- Version 4: plain language and MOE shown
- Version 5: GRADE language and MOE not shown
- Version 6: GRADE language and MOE shown

#### Development and User Testing of the Interventions

The summaries were drafted in English by 4 researchers (SW, AO, HMK, SR) and translated to Norwegian by 2 researchers (IHE, CH). Note that the summaries underwent multiple rounds of user testing and modifications as needed before the trial. We used human-centered design methods to develop the summaries to present to the participants [26]. Initially, we invited 4 native English speakers and 3 native Norwegian speakers to unmoderated user testing using Loop11, a digital user experience platform [27]. In total, 7 user test participants (n=5, 71% female and n=2, 29% male; n=2, 29% with secondary school education and n=5, 71% with postsecondary education; n=2, 29% living in Canada and n=5, 71% living in Norway; and n=3, 43% aged >55 years and n=4, 57% aged between 30 and 45 years) were introduced to the project and asked to imagine themselves in the following scenario: “Imagine you hear that glasses may reduce your chance of becoming infected with COVID. You go online to find out more information and find a website that says... [insert COVID trial summary version 1, 2 or 3].”

They were then asked to read 5 pieces of text: 3 versions of the summaries with progressively more information (eg, the first version was the shortest summary with no text related to uncertainty or the MOE, the second version had text related to uncertainty but not the MOE, and the third version had text related to uncertainty and the MOE); the trial questions; and the text that would be available via hyperlinks and hover text in the summaries. For each piece of text, the user test participants w asked for their first set of impressions and then a series of follow-up questions related to content, font, format, language, and anything else.

We revised the summaries according to feedback. The translations were reviewed by a third researcher. The English summaries were presented to 1 more participant in English (moderated, in-person user experience interview) using the same format as the first 4 interviews. The summaries were subsequently revised, and changes were also made to the Norwegian summaries.

We gathered feedback on the Norwegian summaries from colleagues and conducted 2 in-person user experience interviews with 2 native Norwegian speakers using the same question guide as the English user experience interviews. Revisions were made according to the feedback, and where appropriate, these revisions were back translated into the English versions of the COVID-19 trial summaries.

Furthermore, we gathered feedback from the user experience participants on other materials related to the process of participating in the randomized trial (eg, from the invitation to participate to the text sent after participants completed the questionnaire).

### Outcomes

We defined 3 binary coprimary outcomes to measure understanding of overall uncertainty and 1 coprimary outcome to measure understanding of statistical uncertainty (precision of the effect estimate for the benefit). Each of the 3 outcomes for overall uncertainty were measured by comparing participants’ answers to questions (Table 1) about uncertainty to expected (correct) answers based on the size of the effects and the certainty of the evidence (the full questionnaire is provided in the Multimedia Appendix 2).

The 3 *coprimary outcomes* were as follows (all used 4-point ordinal response sets):

- Understanding of the uncertainty of the benefit (question 9)
- Understanding of the sufficiency of the evidence (question 15)
- Understanding of the uncertainty of important harms (question 12)

An additional coprimary outcome was included for the 3 versions reporting the MOE, assessing the understanding of statistical uncertainty (ie, the precision of the effect estimates: choose which among the 4 statements was most consistent with the information provided, eg, question 10: “wearing glasses may *reduce* the chance of COVID a little, but might *reduce it a lot*”).

*Secondary outcomes* included questions (Table 1) regarding the following:

- The perceived benefit and harms of wearing glasses to reduce the chance of acquiring COVID-19 infections (questions 8 and 11)
- Intended behavior (whether participants would wear glasses to reduce the chance of acquiring COVID-19 in areas with high and low COVID-19 infection rates; questions 3 and 7)
- Perceptions of the information; trustworthiness (question 13), sufficiency (question 14), clarity (questions 17 and 18), and helpfulness (question 19); and the likelihood of sharing it with others (question 20)
- Decisional conflict (questions 4 and 5) [28]

**Table 1:** Outcomes and the corresponding questionnaire items.

Outcome		Questionnaire
**Primary outcomes**		
	Understanding uncertainty	Question 9. How sure are you about the effect of wearing glasses on your chance of getting COVID?
	Understanding uncertainty of important harms	Question 12. How sure are you about whether wearing glasses to reduce COVID can cause important harms?
	Understanding sufficiency	Question 15. Not enough is known to be sure about the effects of wearing glasses to reduce the chance of getting COVID.
	Understanding statistical uncertainty	Question 10. Which of the following statements is most consistent with the information provided?
**Secondary outcomes**		
	Perceived benefit and harm	Question 8. What is the possible effect of wearing glasses on your chance of getting COVID? Question 11. How likely do you think it is that wearing glasses to reduce the chance of getting COVID can cause any important harms?
	Intended behavior	Question 3. If there were a surge of COVID-19 cases in your area, how likely would you be to wear glasses or recommend wearing glasses to reduce the chance of getting COVID? Question 7. If there were very few COVID cases in your area, how likely would you be to wear glasses or recommend wearing glasses to reduce the chance of getting COVID?
	Perceptions of the information	Question 13. This information seems like a trustworthy summary of what is known about the effects of wearing glasses to reduce the chance of getting COVID. Question 14. The summary gives me enough information to understand what is known about the effects of wearing glasses to reduce the chance of getting COVID. Question 17. I think the information about whether wearing glasses affects the chance of getting COVID was... Question 18. I think the information about whether wearing glasses to prevent COVID has important harms was... Question 19. If you were making a decision about wearing glasses to prevent COVID, would you find the information we showed you helpful? Question 20. Say you knew someone who heard that wearing glasses might affect your chance of getting COVID. How likely would you be to share the information you just saw with them?
	Decisional conflict	Question 4. The answer about wearing glasses if there were a surge of COVID was hard for me to give. Question 5. The information in the summary helped me make an informed decision about wearing glasses if there were a surge of COVID.

### Data Collection

In the United States, we collected data using SurveyMonkey (SurveyMonkey Inc). Participants were directed to the platform after agreeing to participate in the study. They were asked to enter their personal identifying number (as a panel member on the relevant recruiting platform). In Norway, Opinion collected the data in their own panel via an email invitation and a link to the survey on their own platform. After reading initial information, the participants were randomized to read one of the 6 summaries; they were asked to answer 18 questions about the summary; 4 questions about themselves (eg, whether they wore glasses, what was their highest level of education, and what was their concern regarding COVID-19) to assess saliency of the scenario; and 3 questions to assess their numeracy (Multimedia Appendix 2). They submitted their responses electronically, using the “submit” button (refer to Statistical Analysis section).

### Statistical Analysis

The US and Norwegian trials were analyzed in the same way, except as noted. We excluded participants who completed the survey in <3 minutes, reported regularly wearing glasses, or failed the attention checks. The attention checks (questions 6 and 21) included 2 true or false questions to verify the consistency of participants’ responses, ensuring that they were paying attention and not just randomly answering. Data were duplicated for some US participants who appeared to have submitted the same responses multiple times in a short period. We assumed that these participants clicked the submit button several times or refreshed their browsers, so we analyzed only the first data submitted by these participants. All analyses were performed before unblinding as prespecified according to the intention-to-treat principle—all randomized participants meeting the inclusion criteria were included and analyzed in the arms to which they were randomized [20].

All outcomes were binomial. We used logistic regression to estimate odds ratios (ORs) for the treatments and their interactions. Model fit was assessed using the Hosmer-Lemeshow test. To aid in interpretation, we reexpressed ORs as risk differences, accounting for statistical uncertainty on baseline odds, main effects, and interactions. No data were missing for any of the participants meeting the inclusion criteria.

We used fixed effects meta-analysis to pool estimates across the trials, obtain overall estimates of effect, and assess the country as a potential effect modifier. We performed prespecified subgroup analyses for both trials to explore differences in treatment effect with respect to numeracy and, in the US trial, saliency. Numeracy was defined as scoring 3 (vs <3) on a validated instrument [29]. Saliency was defined as being very or extremely worried about acquiring COVID-19 and considering it very or extremely important to take action to reduce the chance of acquiring COVID-19. It was not possible to perform the analysis for saliency for the Norwegian trial because only 20 participants met the saliency criteria.

Furthermore, we performed nonprespecified analyses for potential effect modification. In the US trial, we explored effect modification by response time (responding within 7 min vs >7 min). The choice of 7 minutes was data driven, chosen to be the whole number of minutes closest to the median response time. We also explored effect modification by education level (having graduated vs not having graduated from a university).

We presented 2-sided 95% CIs and *P* values, wherever applicable, throughout the study. Meta- and subgroup analyses were presented using forest plots, with *P* values testing null hypotheses of homogeneity (there was no difference between the estimates for the 2 trials or no effect modification). All analyses were performed using Stata 18 (StataCorp LLC).

### Ethical Considerations

This study was considered for ethics approval by the Regional Committee for Medical and Health Research Ethics in Norway and was found not to require ethics approval because it falls outside the committee’s mandate under the Health Research Act (reference 557972).

It was also deemed exempt from further review by the Committee for the Protection of Human Subjects (STUDY00032615) and the Dartmouth College Institutional Review Board.

By clicking on the study link in Prolific and Opinions platforms, participants implicitly consented to take part in the study. Everyone invited to participate were given information about who was conducting the research (Dartmouth University was emphasized in the US trial, while Norwegian Institute of Public Health was emphasized in the Norwegian trial), the aim of the research, what they would be asked to do, and how long it would take (Multimedia Appendix 3). It was clarified that participation was voluntary and that participants could discontinue participation at any time. We did not collect personal data from the participants (as per policy of the recruitment agencies), all collected data were anonymous, and we could not trace any data back to the participants. We did not inform the participants how the data would be stored or for how long; however, we did provide contact information to participants to ask any questions.

However, the final process of obtaining informed consent did not completely align with the procedures described in the study protocol [20]. During user testing, we received feedback that the introductory text was too long and cumbersome. Thus, we attempted to shorten it. Given that the study was completely anonymous (no personal or identifying information was gathered from the participants), posed minimal risk, and involved consenting members of the web-based panel platforms we used, we decided that we did not need to provide information regarding how data would be stored. In addition, we could remove a specific sentence asking for informed consent, as implied consent via clicking on the link to participate in the study was deemed sufficient. However, considering emerging best practice guidelines for web-based trials, we will provide more information and seek explicit consent in future trials.

We paid participants 100 Norwegian Kroner (US $8.40) and US $4.30 (50 Norwegian Kroner) in the Norwegian and US trials, respectively.

### Patient and Public Involvement

We involved members of the public in user testing during the development of the survey and COVID-19 trial summaries.

## Results

### Overview

**Figure 3:**
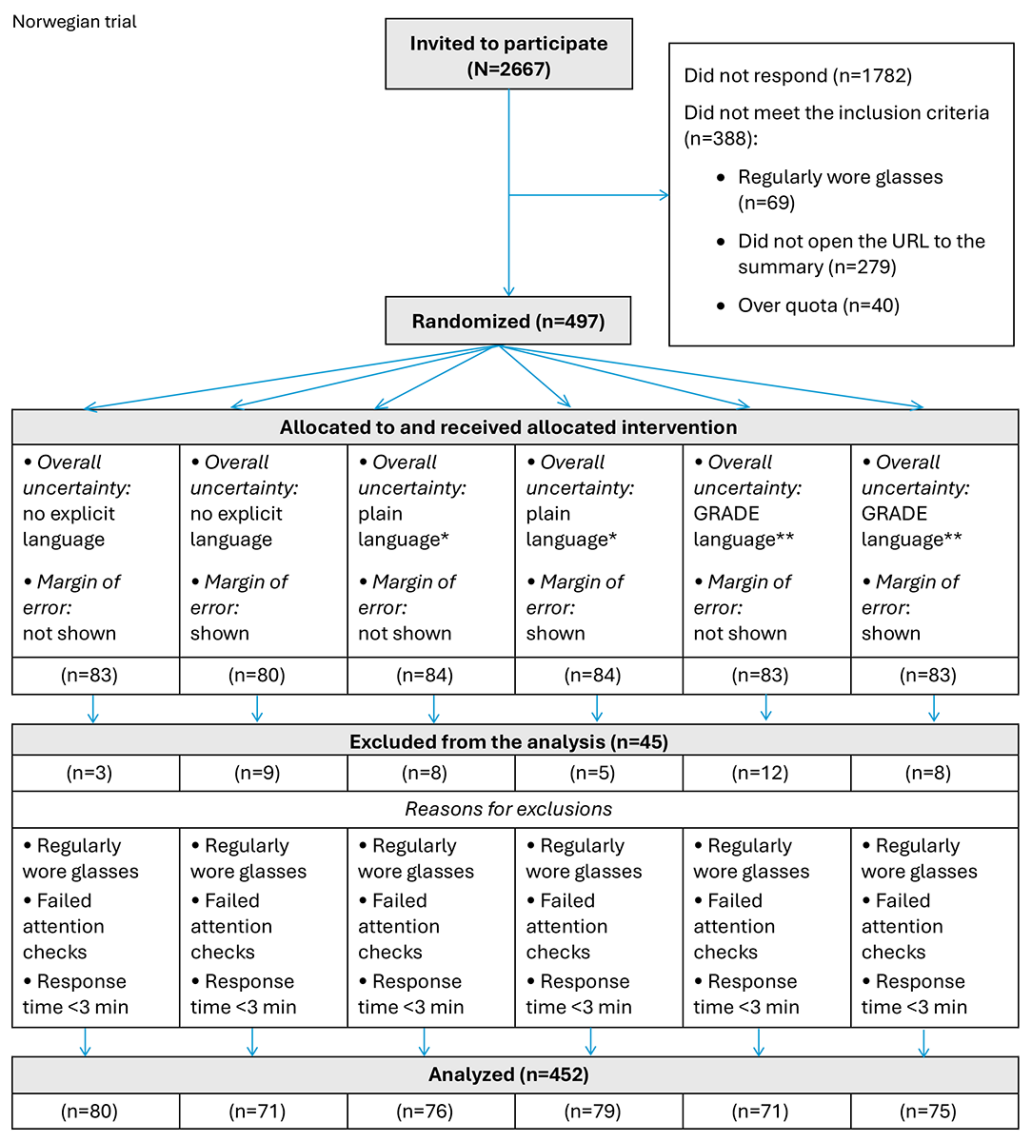
Modified CONSORT (Consolidated Standards of Reporting Trials) flow diagram of the participant inclusion process in April 2023. The flow diagram shows the eligible, recruited, and allocated participants from the Opinion panel in the Norwegian randomized controlled trial. *A less formal expression of the overall uncertainty language used in ordinary or familiar conversation, corresponding to the same Grading of Recommendations Assessment, Development and Evaluation (GRADE) assessment of the certainty of the evidence. **On the basis of the Cochrane Effective Practice and Organization of Care Group’s guidance for communicating the certainty of evidence based on the GRADE approach to assessing the certainty of evidence [30].

We invited 2667 Norwegians to participate in the trial, of which 1782 (66.82%) did not respond, 388 (14.55%) were not eligible, and 497 (18.63%) were randomized to one of the 6 intervention groups (Figure 3).

Of the 497 participants, 45 (9%) were excluded from the analysis for the reasons provided in Figure 3.

**Figure 4:**
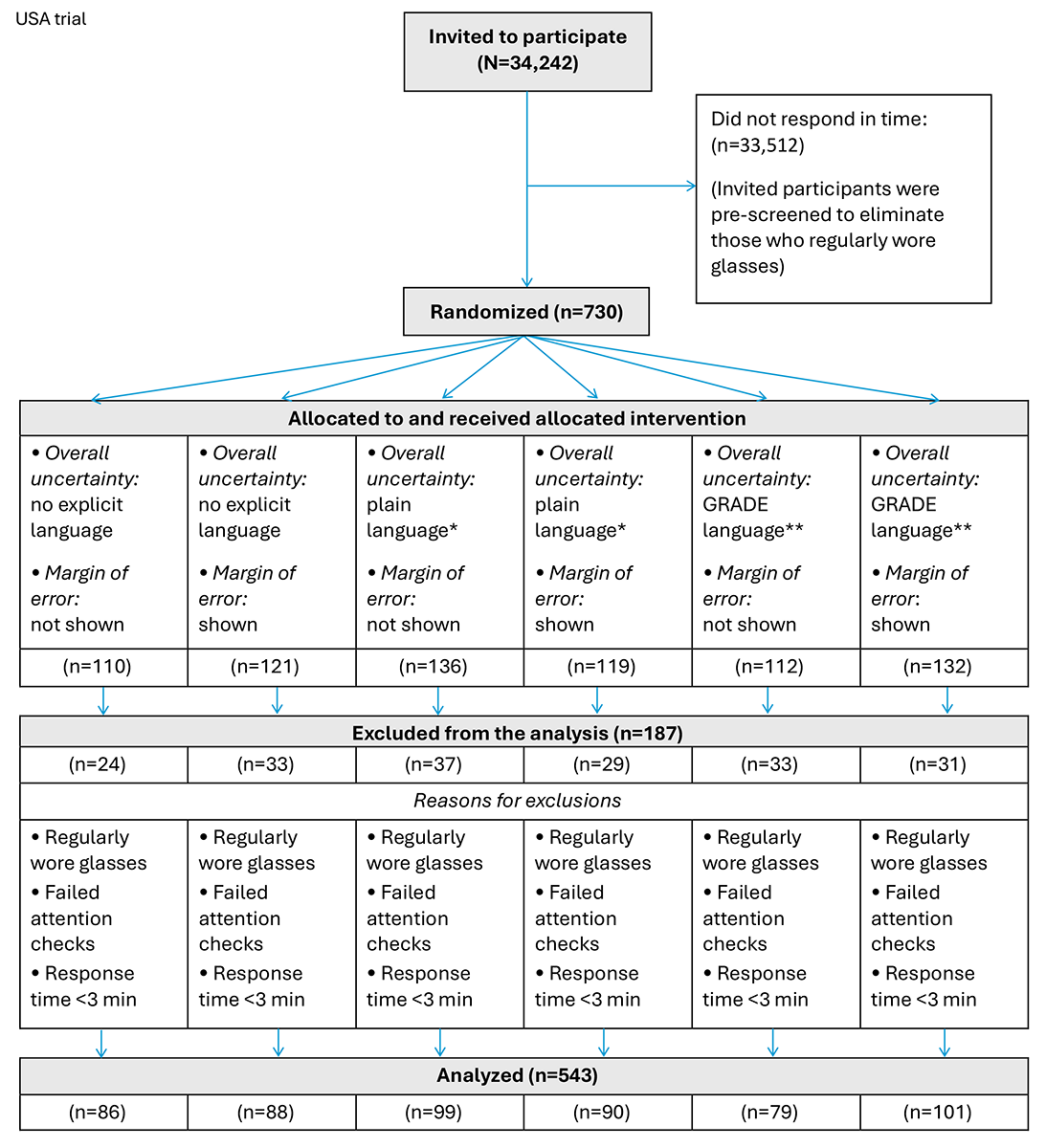
Modified CONSORT (Consolidated Standards of Reporting Trials) flow diagram of the participant inclusion process in April 2023. The flow diagram shows the eligible, recruited, and allocated participants from the Prolific panel in the US randomized controlled trial. *A less formal expression of the overall uncertainty language used in ordinary or familiar conversation, corresponding to the same Grading of Recommendations Assessment, Development and Evaluation (GRADE) assessment of the certainty of the evidence. **On the basis of the Cochrane Effective Practice and Organization of Care Group’s guidance for communicating the certainty of evidence based on the GRADE approach to assessing the certainty of evidence [30].

The study was open for 34,242 people in the United States to participate in the trial, of which 33,512 (97.88%) did not respond in time and 730 (2.13%; required sample size) were randomized to one of the 6 intervention groups (Figure 4).

Of the 730 participants, 187 (25.6%) were later excluded from the analysis for the reasons provided in Figure 4. The average age of participants was 45 (SD 16.8) years in the Norwegian trial and 38 (SD 13.2) years in the US trial (Table 2).

In total, 50% (226/452) of the Norwegians and 48.3% (262/543) of the US participants were female. The educational level was higher in the US participants (284/543, 52.3% had at least a college degree) than in the Norwegian participants (197/452, 43.6% had at least a college degree). Altogether, approximately half (517/995, 52%) of participants in both trials failed to answer all 3 numeracy questions correctly (Table 2).

There were only minor differences among the comparison groups in both the Norwegian and US trials (Tables S1 and S2 in Multimedia Appendix 4).

**Table 2:** Characteristics of the Norwegian and American participants from the web-based research panels taking part in the randomized controlled trial on evaluating the people’s understanding of overall and statistical uncertainty when presented with alternative expressions in April 2023.

			Norwegian trial (n=452)		US trial (n=543)		Total (N=995)
Age (y), mean (SD)			45 (16.8)		38 (13.2)		41 (15.4)
**Age group (y), n (%)**							
	18 to 29	109 (24.1)		155 (28.5)		264 (26.5)	
	30 to 39	74 (16.4)		195 (35.9)		269 (27)	
	40 to 49	81 (17.9)		98 (18)		179 (18)	
	50 to 59	88 (19.5)		27 (5)		115 (11.6)	
	60 to 69	59 (13.1)		58 (10.7)		117 (11.8)	
	≥70	41 (9.1)		10 (1.8)		51 (5.1)	
**Sex, n (%)**							
	Female	226 (50)		262 (48.3)		488 (49)	
	Male	226 (50)		281 (51.7)		507 (51)	
**Employment status, n (%)**							
	Full time	209 (46.2)		213 (39.2)		422 (42.4)	
	Part time	34 (7.5)		65 (12)		99 (9.9)	
	Unpaid^a^	—^b^		55 (10.1)		55 (5.5)	
	Unemployed or seeking work	—		51 (9.4)		51 (5.1)	
	Welfare^c^	114 (25.2)		—		114 (11.5)	
	Self-employed	22 (4.9)		—		22 (2.2)	
	Missing	17 (3.8)		129 (23.8)		146 (14.7)	
	Student	49 (10.8)		—		49 (4.9)	
	Other	7 (1.5)		30 (5.5)		37 (3.7)	
**Highest education, n (%)**							
	<High school	33 (7.3)		25 (4.6)		58 (5.8)	
	High school degree	131 (29)		92 (16.9)		223 (22.4)	
	Some college	91 (20.1)		142 (26.2)		233 (23.4)	
	College degree	124 (27.4)		204 (37.6)		328 (33)	
	Graduate or professional school	73 (16.2)		80 (14.7)		153 (15.4)	
**Numeracy, n (%)**							
	<3	235 (52)		282 (51.9)		517 (52)	
	3	217 (48)		261 (48.1)		478 (48)	

^a^Includes participants who are homemakers, retired, or disabled.

^b^Not applicable.

^c^Includes participants in any welfare program, maternity leave, pensioner, or unemployment benefits.

### Understanding of the Uncertainty of the Benefit

#### Overall

**Figure 5:**
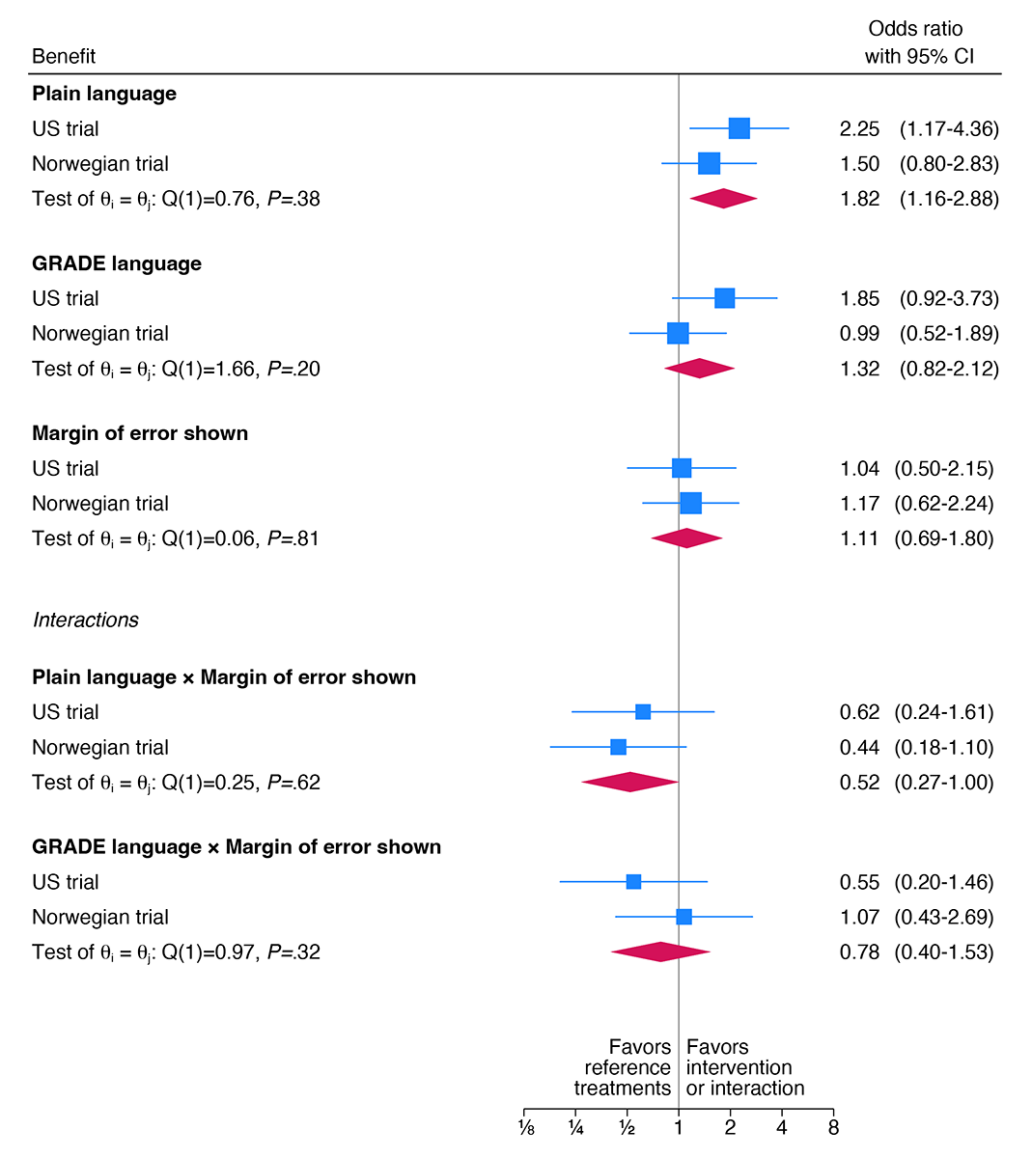
Meta-analysis of the understanding of the uncertainty of the benefit of wearing glasses. Odds ratios for answering “Mixed but more unsure than sure” to the question “How sure are you about the effect of wearing glasses on your chance of getting COVID?” in the Norwegian and US randomized controlled trials on evaluating people’s understanding of overall and statistical uncertainty when presented with alternative expressions. GRADE: Grading of Recommendations Assessment, Development and Evaluation.

Overall, plain (but not GRADE) language, compared to no explicit language, improved correct understanding of the overall uncertainty of the effect of wearing glasses on the chance of acquiring COVID-19 (ie, “mixed but more unsure than sure;” OR 1.82, 95% CI 1.16-2.88; Figure 5).

When the MOE was shown together with plain language, the effect on understanding of the overall uncertainty was substantially reduced (OR 0.52, 95% CI 0.27-1.00; Figure 5): participants were more likely to perceive the evidence as “very unsure” rather than “mixed but more unsure than sure” (the correct response) (Table S3 and S4 in Multimedia Appendix 4).

#### Individual Trials

Overall, on average, across all 6 comparison groups, there were 25.2% (114/452) of Norwegian participants who responded that the evidence was very unsure compared to US participants, of whom 16.4% (89/543) reported the same (Table S3 and S4 in Multimedia Appendix 4).

The risk difference for the Norwegian trial was less certain (risk difference of 10.1%, 95% CI –5.5 to 25.7; Table 3) than in the US trial.

In the US trial, using plain language increased correct understanding of how sure we can be about the effect of wearing glasses on the chance of acquiring COVID-19, from 21% (18/86) to 37% (37/99; risk difference 16.4%; 95% CI 3.6-29.3; Table 4).

**Table 3:** Risk differences for the understanding of the overall uncertainty of the benefit of wearing glasses in the Norwegian randomized controlled trial on evaluating people’s understanding of overall and statistical uncertainty when presented with alternative expressions for the question “How sure are you about the effect of wearing glasses on your chance of getting COVID?” with responses including very sure, mixed but more sure than unsure, mixed but more unsure than sure (correct), and very unsure.

Uncertainty	Margin of error	Participants^a^, n (%)	Odds ratio^b^ (95% CI)	Risk difference^c^ (%), (95% CI)	*P* value
No explicit language (n=80)	Not shown	34 (42.5)	1	0	—^d^
No explicit language (n=71)	Shown	33 (46.5)	1.17 (0.62 to 2.24)	4.0 (–11.89 to 19.85)	.62
Plain language (n=76)	Not shown	40 (52.6)	1.50 (0.80 to 2.83)	10.1 (–5.47 to 25.73)	.21
Plain language (n=79)	Shown	29 (36.7)	0.78 (0.42 to 1.48)	–5.8 (–20.97 to 9.39)	.46
GRADE^e^ language (n=71)	Not shown	30 (42.3)	0.99 (0.52 to 1.89)	–0.3 (–16.04 to 15.54)	.98
GRADE language (n=75)	Shown	36 (48)	1.25 (0.66 to 2.35)	5.5 (–10.16 to 21.16)	.49

^a^Participants randomized to the intervention answering correctly or as anticipated.

^b^Odds ratios include the main and interaction effects.

^c^Risk differences account for uncertainty on the baseline odds.

^d^Not applicable.

^e^GRADE: Grading of Recommendations Assessment, Development and Evaluation.

**Table 4:** Risk differences for the understanding of the overall uncertainty of the benefit of wearing glasses in the US randomized controlled trial on evaluating people’s understanding of overall and statistical uncertainty when presented with alternative expressions for the question “How sure are you about the effect of wearing glasses on your chance of getting COVID?” with responses including very sure, mixed but more sure than unsure, mixed but more unsure than sure (correct), and very unsure.

Overall uncertainty	Margin of error	Participants^a^, n (%)	Odds ratio (95% CI)^b^	Risk difference^c^ (%), (95% CI)	*P* value
No explicit language (n=86)	Not shown	18 (20.9)	1	0	—^d^
No explicit language (n=88)	Shown	19 (21.6)	1.04 (0.50 to 2.15)	0.7 (–11.5 to 12.8]	.92
Plain language (n=99)	Not shown	37 (37.4)	2.25 (1.17 to 4.36)	16.4 (3.6 to 29.3)	.02
Plain language (n=90)	Shown	25 (27.8)	1.45 (0.73 to 2.91)	6.8 (–5.8 to 19.5)	.29
GRADE^e^ language (n=79)	Not shown	26 (32.9)	1.85 (0.92 to 3.73)	12.0 (–1.5 to 25.4)	.08
GRADE language (n=101)	Shown	22 (21.8)	1.05 (0.52 to 2.12)	0.9 (–10.9 to 12.6)	.89

^a^Participants randomized to the intervention answering correctly or as anticipated.

^b^Odds ratios include the main and interaction effects.

^c^Risk differences account for uncertainty on the baseline odds.

^d^Not applicable.

^e^GRADE: Grading of Recommendations Assessment, Development and Evaluation*.*

### Understanding of the Sufficiency of the Evidence

#### Overall

**Figure 6:**
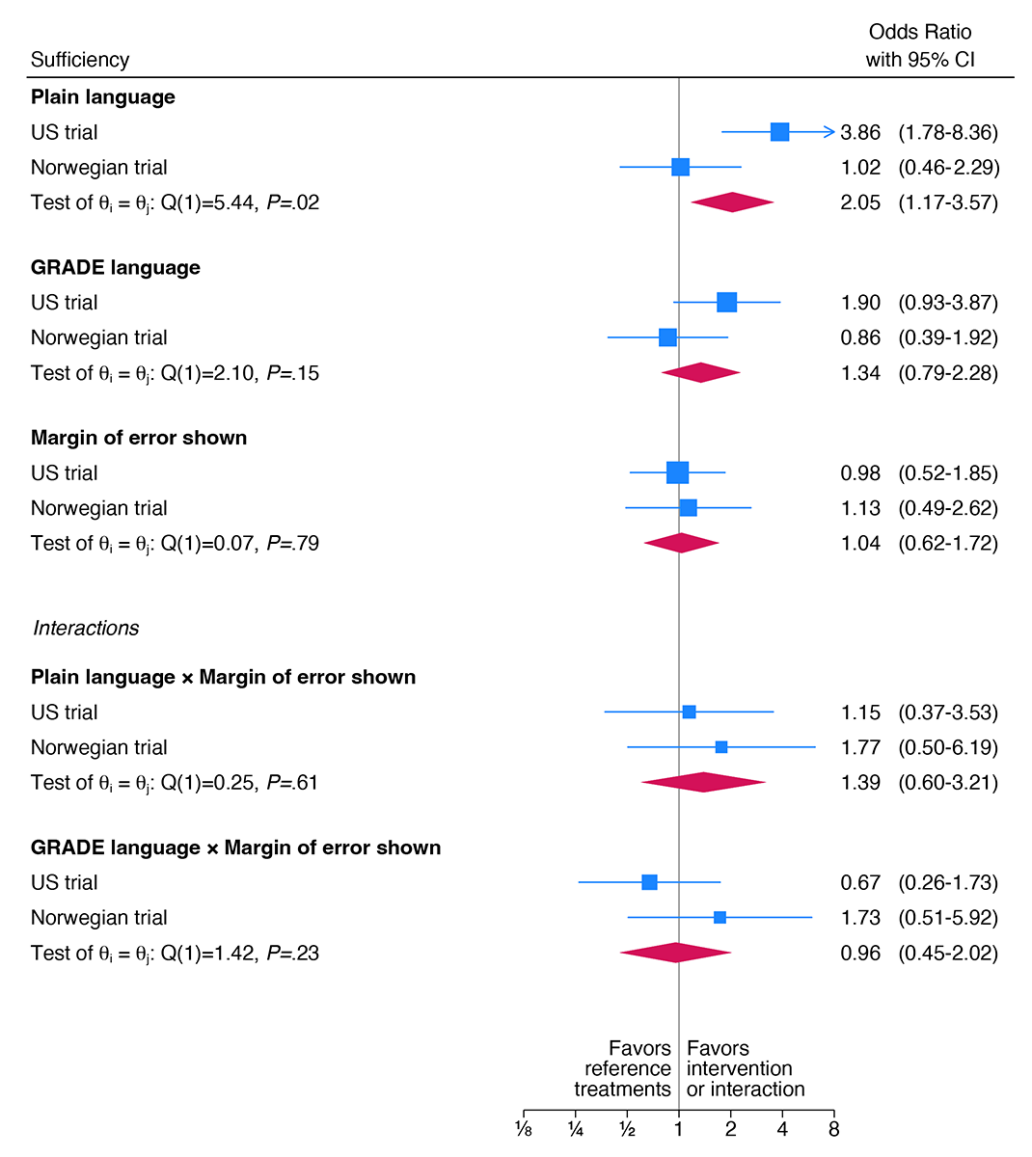
Meta-analysis presenting understanding the sufficiency of the evidence. Odds ratios for agreeing or strongly agreeing with “Not enough is known to be sure about the effects of wearing glasses to reduce the chance of getting COVID” in the Norwegian and US randomized controlled trials on evaluating people’s understanding of overall and statistical uncertainty when presented with alternative expressions. GRADE: Grading of Recommendations Assessment, Development and Evaluation.

Overall, plain, compared to no explicit language, improved correct understanding of the sufficiency of the evidence (OR 2.05, 95% CI 1.17-3.57; Figure 6). However, the results of the 2 trials were heterogeneous (Cochran Q_1_=5.44; *P*=.02). The OR for the Norwegian trial was 1.02 (95% CI 0.46-2.29), whereas the OR for the US trial was 3.86 (95% CI 1.78-8.36). The effect of GRADE language, compared to no explicit language, was inconclusive on understanding of the sufficiency of the evidence (OR 1.34, 95% CI 0.79-2.28; Figure 6).

#### Individual Trials

The results of the Norwegian trial for this outcome likely reflected a ceiling effect. In total, 81% (65/80) of the participants, who were shown no explicit language for overall uncertainty and no MOE (Table 5), agreed that not enough was known to be sure about the effects of wearing glasses to reduce the chance of acquiring COVID-19.

On the basis of the results of the US trial, using plain language increased the proportion of participants who agreed or strongly agreed that not enough was known to be sure about the effects of wearing glasses to reduce the chance of getting COVID-19, from 67% (58/86) to 89% (88/99); risk difference 21.4%, 95% CI 9.8-33.1 (Table 6). The effect of using the GRADE language was less certain.

Participants randomized to the intervention answering correctly or as anticipated.

**Table 5:** Risk differences for sufficiency of the evidence in the Norwegian randomized controlled trial on evaluating people’s understanding of overall and statistical uncertainty when presented with alternative expressions for the question “not enough is known to be sure about the effects of wearing glasses to reduce the chance of getting COVID,” with responses including strongly agree, agree (correct), disagree, and strongly disagree.

Overall uncertainty	Margin of error	Participants^a^, n (%)	Odds ratio (95% CI)^b^	Risk difference^c^ (%; 95% CI)	*P* value
No explicit language (n=80)	Not shown	65 (81.2)	1	0	—^d^
No explicit language (n=71)	Shown	59 (83.1)	1.13 (0.49 to 2.62)	1.9 (–10.36 to 14.06)	.77
Plain language (n=76)	Not shown	62 (81.6)	1.02 (0.46 to 2.29)	0.3 (–11.88 to 12.54)	.96
Plain language (n=79)	Shown	71 (89.9)	2.05 (0.81 to 5.15)	8.6 (–2.21 to 19.46)	.13
GRADE^e^ language (n=71)	Not shown	56 (78.9)	0.86 (0.39 to 1.92)	–2.4 (–15.16 to 10.40)	.72
GRADE language (n=75)	Shown	66 (88)	1.69 (0.69 to 4.14)	6.8 (–4.53 to 18.03)	.25

^a^Participants randomized to the intervention answering correctly or as anticipated.

^b^Odds ratios include the main and interaction effects.

^c^Risk differences account for uncertainty on the baseline odds.

^d^Not applicable.

^e^GRADE: Grading of Recommendations Assessment, Development and Evaluation*.*

**Table 6:** Risk differences for sufficiency of the evidence in the US randomized controlled trial on evaluating people’s understanding of overall and statistical uncertainty when presented with alternative expressions for the question “not enough is known to be sure about the effects of wearing glasses to reduce the chance of getting COVID,” with responses including strongly agree, agree (correct), disagree, and strongly disagree.

Overall uncertainty	Margin of error	Participants^a^, n (%)	Odds ratio (95% CI)^b^	Risk difference^c^ (%; 95% CI)	*P* value
No explicit language (n=86)	Not shown	58 (67.4)	1	0	—^d^
No explicit language (n=88)	Shown	59 (67)	0.98 (0.52 to 1.85)	–0.4 (–14.34 to 13.55)	.96
Plain language (n=99)	Not shown	88 (88.9)	3.86 (1.78 to 8.36)	21.5 (9.77 to 33.13)	<.001
Plain language (n=90)	Shown	81 (90)	4.34 (1.91 to 9.90)	22.6 (10.87 to 34.24)	<.001
GRADE^e^ language (n=79)	Not shown	63 (79.7)	1.90 (0.93 to 3.87)	12.3 (–0.98 to 25.59)	.08
GRADE language (n=101)	Shown	73 (72.3)	1.26 (0.67 to 2.36)	4.8 (–8.37 to 18.04)	.47

^b^Odds ratios include the main and interaction effects.

^c^Risk differences account for uncertainty on the baseline odds.

^d^Not applicable.

^e^GRADE: Grading of Recommendations Assessment, Development and Evaluation*.*

### Understanding of the Uncertainty of Important Harms

#### Overall

There was no difference between plain and GRADE languages, compared to no explicit language, on the ability to correctly understand the uncertainty of important harms (OR 0.98, 95% CI 0.68-1.63 and OR 1.23, 95% CI 0.85-1.78; see Figure S1 in Multimedia Appendix 4).

#### Individual Trials

Understanding of the uncertainty of important harms varied across the trials. In total, 14.2% (64/452) of the participants in the Norwegian trial and 31.1% (169/543) in the US trial correctly understood what we aimed to communicate about the certainty of the evidence for important harms (Tables S5 and S6 in Multimedia Appendix 4). In both trials, there were only small differences between the comparison groups that could have occurred by chance alone.

### Understanding Statistical Uncertainty (MOE)

#### Overall

In both countries, showing, compared to not showing, the MOE increased the proportion of people who chose the answer most consistent with the information provided (may reduce the chance of COVID a little but might increase it a little).

#### Individual Trials

In the Norwegian trial, 27.1% (61/225 and between 21%-34% in each of the 3 groups) of the participants answered correctly when the MOE was shown. Only 1.8% (4/227 and between 1%-3% in each of the 3 groups) answered correctly when MOE was not shown. In the US trial, 27.6% (77/279 and between 21%-36% in each of the 3 groups)) answered correctly when MOE was shown, while 1.9% (5/264 and between 0%-4% in each of the 3 groups) answered correctly when the MOE was not shown (Tables S7 and S8 in Multimedia Appendix 4).

The majority of the total number of participants shown the MOE (366/504, 72.6%) from both trials (and between 64% and 79% in each of the 3 groups) *failed* to correctly understand the MOE. As expected, very few people correctly guessed the MOE when it was not shown. A correct answer when not shown the MOE would have been “Don’t know.” The proportion of participants who responded “Don’t know” when the MOE was shown was 4.8% (24/504) compared to 4.7% (23/491) when MOE was not shown.

### Secondary Outcomes

#### Interest in Wearing Glasses to Reduce COVID-19 Risk During a Surge in Cases

Interest in wearing glasses to reduce COVID-19 risk during a surge was consistently lower in the Norwegian versus US trials (Figure S2 in Multimedia Appendix 4). In the Norwegian trial, plain language with or without the MOE and the GRADE language without the MOE reduced the proportion of participants who responded that they would be likely or very likely to wear or recommend wearing glasses to reduce the chance of acquiring COVID-19 if there were a surge of COVID-19 cases (risk difference: –13.6%, 95% CI –24.7 to –2.5; –18.6%, 95% CI –28.7 to –8.4; and −15.5%, 95% CI –26.4 to –4.5, respectively; Table S9 in Multimedia Appendix 4).

In the US trial, the combination of using either plain or GRADE languages to communicate the overall uncertainty of the benefit of wearing glasses and showing the MOE reduced the proportion of participants who responded that they would be likely or very likely to wear or recommend wearing glasses to reduce the chance of acquiring COVID-19 if there were a surge of COVID-19 cases (risk difference: –20.5%, 95% CI –33.1 to –7.9 and 21.2%, 95% CI –33.5 to –8.9, respectively; Table S10 in Multimedia Appendix 4).

The difference between the 2 trials is in part due to the lower proportion of participants in the Norwegian reference group (18/80, 23%) compared to the US reference group (31/86, 36%), who responded that they would be likely or very likely to wear or recommend wearing glasses to reduce the chance of acquiring COVID-19 if there were a surge of COVID-19 cases. This might be due to the translation from English to Norwegian.

#### Interest in Wearing Glasses to Reduce COVID-19 Risk if No Surge in Cases

We did not find an effect of plain or GRADE languages, compared to no explicit language, on the likelihood of wearing glasses or recommending wearing glasses to reduce the chance of acquiring COVID-19 if there were very few COVID-19 cases, the perceived benefit of wearing glasses, or the perceived chance of important harms (Figure S3 in Multimedia Appendix 4).

#### Perceptions of the Information Provided

In both trials, perceptions of the information provided differed little across plain and GRADE languages compared to no explicit language, with few exceptions; in both trials (US trial more so than Norway), perceptions of *helpfulness* were somewhat lower with plain language, with or without the MOE (Tables S11 and S12 in Multimedia Appendix 4).

In the Norwegian trial, with plain or GRADE languages, reporting the MOE reduced perceptions of the *trustworthiness* of the information (Table S13 and S14 in Multimedia Appendix 4).

In the Norwegian trial, the GRADE language without the MOE reduced the perception that information about whether wearing glasses affects the chance of acquiring COVID-19 was *sufficient* (Table S15 and S16 in Multimedia Appendix 4).

In the Norwegian trial, plain language without the MOE and the GRADE language with it reduced perceptions that the information about the benefit of glasses was *clear.* A similar effect was seen in the US trial for plain language with the MOE (Tables S17 and S18 in Multimedia Appendix 4).

In the Norwegian trial, plain language with the MOE reduced perceptions that information about whether glasses had important harms was *clear*. Similarly, this effect was seen in the US trial when the MOE was shown with no explicit language or plain language (Tables S19 and S20 in Multimedia Appendix 4).

In the Norwegian trial, plain language with the MOE reduced the proportion of participants who responded that they definitely or probably would *share the information* with someone who heard that wearing glasses might affect the chance of acquiring COVID-19 (Table S21 and S22 in Multimedia Appendix 4).

#### Decisional Conflict

Plain language without the MOE increased the feeling that the decision about wearing glasses if there were a surge of COVID-19 cases was hard to make (Table S23 and S24 in Multimedia Appendix 4). Most participants felt that they made an informed decision about wearing glasses if there were a surge of COVID-19 (264/452, 58.4%; between 52.6% and 67.6% in the Norwegian trial and 406/543, 74.8%; between 70.9% and 83% in the US trial; Table S25 and S26 in Multimedia Appendix 4).

#### Potential Modifying Factors

We did not find credible evidence of effect modification for numeracy, education, salience, or the time taken to complete the questionnaire.

## Discussion

### Principal Findings

The US and Norwegian trials comparing different ways of communicating the overall and statistical uncertainty of research results to the public generated mixed results. Plain language improved readers’ understanding of the overall uncertainty of the benefit but only to a modest extent. The effect of the GRADE language was uncertain but, at best, had a modest effect. Furthermore, reporting the MOE reduced understanding by making the evidence seem more uncertain than it actually was. Reporting the MOE did improve understanding of statistical uncertainty around the effect of glasses but only for a minority group of people. A more detailed discussion of the most important findings follows in the subsequent sections.

### Communicating Overall Uncertainty of the Benefit

Plain language is probably more accessible than the GRADE language (eg, the phrase “not very confident” seems easier to understand than “moderate uncertainty”) for communicating the overall uncertainty of benefit. Given the modest findings, more work is needed on the language; other ways of communicating uncertainty, such as visualizations; and easily accessible explanations.

It should be noted that we displayed the GRADE symbol together with the GRADE language. The GRADE symbols for uncertainty are similar to widely used symbols used for ranking the quality of, for example, hotels, restaurants, and consumer products [30]. Nonetheless, they may be unfamiliar in this context and not easily understood without further explanation.

In another small trial [31], the GRADE symbols were compared to letters to convey the quality of evidence, and both letters and symbols were well understood.

Both the plain language and the GRADE language summaries included text explaining why we were “not very confident” (it was “uncertain” that wearing glasses may slightly reduce the chance of acquiring COVID-19). These explanations were available under a tab labeled “Keep in mind.” Neither the number of participants who read this text nor the effect of the text on their understanding of the uncertainty of the evidence is known. More Norwegians responded that the evidence was very unsure compared to US participants. One reason for the differences between the Norwegian and US results may be that “may reduce” was translated to “kan muligens redusere” (“can possibly reduce”) in Norwegian (based on the approved translation used by the Norwegian Institute of Public Health) [32]. The word “kan” in Norwegian can mean either “may” or “can,” and these 2 terms may differ with regards to their emphasis on the degree of uncertainty, which is why the moderating adverb “muligens” (directly translating to “possibly”) was included in the Norwegian version. Although we user-tested the summaries before the trials, further exploration of the extent to which people find this text helpful and how it impacts their understanding of the uncertainty of the evidence is warranted. Furthermore, cultural understandings and comfort with terms related to or describing the concept of “uncertainty” could be further explored to communicate uncertainty more accurately in languages other than English.

### Communicating Overall Uncertainty of Important Harm

Neither plain language nor the GRADE language improved the understanding of the overall uncertainty of important harm. This could be because we did not explain why we said that there was moderate certainty evidence and prima facie, it seemed implausible to participants that wearing glasses could cause important harm. In fact, about one-third of the Norwegian participants were very sure, and this might be because they considered it implausible that wearing glasses could cause important harm. On the other hand, the other participants (about half) might have been somewhat or very unsure because there was so little information. The difference between the Norwegian and US trials might have been due to translation. The English version for the control group (No explicit language) stated that “wearing glasses probably does not cause important harms, such as serious fall due to reduced vision” while the Norwegian version, if back translated stated “Using glasses probably doesn’t cause serious injury, for example after falling” but does not refer to “reduced vision.” Roughly twice as many participants in the US trial, compared to participants in the Norwegian trial, correctly understood the overall uncertainty of the evidence for important harm. The most likely reason for this is the difference between the English summaries and the Norwegian translations. In this case, the explanation for why important harms (serious injury after falling is plausible because of reduced vision) was not mentioned in the Norwegian translation. This highlights the need for more extensive user testing and the use of back translation or other means of ensuring that translations are correctly understood.

### Effect of MOE on Overall Uncertainty

Showing the MOE did help people understand statistical uncertainty (ie, glasses may reduce the chance of acquiring COVID-19 a little *but might* increase it a little). While this is encouraging, the modest effect suggests more work is needed to ensure that people understand what MOE implies.

In the group of participants who were shown plain language, showing the MOE (for benefit) decreased the proportion of people who answered this question correctly. This probably happened because the effect of glasses was small, and the MOE really highlighted that adding uncertainty to uncertainty in the context of a small effect, to begin with, made people feel the effect was less certain than it was. This finding underscores the need for more work to help people calibrate their sense of uncertainty. It would be interesting to see what would happen in another example where the intervention effect was bigger than the effect of glasses. Another reason for this may be that participants who were shown the MOE were also shown 2 reasons for rating the evidence as low certainty (wide MOE *and* important study limitations), whereas those who were not shown the MOE were only presented with 1 source of uncertainty (important study limitations). Findings from a 2020 study [33] looking at the effect of communicating uncertainty found that participants who were shown 3 sources of uncertainty were more likely to report a weaker perception of the effectiveness of the intervention (drug) than those who were presented with only 2 sources of uncertainty. More research needs to be conducted to explore this hypothesis.

### Effect of MOE on Intended Behavior

Reporting overall uncertainty using plain language or the GRADE language or reporting the MOE decreased the likelihood that participants would wear glasses to protect themselves against COVID-19 if there was a surge in cases. This finding is consistent with other research findings that suggest reporting uncertainty decreased the likelihood that people would use eye protection to reduce their chance of acquiring COVID-19 [18]. It is also logical that the less certain one is about the benefits of doing something, the less likely it is to be done. These results highlight the importance of effectively communicating uncertainty if the intention is to inform people rather than to persuade them [19].

### Effect of MOE on Perceptions of Information

In Norwegian trial, showing the MOE in combination with plain language reduced the perceived trustworthiness of the information (the results were similar, although smaller, and not statistically significant for the other versions). This is consistent with the findings of Schneider et al [18], which found that including a clue that evidence of the effect of eye protection was low quality or certainty reduced the perceived trustworthiness of an infographic. In contrast, in our US trial, the reduction in perceived trustworthiness when showing the MOE was substantially smaller and not statistically significant in any version. This suggests that we cannot conclude that communicating uncertainty necessarily reduces the perceived trustworthiness of information about the effects of interventions. Indeed, more candid communication over time might make changes in recommendations seem less arbitrary and help preserve people’s trust in health authorities [14].

There is some evidence that plain language with or without the MOE and the GRADE language with the MOE reduced the perception that the summary was sufficient. This may be due to the participants confusing the sufficiency of the evidence (ie, a small, uncertain effect) with the sufficiency of the summary, which was meant to communicate the effect. While the question we asked specifically aimed at the latter (ie, whether the information was a sufficient summary of what is known about the effects of wearing glasses to reduce the chance of acquiring COVID-19), future qualitative work should be done to help distinguish these 2 kinds of sufficiency. Similarly, showing the MOE also reduced the perception that the summaries were clear, perhaps for the same reasons. In the Norwegian trial, the likely ceiling effect may reflect the mentioned translation of “may” to “can possibly” in the Norwegian summary.

Nevertheless, most participants found the decision about wearing glasses to reduce the chance of acquiring COVID-19 hard to make, with or without the MOE, regardless of which summary they were shown.

### Limitations

Study limitations include the weak, uncertain effect that we were trying to summarize (from the Glasses trial), which magnified the communication challenges, and several language translation issues, which may limit the cross-country comparisons. Furthermore, our results may have been influenced by a lack of saliency, that is, COVID-19 infection rates were relatively low when we conducted the study, and participants might have responded differently in a more realistic or pressing scenario. Furthermore, we need to explore the effect of paying participants in web-based trials on the quality of the responses [24].

As this study was the research team’s first attempt at conducting a web-based trial, we encountered and reflected on a number of challenges and opportunities. First, while we were satisfied with the web-based platforms we eventually chose, it was time-consuming and challenging to identify a proper platform to facilitate the conduct of the trial, and we eventually needed to incorporate 2 platforms to meet our specific needs. Another challenge was related to obtaining informed consent—it was unclear what elements of the consent were covered by the web-based platform or our invitation text. Since the conduct of our trial, more guidance has been provided on the platforms and other academic websites offering guidance on informed consent in web-based trials.

In the next Message Lab trial for assessing different ways of communicating evidence, we would like to set up Google Analytics (Google LLC), if using Google Sites to present the health messages to the participants. This will allow us to investigate further on how much time people spend on the different sections and how they navigate the texts presented.

### Conclusions

Our study has several strengths, including the randomized factorial design, which let us explore the interaction between uncertainty language (GRADE, plain, or no explicit) and the MOE on understanding the benefits, harms, and the corresponding uncertainties and allowed replication in 2 distinct populations. Our study shows that explicitly reporting uncertainty affects peoples’ understanding, perceptions, and intended actions. We found that plain language was better than no explicit language in helping people understand the overall uncertainty of the evidence, although most participants still did not correctly understand how sure they could be. Reporting MOE reduced understanding of the overall uncertainty by making people feel that the evidence was even less certain. Reporting MOE improved the interpretation of statistical uncertainty around the effect of glasses, but only for a minority group of participants.

This study underscores how much more work needs to be done to develop effective ways to communicate overall uncertainty and just how unclear the numbers are (statistical uncertainty). If this communication is done poorly, it may simply add to confusion and lead to poor decisions. If done properly, effective communication around uncertainty can help people to make the best decisions they can, given the evidence that is known.

## Appendix

Multimedia Appendix 1 All summaries in both English and Norwegian.

Multimedia Appendix 2 Questionnaires in English and Norwegian.

Multimedia Appendix 3 Information provided to the participants.

Multimedia Appendix 4 Additional result tables and figures.

Multimedia Appendix 5 CONSORT-eHEALTH checklist (V 1.6.1).

## Abbreviations

GRADE: Grading of Recommendations Assessment, Development and Evaluation
MOE: margin of error
OR: odds ratio

## Data Availability

The datasets generated and analyzed during this study are available from the corresponding author upon reasonable request.
