# Supplementary material for: Alternative Presentations of Overall and Statistical Uncertainty for Adults’ Understanding of the Results of a Randomized Trial of a Public Health Intervention: Parallel Web-Based Randomized Trials
Source: JMIR Public Health Surveill. 2025 Mar 18;11:e62828. doi: 10.2196/62828 (PMC11962331; doi:10.2196/62828)
Supplement: Multimedia Appendix 1 [file publichealth_v11i1e62828_app1.pdf]

Multimedia appendix 1 – All summaries, both languages

English summaries

Version1: No explicit language, margin of error not shown (reference / control version)

https://sites.google.com/view/glasses-summary-6/home

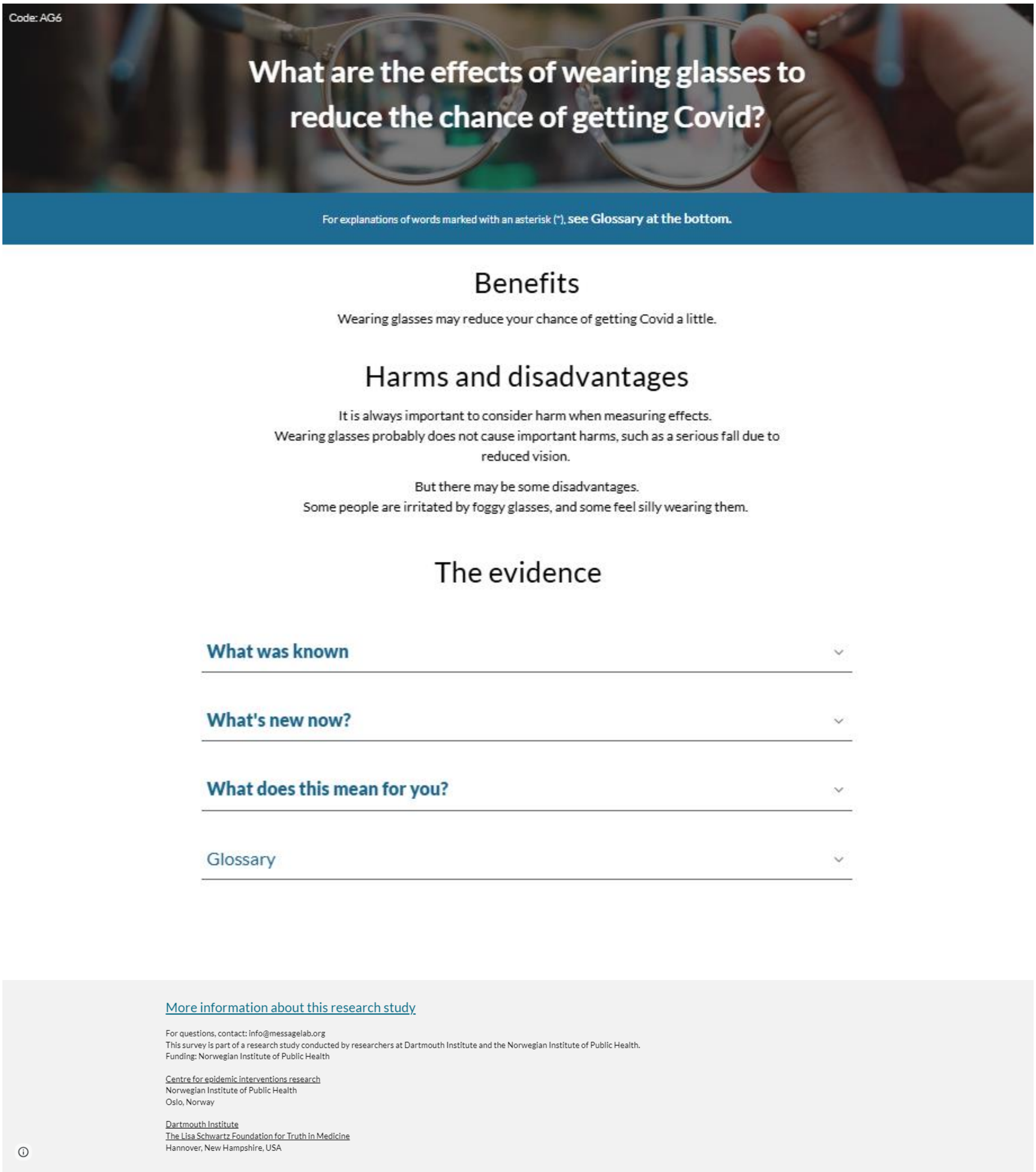

Code: AG6

What are the effects of wearing glasses to reduce the chance of getting Covid?

For explanations of words marked with an asterisk (*), see Glossary at the bottom.

Benefits

Wearing glasses may reduce your chance of getting Covid a little.

Harms and disadvantages

It is always important to consider harm when measuring effects.
Wearing glasses probably does not cause important harms, such as a serious fall due to reduced vision.

But there may be some disadvantages.
Some people are irritated by foggy glasses, and some feel silly wearing them.

The evidence

What was known

What's new now?

What does this mean for you?

Glossary

More information about this research study

For questions, contact: info@messagelab.org
This survey is part of a research study conducted by researchers at Dartmouth Institute and the Norwegian Institute of Public Health.
Funding: Norwegian Institute of Public Health

Centre for epidemic interventions research
Norwegian Institute of Public Health
Oslo, Norway

Dartmouth Institute
The Lisa Schwartz Foundation for Truth in Medicine
Hannover, New Hampshire, USA

What was known

Some researchers thought wearing glasses might protect against Covid infection – but the evidence was limited.

A recent systematic review* of studies examined whether eye protection – including glasses – protects against Covid infection. The review found that it might make a difference. But the studies were not randomized studies*. This means that the findings might be explained by differences in the people who happened to wear glasses rather than any effect glasses might have.

What's new now?

A new randomized study has been published.

3800 healthy adults, who normally didn't wear glasses, were chosen at random (by a computer program) either to wear glasses when around others, or not to wear them. ***Here's what happened:***

- **Benefit:** Slightly fewer people tested positive for COVID when *told to wear* glasses vs. people ***not*** *told to wear* glasses: 9.6% vs. 11.5%. That's a difference of 1.9%.
- **Harms and disadvantages:** There were no major harms linked to wearing glasses, but some possible disadvantages. One person reported a fall due to reduced vision, and about 25 people (0.2%) reported being irritated by foggy glasses, especially when also wearing a face mask.

What does this mean for you?

How well do the results apply to you?

Covid infection rates were very high in this trial (11.5% of people got Covid over two weeks, meaning there was a big surge at that time). If the Covid levels are lower in your setting, wearing glasses would make less of a difference.

What can you do?

It's reasonable to try glasses to protect against Covid: there may be some benefit and there are no obvious important harms. So...

You might choose to wear glasses if Covid levels are high like in this trial, you are a more cautious person, or you are at more risk of developing serious illness from Covid.

You might choose not to if these do not apply, if glasses are uncomfortable or interfere with mask use, or if you simply don't have access to them.

Glossary

Systematic review: A summary of studies addressing a clear question, using systematic and explicit methods to identify, select, and critically appraise relevant studies, and to collect and analyse data from them.

Randomized studies: A category of studies comparing two or more treatments in which random allocation is used to assign participants to treatment comparison groups.

Version 2: No explicit language, margin of error shown
https://sites.google.com/view/glasses-summary-5/home

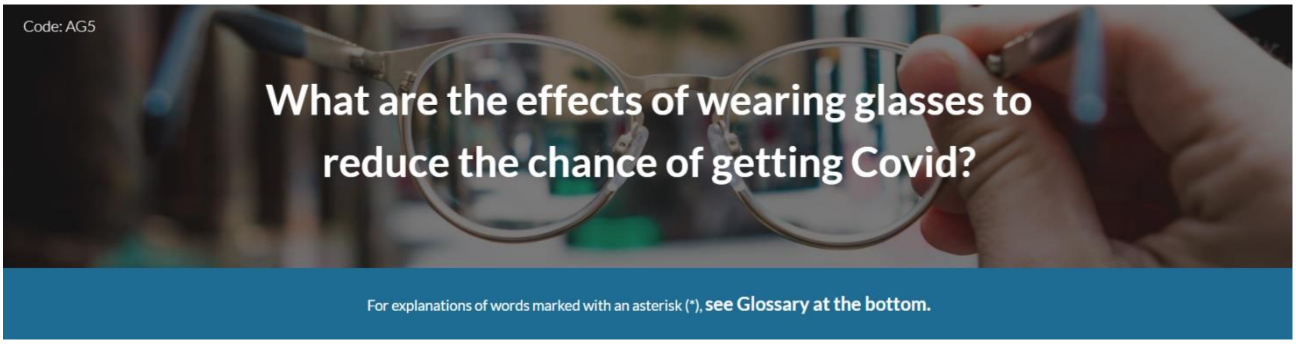

For explanations of words marked with an asterisk (*), **see Glossary at the bottom.**

Benefits

Wearing glasses may reduce your chance of getting Covid a little.

Harms and disadvantages

It is always important to consider harm when measuring effects.
Wearing glasses probably does not cause important harms, such as a serious fall due to reduced vision.

But there may be some disadvantages.
Some people are irritated by foggy glasses, and some feel silly wearing them.

The evidence

What was known

What's new now?

What does this mean for you?

Glossary

More information about this research study

For questions, contact: info@messagelab.org
This survey is part of a research study conducted by researchers at Dartmouth Institute and the Norwegian Institute of Public Health.
Funding: Norwegian Institute of Public Health

Centre for epidemic interventions research
Norwegian Institute of Public Health
Oslo, Norway

Dartmouth Institute
The Lisa Schwartz Foundation for Truth in Medicine
Hannover, New Hampshire, USA

What was known

Some researchers thought wearing glasses might protect against Covid infection – but the evidence was limited.

A recent systematic review* of studies examined whether eye protection – including glasses – protects against Covid infection. The review found that it might make a difference. But the studies were not randomized studies*. This means that the findings might be explained by differences in the people who happened to wear glasses rather than any effect glasses might have.

What's new now?

A new randomized study has been published.

3800 healthy adults, who normally didn't wear glasses, were chosen at random (by a computer program) either to wear glasses when around others, or not to wear them, for two weeks. ***Here's what happened:***

- **Benefit:** Slightly fewer people tested positive for COVID when *told to wear* glasses vs. people ***not*** *told to wear* glasses: 9.6% vs. 11.5%. That's a difference of 1.9%.

  Accounting for the play of chance (i.e., the margin of error*), glasses might *reduce* the risk of Covid by as much as 3.9% but might *increase* it by as much as 0.1%.

- **Harms and disadvantages:** There were no major harms linked to wearing glasses, but some possible disadvantages. One person reported a fall due to reduced vision, and about 25 people (0.2%) reported being irritated by foggy glasses, especially when also wearing a face mask.

What does this mean for you?

How well do the results apply to you?

Covid infection rates were very high in this trial (11.5% of people got Covid over two weeks, meaning there was a big surge at that time). If the Covid levels are lower in your setting, wearing glasses would make less of a difference.

What can you do?

It's reasonable to try glasses to protect against Covid: there may be some benefit and there are no obvious important harms. So...

You might choose to wear glasses if Covid levels are high like in this trial, you are a more cautious person, or you are at more risk of developing serious illness from Covid.

You might choose not to if these do not apply, if glasses are uncomfortable or interfere with mask use, or if you simply don't have access to them.

Glossary

Systematic review: A summary of studies addressing a clear question, using systematic and explicit methods to identify, select, and critically appraise relevant studies, and to collect and analyse data from them.

Randomized studies: A category of studies comparing two or more treatments in which random allocation is used to assign participants to treatment comparison groups.

Margin of error (confidence interval): A measure of uncertainty due to the play of chance.

Version 3: Plain language, margin of error not shown

https://sites.google.com/view/glasses-summary-4/home

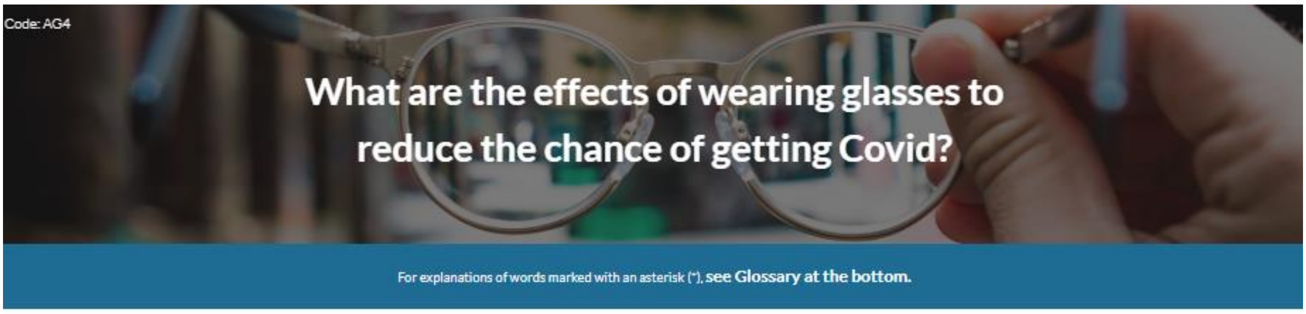

For explanations of words marked with an asterisk (*), see Glossary at the bottom.

Benefits

Wearing glasses may slightly reduce your chance of getting Covid a little – but we are not very confident about this.

Harms and disadvantages

It is always important to consider harm when measuring effects.
We are somewhat confident that wearing glasses does not cause important harms, such as a serious fall due to reduced vision.

But there may be some disadvantages.
Some people are irritated by foggy glasses, and some feel silly wearing them.

The evidence

What was known

What's new now?

Keep in mind...

What does this mean for you?

Glossary

More information about this research study

For questions, contact: info@messagelab.org
This survey is part of a research study conducted by researchers at Dartmouth Institute and the Norwegian Institute of Public Health.
Funding: Norwegian Institute of Public Health

Centre for epidemic interventions research
Norwegian Institute of Public Health
Oslo, Norway

Dartmouth Institute
The Lisa Schwartz Foundation for Truth in Medicine
Hannover, New Hampshire, USA

What was known

Some researchers thought wearing glasses might protect against Covid infection – but the evidence was limited.

A recent systematic review* of studies examined whether eye protection – including glasses – protects against Covid infection. The review found that it might make a difference. But the studies were not randomized studies*. This means that the findings might be explained by differences in the people who happened to wear glasses rather than any effect glasses might have.

What's new now?

A new randomized study has been published.

3800 healthy adults, who normally didn't wear glasses, were chosen at random (by a computer program) either to wear glasses when around others, or not to wear them. ***Here's what happened:***

- **Benefit:** Slightly fewer people tested positive for COVID when *told to wear* glasses vs. people ***not*** *told to wear* glasses: 9.6% vs. 11.5%. That's a difference of 1.9%.

- **Harms and disadvantages:** There were no major harms linked to wearing glasses, but some possible disadvantages. One person reported a fall due to reduced vision, and about 25 people (0.2%) reported being irritated by foggy glasses, especially when also wearing a face mask.

Keep in mind...

Wearing glasses may slightly reduce your chance of getting Covid – but we are not very confident about this.

One reason is because there were important study limitations*:

- ***People knew if they were told to wear glasses*** (i.e., the study was not blinded*). This may have led them to behave differently and do other things (which they were not told to do) that might affect their chance of Covid infection. In fact, we know that people in the glasses group were more likely to wear face masks - which might have reduced Covid risk. Being told to wear glasses also might have affected how often people tested themselves for Covid or reported a positive result.

- ***Some people who were meant to wear glasses didn't*** (or wore them inconsistently or for less than 2 weeks), ***and some told not to wear glasses wore them anyway***. The effect of glasses might be bigger than what the study found for people who wear glasses consistently compared to not wearing glasses at all.

What does this mean for you?

How well do the results apply to you?

Covid infection rates were very high in this trial (11.5% of people got Covid over two weeks, meaning there was a big surge at that time). If the Covid levels are lower in your setting, wearing glasses would make less of a difference.

What can you do?

It's reasonable to try glasses to protect against Covid: there may be some benefit and there are no obvious important harms. So...

You might choose to wear glasses if Covid levels are high like in this trial, you are a more cautious person, or you are at more risk of developing serious illness from Covid.

You might choose not to if these do not apply, if glasses are uncomfortable or interfere with mask use, or if you simply don't have access to them.

Glossary

Systematic review: A summary of studies addressing a clear question, using systematic and explicit methods to identify, select, and critically appraise relevant studies, and to collect and analyse data from them.

Randomized studies: A category of studies comparing two or more treatments in which random allocation is used to assign participants to treatment comparison groups.

Study limitations (risk of bias): The likelihood of there being a systematic error (bias) that distorts an effect estimate in treatment comparisons.

Blinded: In treatment comparisons, actions intended to prevent study participants (the people receiving and providing care) or the researchers (or others measuring outcomes) from knowing which participants received which treatment.

Version 4: Plain language, margin of error shown
https://sites.google.com/view/glasses-summary-3/home

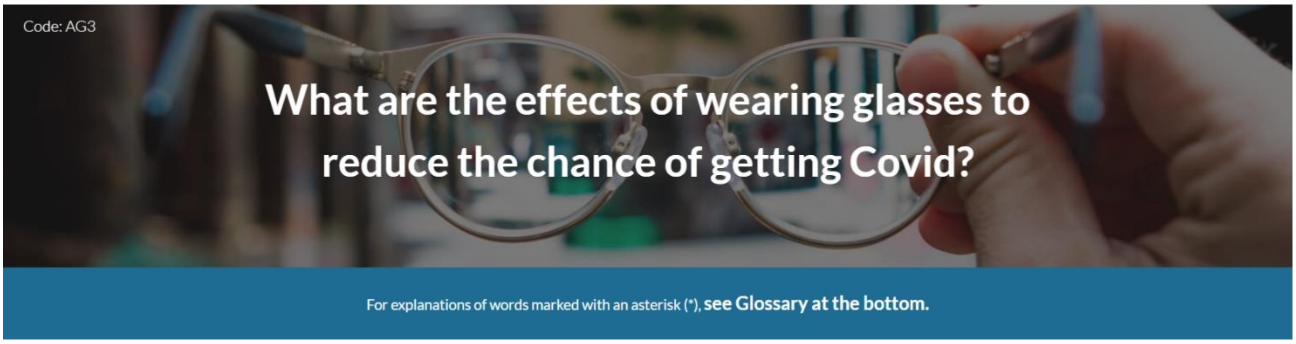

For explanations of words marked with an asterisk (*), **see Glossary at the bottom.**

Benefits

Wearing glasses may slightly reduce your chance of getting Covid a little – but we are not very confident about this.

Harms and disadvantages

It is always important to consider harm when measuring effects.
We are somewhat confident that wearing glasses does not cause important harms, such as a serious fall due to reduced vision.

But there may be some disadvantages.
Some people are irritated by foggy glasses, and some feel silly wearing them.

The evidence

What was known

What's new now?

Keep in mind...

What does this mean for you?

Glossary

More information about this research study

For questions, contact: info@messagelab.org
This survey is part of a research study conducted by researchers at Dartmouth Institute and the Norwegian Institute of Public Health.
Funding: Norwegian Institute of Public Health

Centre for epidemic interventions research
Norwegian Institute of Public Health
Oslo, Norway

Dartmouth Institute
The Lisa Schwartz Foundation for Truth in Medicine
Hannover, New Hampshire, USA

What was known

Some researchers thought wearing glasses might protect against Covid infection – but the evidence was limited.

A recent systematic review* of studies examined whether eye protection – including glasses – protects against Covid infection. The review found that it might make a difference. But the studies were not randomized studies*. This means that the findings might be explained by differences in the people who happened to wear glasses rather than any effect glasses might have.

What's new now?

A new randomized study has been published.

3800 healthy adults, who normally didn't wear glasses, were chosen at random (by a computer program) either to wear glasses when around others, or not to wear them, for two weeks. ***Here's what happened:***

- **Benefit:** Slightly fewer people tested positive for COVID when *told to wear* glasses vs. people ***not*** *told to wear* glasses: 9.6% vs. 11.5%. That's a difference of 1.9%.

  Accounting for the play of chance (i.e., the margin of error*), glasses might *reduce* the risk of Covid by as much as 3.9% but might *increase* it by as much as 0.1%.

- **Harms and disadvantages:** There were no major harms linked to wearing glasses, but some possible disadvantages. One person reported a fall due to reduced vision, and about 25 people (0.2%) reported being irritated by foggy glasses, especially when also wearing a face mask.

Keep in mind...

Wearing glasses may slightly reduce your chance of getting Covid – but we are not very confident about this.

This is because of the wide margin of error* and important study limitations*:

- ***People knew if they were told to wear glasses*** (i.e., the study was not blinded*). This may have led them to behave differently and do other things (which they were not told to do) that might affect their chance of Covid infection. In fact, we know that people in the glasses group were more likely to wear face masks - which might have reduced Covid risk. Being told to wear glasses also might have affected how often people tested themselves for Covid or reported a positive result.
- ***Some people who were meant to wear glasses didn't*** (or wore them inconsistently or for less than 2 weeks), ***and some told not to wear glasses wore them anyway***. The effect of glasses might be bigger than what the study found for people who wear glasses consistently compared to not wearing glasses at all.

What does this mean for you?

How well do the results apply to you?

Covid infection rates were very high in this trial (11.5% of people got Covid over two weeks, meaning there was a big surge at that time). If the Covid levels are lower in your setting, wearing glasses would make less of a difference.

What can you do?

It's reasonable to try glasses to protect against Covid: there may be some benefit and there are no obvious important harms. So...

You might choose to wear glasses if Covid levels are high like in this trial, you are a more cautious person, or you are at more risk of developing serious illness from Covid.

You might choose not to if these do not apply, if glasses are uncomfortable or interfere with mask use, or if you simply don't have access to them.

Glossary

Systematic review: A summary of studies addressing a clear question, using systematic and explicit methods to identify, select, and critically appraise relevant studies, and to collect and analyse data from them.

Randomized studies: A category of studies comparing two or more treatments in which random allocation is used to assign participants to treatment comparison groups.

Margin of error (confidence interval): A measure of uncertainty due to the play of chance.

Study limitations (risk of bias): The likelihood of there being a systematic error (bias) that distorts an effect estimate in treatment comparisons.

Blinded: In treatment comparisons, actions intended to prevent study participants (the people receiving and providing care) or the researchers (or others measuring outcomes) from knowing which participants received which treatment.

Version 5: GRADE language, margin of error not shown

https://sites.google.com/view/glasses-summary-2/home

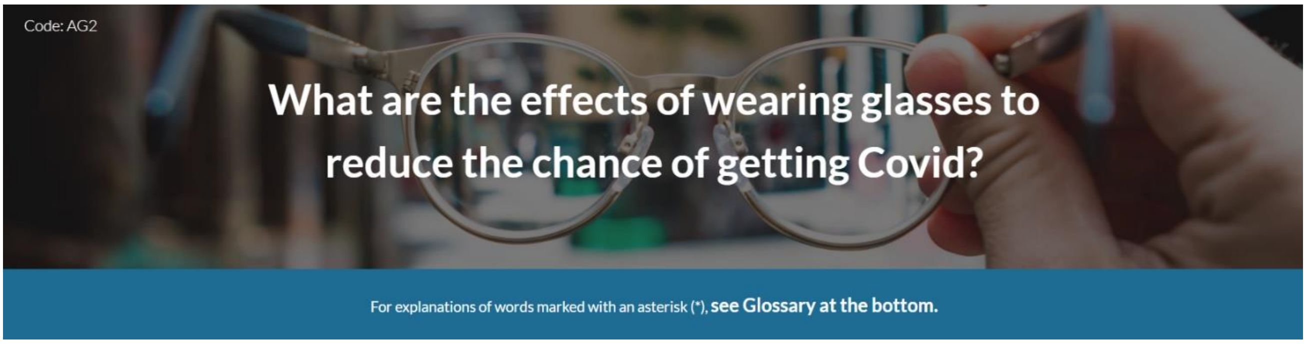

For explanations of words marked with an asterisk (*), **see Glossary at the bottom.**

Benefits

Wearing glasses may slightly reduce your chance of getting Covid.
(⨁⨁◯◯ low certainty evidence)*

Harms and disadvantages

It is always important to consider harm when measuring effects.
Wearing glasses probably does not increase your chance of any major harms, such as a serious fall due to reduced vision. (⨁⨁⨁◯ moderate certainty evidence)*

But there may be some disadvantages.
Some people are irritated by foggy glasses, and some feel silly wearing them.

The evidence

What was known

What's new now?

Keep in mind...

What does this mean for you?

Glossary

More information about this research study

For questions, contact: info@messagelab.org
This survey is part of a research study conducted by researchers at Dartmouth Institute and the Norwegian Institute of Public Health.
Funding: Norwegian Institute of Public Health

Centre for epidemic interventions research
Norwegian Institute of Public Health
Oslo, Norway

Dartmouth Institute
The Lisa Schwartz Foundation for Truth in Medicine
Hannover, New Hampshire, USA

What was known

Some researchers thought wearing glasses might protect against Covid infection – but the evidence was limited.

A recent systematic review* of studies examined whether eye protection – including glasses – protects against Covid infection. The review found that it might make a difference. But the studies were not randomized studies*. This means that the findings might be explained by differences in the people who happened to wear glasses rather than any effect glasses might have.

What's new now?

A new randomized study has been published.

3800 healthy adults, who normally didn't wear glasses, were chosen at random (by a computer program) either to wear glasses when around others, or not to wear them. ***Here's what happened:***

- **Benefit:** Slightly fewer people tested positive for COVID when *told to wear* glasses vs. people ***not*** *told to wear* glasses:9.6% vs. 11.5%. That's a difference of 1.9%.

- **Harms and disadvantages:** There were no major harms linked to wearing glasses, but some possible disadvantages. One person reported a fall due to reduced vision, and about 25 people (0.2%) reported being irritated by foggy glasses, especially when also wearing a face mask.

Keep in mind...

Wearing glasses may slightly reduce your chance of getting Covid, but this is uncertain. (⊕⊕◯◯ low certainty evidence)*

One reason is because there were important study limitations*:

- ***People knew if they were told to wear glasses*** (i.e., the study was not blinded*). This may have led them to behave differently and do other things (which they were not told to do) that might affect their chance of Covid infection. In fact, we know that people in the glasses group were more likely to wear face masks - which might have reduced Covid risk. Being told to wear glasses also might have affected how often people tested themselves for Covid or reported a positive result.

- ***Some people who were meant to wear glasses didn't*** (or wore them inconsistently or for less than 2 weeks), ***and some told not to wear glasses wore them anyway***. The effect of glasses might be bigger than what the study found for people who wear glasses consistently compared to not wearing glasses at all.

What does this mean for you?

How well do the results apply to you?

Covid infection rates were very high in this trial (11.5% of people got Covid over two weeks, meaning there was a big surge at that time). If the Covid levels are lower in your setting, wearing glasses would make less of a difference.

What can you do?

It's reasonable to try glasses to protect against Covid: there may be some benefit and there are no obvious important harms. So...

You might choose to wear glasses if Covid levels are high like in this trial, you are a more cautious person, or you are at more risk of developing serious illness from Covid.

You might choose not to if these do not apply, if glasses are uncomfortable or interfere with mask use, or if you simply don't have access to them.

Glossary

⊕◯◯◯ **Very low certainty of the evidence:** The research does not provide a reliable indication of the likely effect. The likelihood that the actual effect will be substantially different is very high.

⊕⊕◯◯ **Low certainty evidence:** The research provides some indication of the likely effect. However, the likelihood that the actual effect will be substantially different is high.

⊕⊕⊕◯ **Moderate certainty of the evidence:** The research provides a good indication of the likely effect of a treatment. The likelihood that the actual effect of the treatment will not be substantially different is moderate.

⊕⊕⊕⊕ **High certainty of the evidence:** The research provides a very good indication of the likely effect of a treatment. The likelihood that the actual effect will be substantially different from this is low.

Systematic review: A summary of studies addressing a clear question, using systematic and explicit methods to identify, select, and critically appraise relevant studies, and to collect and analyse data from them.

Randomized studies: A category of studies comparing two or more treatments in which random allocation is used to assign participants to treatment comparison groups.

Study limitations (risk of bias): The likelihood of there being a systematic error (bias) that distorts an effect estimate in treatment comparisons.

Blinded: In treatment comparisons, actions intended to prevent study participants (the people receiving and providing care) or the researchers (or others measuring outcomes) from knowing which participants received which treatment.

Version 6: GRADE language, margin of error shown

https://sites.google.com/view/glasses-summary-1/home

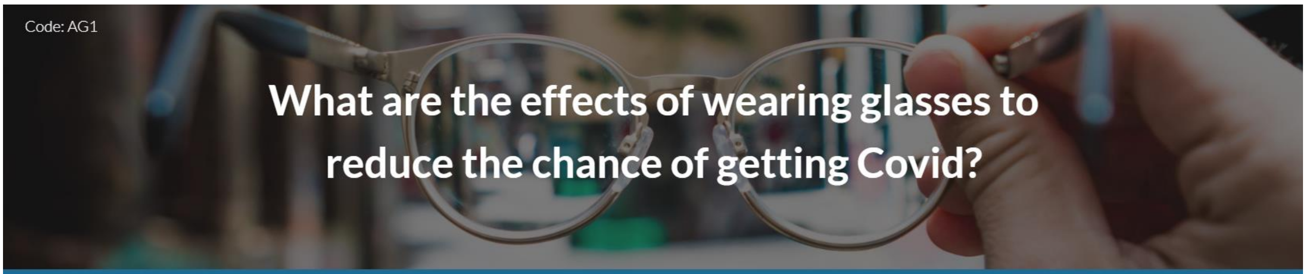

For explanations of words marked with an asterisk (*), **see Glossary at the bottom.**

Benefits

Wearing glasses may slightly reduce your chance of getting Covid.
(⨁⨁◯◯ low certainty evidence)*

Harms and disadvantages

It is always important to consider harm when measuring effects.
Wearing glasses probably does not increase your chance of any major harms, such as a serious fall due to reduced vision. (⨁⨁⨁◯ moderate certainty evidence)*

But there may be some disadvantages.
Some people are irritated by foggy glasses, and some feel silly wearing them.

The evidence

What was known

What's new now?

Keep in mind...

What does this mean for you?

Glossary

More information about this research study

For questions, contact: info@messagelab.org
This survey is part of a research study conducted by researchers at Dartmouth Institute and the Norwegian Institute of Public Health.
Funding: Norwegian Institute of Public Health

Centre for epidemic interventions research
Norwegian Institute of Public Health
Oslo, Norway

Dartmouth Institute
The Lisa Schwartz Foundation for Truth in Medicine
Hannover, New Hampshire, USA

What was known

Some researchers thought wearing glasses might protect against Covid infection – but the evidence was limited.

A recent systematic review* of studies examined whether eye protection – including glasses – protects against Covid infection. The review found that it might make a difference. But the studies were not randomized studies*. This means that the findings might be explained by differences in the people who happened to wear glasses rather than any effect glasses might have.

What's new now?

A new randomized study has been published.

3800 healthy adults, who normally didn't wear glasses, were chosen at random (by a computer program) either to wear glasses when around others, or not to wear them, for two weeks. ***Here's what happened:***

- **Benefit:** Slightly fewer people tested positive for COVID when *told to wear* glasses vs. people ***not*** *told to wear* glasses: 9.6% vs. 11.5%. That's a difference of 1.9%.

  Accounting for the play of chance (i.e., the margin of error*), glasses might *reduce* the risk of Covid by as much as 3.9% but might *increase* it by as much as 0.1%.

- **Harms and disadvantages:** There were no major harms linked to wearing glasses, but some possible disadvantages. One person reported a fall due to reduced vision, and about 25 people (0.2%) reported being irritated by foggy glasses, especially when also wearing a face mask.

Keep in mind...

Wearing glasses may slightly reduce your chance of getting Covid, but this is uncertain. (⊕⊕○○ low certainty evidence)*

This is because of the wide margin of error* and important study limitations*:

- ***People knew if they were told to wear glasses*** (i.e., the study was not blinded*). This may have led them to behave differently and do other things (which they were not told to do) that might affect their chance of Covid infection. In fact, we know that people in the glasses group were more likely to wear face masks - which might have reduced Covid risk. Being told to wear glasses also might have affected how often people tested themselves for Covid or reported a positive result.

- ***Some people who were meant to wear glasses didn't*** (or wore them inconsistently or for less than 2 weeks), ***and some told not to wear glasses wore them anyway***. The effect of glasses might be bigger than what the study found for people who wear glasses consistently compared to not wearing glasses at all.

What does this mean for you?

How well do the results apply to you?

Covid infection rates were very high in this trial (11.5% of people got Covid over two weeks, meaning there was a big surge at that time). If the Covid levels are lower in your setting, wearing glasses would make less of a difference.

What can you do?

It's reasonable to try glasses to protect against Covid: there may be some benefit and there are no obvious important harms. So...

You might choose to wear glasses if Covid levels are high like in this trial, you are a more cautious person, or you are at more risk of developing serious illness from Covid.

You might choose not to if these do not apply, if glasses are uncomfortable or interfere with mask use, or if you simply don't have access to them.

Glossary

⊕◯◯◯ **Very low certainty of the evidence:** The research does not provide a reliable indication of the likely effect. The likelihood that the actual effect will be substantially different is very high.

⊕⊕◯◯ **Low certainty evidence:** The research provides some indication of the likely effect. However, the likelihood that the actual effect will be substantially different is high.

⊕⊕⊕◯ **Moderate certainty of the evidence:** The research provides a good indication of the likely effect of a treatment. The likelihood that the actual effect of the treatment will not be substantially different is moderate.

⊕⊕⊕⊕ **High certainty of the evidence:** The research provides a very good indication of the likely effect of a treatment. The likelihood that the actual effect will be substantially different from this is low.

Systematic review: A summary of studies addressing a clear question, using systematic and explicit methods to identify, select, and critically appraise relevant studies, and to collect and analyse data from them.

Randomized studies: A category of studies comparing two or more treatments in which random allocation is used to assign participants to treatment comparison groups.

Margin of error (confidence interval): A measure of uncertainty due to the play of chance.

Study limitations (risk of bias): The likelihood of there being a systematic error (bias) that distorts an effect estimate in treatment comparisons.

Blinded: In treatment comparisons, actions intended to prevent study participants (the people receiving and providing care) or the researchers (or others measuring outcomes) from knowing which participants received which treatment.

Norwegian summaries

Version 1: No explicit language, margin of error not shown (reference / control version)

https://sites.google.com/view/oppsummering-6/home

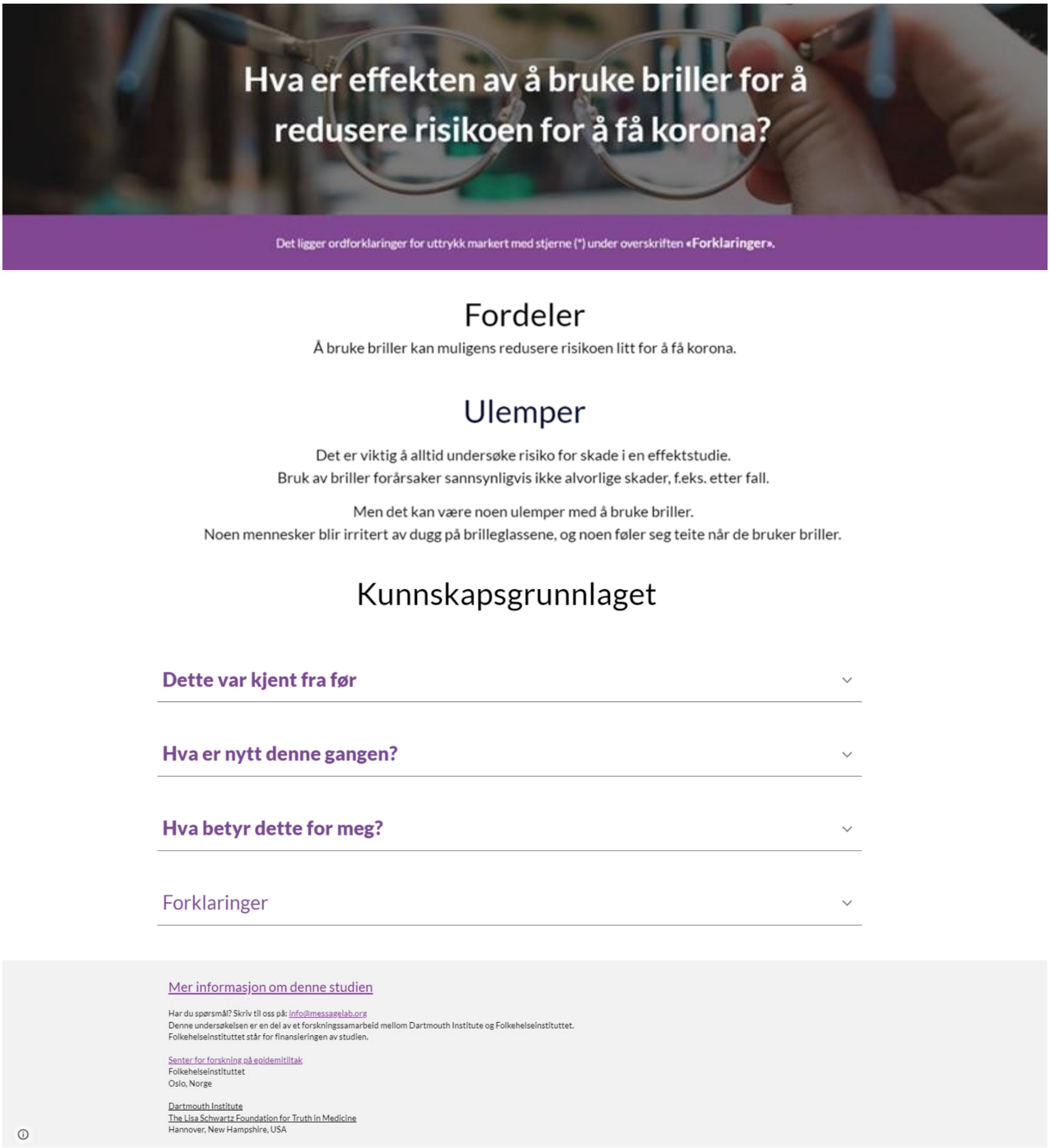

Dette var kjent fra før

Noen forskere har tenkt at det å bruke briller kan beskytte mot koronainfeksjon, men kunnskapsgrunnlaget har vært begrenset.

En kunnskapsoppsummering* av studier undersøkte om øyebeskyttelse – briller inkludert – beskytter mot koronainfeksjon. Oppsummeringen viste at det muligens kan gjøre en forskjell, men disse studiene var ikke randomiserte studier*. Dette betyr at resultatene kan forklares av forskjeller mellom dem som brukte eller ikke brukt briller, snarere enn av at det var brillene som hadde en effekt.

Hva er nytt denne gangen?

En ny randomisert studie har blitt publisert.

3800 friske voksne, som ikke brukte briller fast, ble helt tilfeldig fordelt (av et dataprogram) til enten å bruke briller når de var sammen med andre, eller å ikke bruke briller, gjennom to uker. ***Dette skjedde:***

- **Fordeler:** Litt færre av dem som fikk beskjed om *å bruke briller* testet positivt for korona, sammenlignet med dem som fikk beskjed om å ***ikke*** *bruke briller*: 9.6 % mot 11.5 %. Det er en forskjell på 1.9 %.

- **Ulemper:** Det var ingen betydelige skader forbundet med det å bruke briller. Én person fortalte om et fall på grunn av redusert syn, og rundt 25 personer (0.2 %) fortalte at de ble irritert av dugg på brillene, spesielt når de brukte munnbind.

Hva betyr dette for deg?

Er resultatene overførbare til din situasjon?

Det var mye smitte i studieperioden da 11.5 % av deltakerne i studien fikk korona i løpet av to uker. Dersom smittertrykket er lavere i dine omgivelser, vil det være mindre effekt av å bruke briller for å beskytte seg mot korona.

Hva kan du gjøre nå?

Du må gjerne prøve å bruke briller for å beskytte deg mot korona; det er muligens noen fordeler og det er ingen åpenbare ulemper. Det vil si at:

Du kan velge å bruke briller hvis koronasmittetrykket er høyt, slik som i denne studien, hvis du er en mer forsiktig person, eller hvis du er i risikogruppen for å få alvorlig sykdom av korona.

Du kan velge å ikke bruke briller, hvis brillene er ukomfortable eller hindrer munnbindbruk, eller hvis du ikke har noen tilgjengelige briller å bruke.

Forklaringer

Kunnskapsoppsummering: En oversikt over den beste, tilgjengelige kunnskapen i verden på et spesifikt tema, utført på en systematisk, eksplisitt og transparent måte.

Randomiserte studier: En type studier for sammenligning av tiltak (for eksempel medisinske behandlinger) hvor deltakerne fordeles tilfeldig i grupper, for eksempel ved loddtrekning. Deretter sammenligner man gruppene som får de forskjellige tiltakene.

Mer informasjon om denne studien

Har du spørsmål? Skriv til oss på: info@messagelab.org
Denne undersøkelsen er en del av et forskningssamarbeid mellom Dartmouth Institute og Folkehelseinstituttet.
Folkehelseinstituttet står for finansieringen av studien.

Senter for forskning på epidemitiltak
Folkehelseinstituttet
Oslo, Norge

Dartmouth Institute
The Lisa Schwartz Foundation for Truth in Medicine
Hannover, New Hampshire, USA

Version 2: No explicit language, margin of error shown
https://sites.google.com/view/oppsummering-5/home

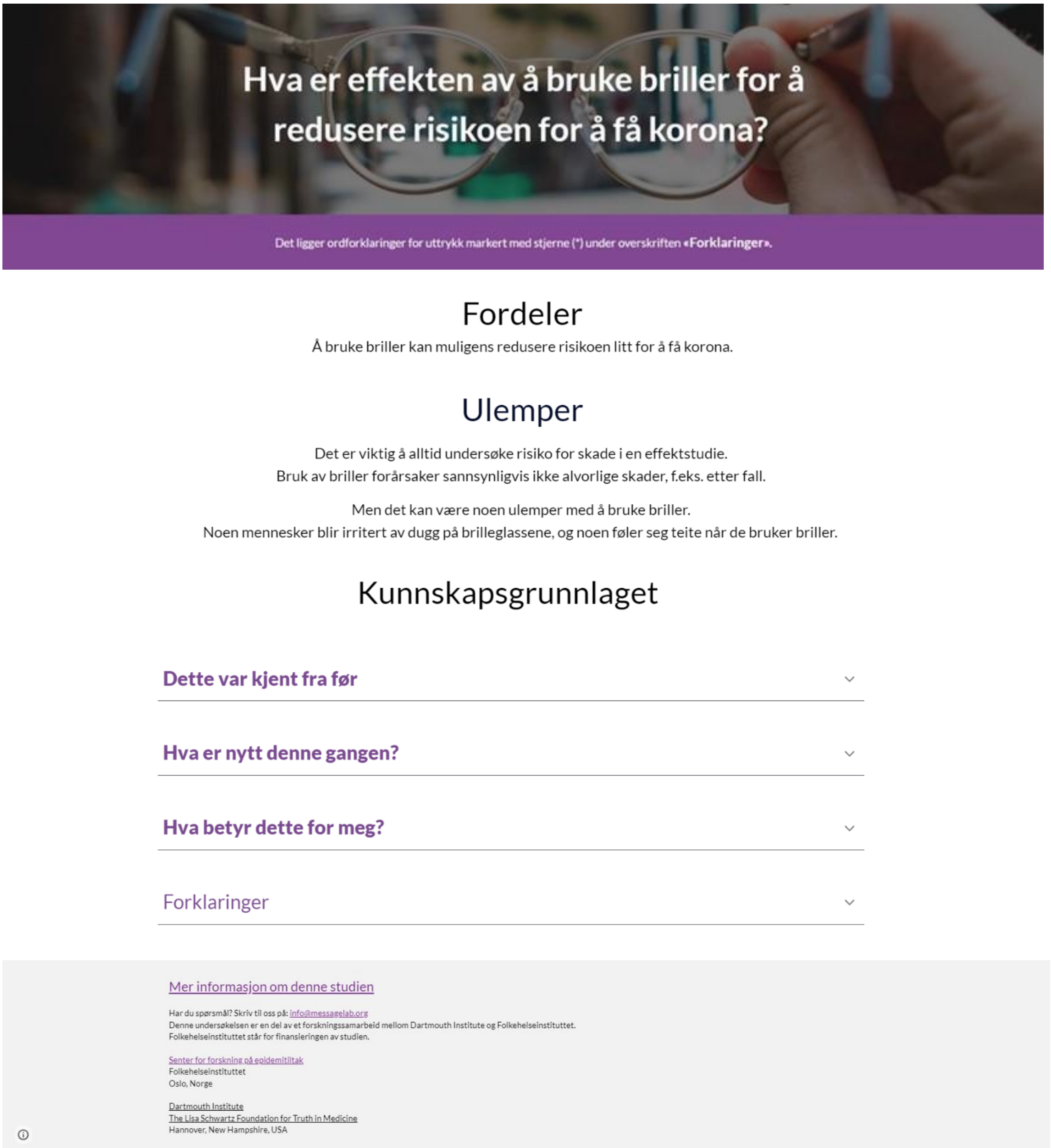

Hva er effekten av å bruke briller for å redusere risikoen for å få korona?

Det ligger ordforklaringer for uttrykk markert med stjerne (*) under overskriften «Forklaringer».

Fordeler

Å bruke briller kan muligens redusere risikoen litt for å få korona.

Ulemper

Det er viktig å alltid undersøke risiko for skade i en effektstudie.
Bruk av briller forårsaker sannsynligvis ikke alvorlige skader, f.eks. etter fall.

Men det kan være noen ulemper med å bruke briller.
Noen mennesker blir irritert av dugg på brilleglassene, og noen føler seg teite når de bruker briller.

Kunnskapsgrunnlaget

Dette var kjent fra før

Hva er nytt denne gangen?

Hva betyr dette for meg?

Forklaringer

Mer informasjon om denne studien

Har du spørsmål? Skriv til oss på: info@messagelab.org
Denne undersøkelsen er en del av et forskningssamarbeid mellom Dartmouth Institute og Folkehelseinstituttet.
Folkehelseinstituttet står for finansieringen av studien.

Senter for forskning på epidemitiltak
Folkehelseinstituttet
Oslo, Norge

Dartmouth Institute
The Lisa Schwartz Foundation for Truth in Medicine
Hannover, New Hampshire, USA

Dette var kjent fra før

Noen forskere har tenkt at det å bruke briller kan beskytte mot koronainfeksjon, men kunnskapsgrunnlaget har vært begrenset.

En kunnskapsoppsummering* av studier undersøkte om øyebeskyttelse – briller inkludert – beskytter mot koronainfeksjon. Oppsummeringen viste at det muligens kan gjøre en forskjell, men disse studiene var ikke randomiserte studier*. Dette betyr at resultatene kan forklares av forskjeller mellom dem som brukte eller ikke brukt briller, snarere enn av at det var brillene som hadde en effekt.

Hva er nytt denne gangen?

En ny randomisert studie har blitt publisert.

3800 friske voksne, som ikke brukte briller fast, ble helt tilfeldig fordelt (av et dataprogram) til enten å bruke briller når de var sammen med andre, eller å ikke bruke briller, gjennom to uker. ***Dette skjedde:***

- **Fordeler:** Litt færre av dem som fikk beskjed om *å bruke briller* testet positivt for korona, sammenlignet med dem som fikk beskjed om å ***ikke*** *bruke briller*: 9.6 % mot 11.5 %. Det er en forskjell på 1.9 %.

  Med tilfeldige feil (dvs. feilmarginen*) tatt i betraktning, kan briller redusere risikoen for å få korona med så mye som 3.9 %, men briller kan også øke risikoen for å få korona med opp til 0.1 %.

- **Ulemper:** Det var ingen betydelige skader forbundet med det å bruke briller. Én person fortalte om et fall på grunn av redusert syn, og rundt 25 personer (0.2 %) fortalte at de ble irritert av dugg på brillene, spesielt når de brukte munnbind.

Hva betyr dette for deg?

Er resultatene overførbare til din situasjon?

Det var mye smitte i studieperioden da 11.5 % av deltakerne i studien fikk korona i løpet av to uker. Dersom smittertrykket er lavere i dine omgivelser, vil det være mindre effekt av å bruke briller for å beskytte seg mot korona.

Hva kan du gjøre nå?

Du må gjerne prøve å bruke briller for å beskytte deg mot korona; det er muligens noen fordeler og det er ingen åpenbare ulemper. Det vil si at:

Du kan velge å bruke briller hvis koronasmittetrykket er høyt, slik som i denne studien, hvis du er en mer forsiktig person, eller hvis du er i risikogruppen for å få alvorlig sykdom av korona.

Du kan velge å ikke bruke briller, hvis brillene er ukomfortable eller hindrer munnbindbruk, eller hvis du ikke har noen tilgjengelige briller å bruke.

Forklaringer

Kunnskapsoppsummering: En oversikt over den beste, tilgjengelige kunnskapen i verden på et spesifikt tema, utført på en systematisk, eksplisitt og transparent måte.

Randomiserte studier: En type studier for sammenligning av tiltak (for eksempel medisinske behandlinger) hvor deltakerne fordeles tilfeldig i grupper, for eksempel ved loddtrekning. Deretter sammenligner man gruppene som får de forskjellige tiltakene.

Feilmargin (konfidensintervall): Et mål på usikkerhet rundt den beregnede effekten, som følge av tilfeldige feil.

Mer informasjon om denne studien

Har du spørsmål? Skriv til oss på: info@messagelab.org
Denne undersøkelsen er en del av et forskningssamarbeid mellom Dartmouth Institute og Folkehelseinstituttet.
Folkehelseinstituttet står for finansieringen av studien.

Senter for forskning på epidemitiltak
Folkehelseinstituttet
Oslo, Norge

Dartmouth Institute
The Lisa Schwartz Foundation for Truth in Medicine
Hannover, New Hampshire, USA

Version 3: Plain language, margin of error not shown

https://sites.google.com/view/oppsummering-4/home

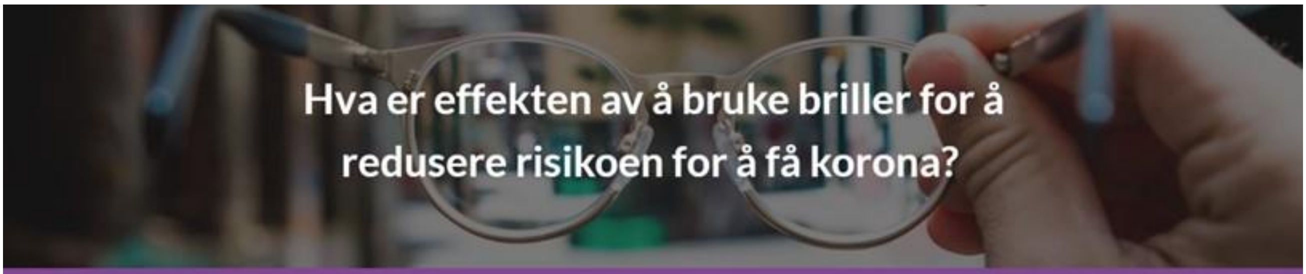

Det ligger ordforklaringer for uttrykk markert med stjerne (*) under overskriften «**Forklaringer**».

Fordeler

Å bruke briller kan muligens redusere risikoen litt for å få korona – men vi er ikke veldig sikre på dette.

Ulemper

Det er viktig å alltid undersøke risiko for skade i en effektstudie.
Vi er noenlunde sikre på at bruk av briller sannsynligvis ikke øker risiko for alvorlige skader, f.eks. etter fall.

Men det kan være noen ulemper med å bruke briller.
Noen mennesker blir irritert av dugg på brilleglassene, og noen føler seg teite når de bruker briller.

Kunnskapsgrunnlaget

Dette var kjent fra før

Hva er nytt denne gangen?

Husk!

Hva betyr dette for deg?

Forklaringer

Mer informasjon om denne studien

Har du spørsmål? Skriv til oss på: info@messagelab.org
Denne undersøkelsen er en del av et forskningssamarbeid mellom Dartmouth Institute og Folkehelseinstituttet.
Folkehelseinstituttet står for finansieringen av studien.

Senter for forskning på epidemitiltak
Folkehelseinstituttet
Oslo, Norge

Dartmouth Institute
The Lisa Schwartz Foundation for Truth in Medicine
Hannover, New Hampshire, USA

Dette var kjent fra før

Noen forskere har tenkt at det å bruke briller kan beskytte mot koronainfeksjon, men kunnskapsgrunnlaget har vært begrenset.

En kunnskapsoppsummering* av studier undersøkte om øyebeskyttelse – briller inkludert – beskytter mot koronainfeksjon. Oppsummeringen viste at det muligens kan gjøre en forskjell, men disse studiene var ikke randomiserte studier*. Dette betyr at resultatene kan forklares av forskjeller mellom dem som brukte eller ikke brukt briller, snarere enn av at det var brillene som hadde en effekt.

Hva er nytt denne gangen?

En ny randomisert studie har blitt publisert.

3800 friske voksne, som ikke brukte briller fast, ble helt tilfeldig fordelt (av et dataprogram) til enten å bruke briller når de var sammen med andre, eller å ikke bruke briller, gjennom to uker. ***Dette skjedde:***

- **Fordeler:** Litt færre av dem som fikk beskjed om *å bruke briller* testet positivt for korona, sammenlignet med dem som fikk beskjed om å ***ikke*** *bruke briller*: 9.6 % mot 11.5 %. Det er en forskjell på 1.9 %.

- **Ulemper:** Det var ingen betydelige skader forbundet med det å bruke briller. Én person fortalte om et fall på grunn av redusert syn, og rundt 25 personer (0.2 %) fortalte at de ble irritert av dugg på brillene, spesielt når de brukte munnbind.

Husk!

Bruk av briller kan reduserer risikoen for korona litt – men vi er ikke veldig sikre på dette.

Viktige begrensninger ved studien* kan være én av grunnene til usikkerheten:

- ***Deltakere i studien visste at de hadde blitt bedt om å bruke briller (dvs. at studien ikke var blindet*).*** Dette kan ha ført til at de oppførte seg annerledes og gjorde andre ting (som de faktisk ble rådet til ikke å gjøre) som kan ha påvirket deres risiko for å få korona. Faktisk så vet vi at flere i brillegruppen brukte munnbind – noe som kan ha redusert risikoen for å få korona. Det å bli bedt om å bruke briller kan også ha hatt noe å si for hvor ofte personene koronatestet seg selv eller meldte inn et positivt testsvar.

- ***Noen personer som ble bedt om å bruke briller brukte dem ikke*** (eller brukte dem av og til, eller mindre enn i to uker), ***og noen som ble bedt om å ikke bruke briller brukte dem allikevel.*** Det betyr at effekten av brillebruk kan være større enn hva studien fant for dem som alltid bruker briller, sammenliknet med dem som ikke bruker briller i det hele tatt.

Hva betyr dette for deg?

Er resultatene overførbare til din situasjon?

Det var mye smitte i studieperioden da 11.5 % av deltakerne i studien fikk korona i løpet av to uker. Dersom smittertrykket er lavere i dine omgivelser, vil det være mindre effekt av å bruke briller for å beskytte seg mot korona.

Hva kan du gjøre nå?

Du må gjerne prøve å bruke briller for å beskytte deg mot korona; det er muligens noen fordeler og det er ingen åpenbare ulemper. Det vil si at:

Du kan velge å bruke briller hvis koronasmittetrykket er høyt, slik som i denne studien, hvis du er en mer forsiktig person, eller hvis du er i risikogruppen for å få alvorlig sykdom av korona.

Du kan velge å ikke bruke briller, hvis brillene er ukomfortable eller hindrer munnbindbruk, eller hvis du ikke har noen tilgjengelige briller å bruke.

Forklaringer

Kunnskapsoppsummering: En oversikt over den beste, tilgjengelige kunnskapen i verden på et spesifikt tema, utført på en systematisk, eksplisitt og transparent måte.

Randomiserte studier: En type studier for sammenligning av tiltak (for eksempel medisinske behandlinger) hvor deltakerne fordeles tilfeldig i grupper, for eksempel ved loddtrekning. Deretter sammenligner man gruppene som får de forskjellige tiltakene.

Begrensninger ved studien (risiko for bias): Risiko for systematiske feil (skjevheter) i resultater pga. svakheter i en studie.

Blindet: Å sørge for at deltakere, behandlere og forskere ikke vet hvilken gruppe deltakerne tilhører.

Version 4: Plain language, margin of error shown

https://sites.google.com/view/oppsummering-3/home

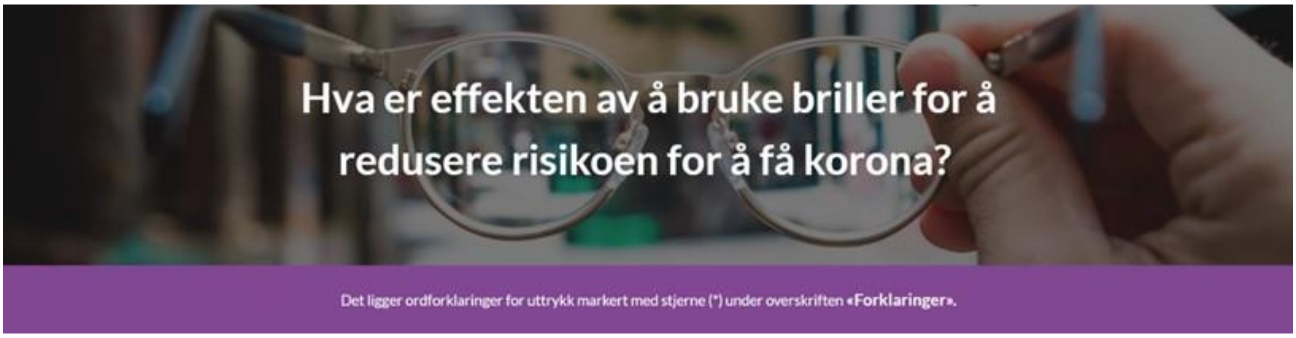

Fordeler

Å bruke briller kan muligens redusere risikoen litt for å få korona – men vi er ikke veldig sikre på dette.

Ulemper

Det er viktig å alltid undersøke risiko for skade i en effektstudie.
Vi er noenlunde sikre på at bruk av briller sannsynligvis ikke øker risiko for alvorlige skader, f.eks. etter fall.

Men det kan være noen ulemper med å bruke briller.
Noen mennesker blir irritert av dugg på brilleglassene, og noen føler seg teite når de bruker briller.

Kunnskapsgrunnlaget

Dette var kjent fra før

Hva er nytt denne gangen?

Husk!

Hva betyr dette for deg?

Forklaringer

More information about this research study

For questions, contact: info@messagelab.org
This survey is part of a research study conducted by researchers at Dartmouth Institute and the Norwegian Institute of Public Health.
Funding: Norwegian Institute of Public Health

Centre for epidemic interventions research
Norwegian Institute of Public Health
Oslo, Norway

Dartmouth Institute
The Lisa Schwartz Foundation for Truth in Medicine
Hannover, New Hampshire, USA

Dette var kjent fra før

Noen forskere har tenkt at det å bruke briller kan beskytte mot koronainfeksjon, men kunnskapsgrunnlaget har vært begrenset.

En kunnskapsoppsummering* av studier undersøkte om øyebeskyttelse – briller inkludert – beskytter mot koronainfeksjon. Oppsummeringen viste at det muligens kan gjøre en forskjell, men disse studiene var ikke randomiserte studier*. Dette betyr at resultatene kan forklares av forskjeller mellom dem som brukte eller ikke brukt briller, snarere enn av at det var brillene som hadde en effekt.

Hva er nytt denne gangen?

En ny randomisert studie har blitt publisert.

3800 friske voksne, som ikke brukte briller fast, ble helt tilfeldig fordelt (av et dataprogram) til enten å bruke briller når de var sammen med andre, eller å ikke bruke briller, gjennom to uker. ***Dette skjedde:***

- **Fordeler:** Litt færre av dem som fikk beskjed om *å bruke briller* testet positivt for korona, sammenlignet med dem som fikk beskjed om å ***ikke*** *bruke briller*: 9.6 % mot 11.5 %. Det er en forskjell på 1.9 %.

  Med tilfeldige feil (dvs. feilmarginen*) tatt i betraktning, kan briller redusere risikoen for å få korona med så mye som 3.9 %, men briller kan også øke risikoen for å få korona med opp til 0.1 %.

- **Ulemper:** Det var ingen betydelige skader forbundet med det å bruke briller. Én person fortalte om et fall på grunn av redusert syn, og rundt 25 personer (0.2 %) fortalte at de ble irritert av dugg på brillene, spesielt når de brukte munnbind.

Husk!

Bruk av briller kan reduserer risikoen for korona litt – men vi er ikke veldig sikre på dette.

Den store feilmarginen* og viktige begrensninger ved studien* er grunnene til usikkerheten:

- ***Deltakere i studien visste at de hadde blitt bedt om å bruke briller (dvs. at studien ikke var blindet*).*** Dette kan ha ført til at de oppførte seg annerledes og gjorde andre ting (som de faktisk ble rådet til ikke å gjøre) som kan ha påvirket deres risiko for å få korona. Faktisk så vet vi at flere i brillegruppen brukte munnbind – noe som kan ha redusert risikoen for å få korona. Det å bli bedt om å bruke briller kan også ha hatt noe å si for hvor ofte personene koronatestet seg selv eller meldte inn et positivt testsvar.

- ***Noen personer som ble bedt om å bruke briller brukte dem ikke*** (eller brukte dem av og til, eller mindre enn i to uker), ***og noen som ble bedt om å ikke bruke briller brukte dem allikevel.*** Det betyr at effekten av brillebruk kan være større enn hva studien fant for dem som alltid bruker briller, sammenliknet med dem som ikke bruker briller i det hele tatt.

Hva betyr dette for deg?

Er resultatene overførbare til din situasjon?

Det var mye smitte i studieperioden da 11.5 % av deltakerne i studien fikk korona i løpet av to uker. Dersom smittertrykket er lavere i dine omgivelser, vil det være mindre effekt av å bruke briller for å beskytte seg mot korona.

Hva kan du gjøre nå?

Du må gjerne prøve å bruke briller for å beskytte deg mot korona; det er muligens noen fordeler og det er ingen åpenbare ulemper. Det vil si at:

Du kan velge å bruke briller hvis koronasmittetrykket er høyt, slik som i denne studien, hvis du er en mer forsiktig person, eller hvis du er i risikogruppen for å få alvorlig sykdom av korona.

Du kan velge å ikke bruke briller, hvis brillene er ukomfortable eller hindrer munnbindbruk, eller hvis du ikke har noen tilgjengelige briller å bruke.

Forklaringer

Kunnskapsoppsummering: En oversikt over den beste, tilgjengelige kunnskapen i verden på et spesifikt tema, utført på en systematisk, eksplisitt og transparent måte.

Randomiserte studier: En type studier for sammenligning av tiltak (for eksempel medisinske behandlinger) hvor deltakerne fordeles tilfeldig i grupper, for eksempel ved loddtrekning. Deretter sammenligner man gruppene som får de forskjellige tiltakene.

Feilmargin (konfidensintervall): Et mål på usikkerhet rundt den beregnede effekten, som følge av tilfeldige feil.

Begrensninger ved studien (risiko for bias): Risiko for systematiske feil (skjevheter) i resultater pga. svakheter i en studie.

Blindet: Å sørge for at deltakere, behandlere og forskere ikke vet hvilken gruppe deltakerne tilhører.

Version 5: GRADE language, margin of error not shown

https://sites.google.com/view/oppsummering-2/home

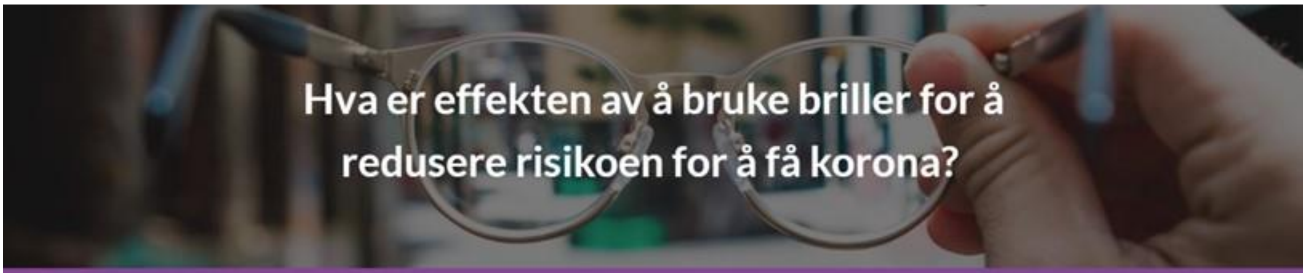

Det ligger ordforklaringer for uttrykk markert med stjerne (*) under overskriften «**Forklaringer**».

Fordeler

Å bruke briller kan muligens redusere risikoen litt for å få korona.
(⊕⊕◯◯ lav tillit til kunnskapsgrunnlaget*.)

Ulemper

Det er viktig å alltid undersøke risiko for skade i en effektstudie.
Bruk av briller øker sannsynligvis ikke risikoen for alvorlige skader, f.eks. etter fall.
(⊕⊕⊕◯ moderat tillit til kunnskapsgrunnlaget*.)

Men det kan være noen ulemper med å bruke briller.
Noen mennesker blir irritert av dugg på brilleglassene, og noen føler seg teite når de bruker briller.

Kunnskapsgrunnlaget

Dette var kjent fra før

Hva er nytt denne gangen?

Husk!

Hva betyr dette for deg?

Forklaringer

Mer informasjon om denne studien

Har du spørsmål? Skriv til oss på: info@messagelab.org
Denne undersøkelsen er en del av et forskningssamarbeid mellom Dartmouth Institute og Folkehelseinstituttet.
Folkehelseinstituttet står for finansieringen av studien.

Senter for forskning på epidemitiltak
Folkehelseinstituttet
Oslo, Norge

Dartmouth Institute
The Lisa Schwartz Foundation for Truth in Medicine
Hannover, New Hampshire, USA

Dette var kjent fra før

Noen forskere har tenkt at det å bruke briller kan beskytte mot koronainfeksjon, men kunnskapsgrunnlaget har vært begrenset.

En kunnskapsoppsummering* av studier undersøkte om øyebeskyttelse – briller inkludert – beskytter mot koronainfeksjon. Oppsummeringen viste at det muligens kan gjøre en forskjell, men disse studiene var ikke randomiserte studier*. Dette betyr at resultatene kan forklares av forskjeller mellom dem som brukte eller ikke brukt briller, snarere enn av at det var brillene som hadde en effekt.

Hva er nytt denne gangen?

En ny randomisert studie har blitt publisert.

3800 friske voksne, som ikke brukte briller fast, ble helt tilfeldig fordelt (av et dataprogram) til enten å bruke briller når de var sammen med andre, eller å ikke bruke briller, gjennom to uker. ***Dette skjedde:***

- **Fordeler:** Litt færre av dem som fikk beskjed om *å bruke briller* testet positivt for korona, sammenlignet med dem som fikk beskjed om å ***ikke*** *bruke briller*: 9.6 % mot 11.5 %. Det er en forskjell på 1.9 %.

- **Ulemper:** Det var ingen betydelige skader forbundet med det å bruke briller. Én person fortalte om et fall på grunn av redusert syn, og rundt 25 personer (0.2 %) fortalte at de ble irritert av dugg på brillene, spesielt når de brukte munnbind.

Husk!

Bruk av briller kan reduserer risikoen for korona litt – men dette er usikkert.

(⊕⊕◯◯ lav tillit til kunnskapsgrunnlaget*.)

Viktige begrensninger ved studien* kan være én av grunnene til usikkerheten:

- ***Deltakere i studien visste at de hadde blitt bedt om å bruke briller (dvs. at studien ikke var blindet*).*** Dette kan ha ført til at de oppførte seg annerledes og gjorde andre ting (som de faktisk ble rådet til ikke å gjøre) som kan ha påvirket deres risiko for å få korona. Faktisk så vet vi at flere i brillegruppen brukte munnbind – noe som kan ha redusert risikoen for å få korona. Det å bli bedt om å bruke briller kan også ha hatt noe å si for hvor ofte personene koronatestet seg selv eller meldte inn et positivt testsvar.

- ***Noen personer som ble bedt om å bruke briller brukte dem ikke*** (eller brukte dem av og til, eller mindre enn i to uker), ***og noen som ble bedt om å ikke bruke briller brukte dem allikevel.*** Det betyr at effekten av brillebruk kan være større enn hva studien fant for dem som alltid bruker briller, sammenliknet med dem som ikke bruker briller i det hele tatt.

Hva betyr dette for deg?

Er resultatene overførbare til din situasjon?

Det var mye smitte i studieperioden da 11.5 % av deltakerne i studien fikk korona i løpet av to uker. Dersom smittertrykket er lavere i dine omgivelser, vil det være mindre effekt av å bruke briller for å beskytte seg mot korona.

Hva kan du gjøre nå?

Du må gjerne prøve å bruke briller for å beskytte deg mot korona; det er muligens noen fordeler og det er ingen åpenbare ulemper. Det vil si at:

Du kan velge å bruke briller hvis koronasmittetrykket er høyt, slik som i denne studien, hvis du er en mer forsiktig person, eller hvis du er i risikogruppen for å få alvorlig sykdom av korona.

Du kan velge å ikke bruke briller, hvis brillene er ukomfortable eller hindrer munnbindbruk, eller hvis du ikke har noen tilgjengelige briller å bruke.

Forklaringer

⊕○○○ **Svært lav tillit til kunnskapsgrunnlaget:** Forskningen gir ikke en pålitelig pekepinn på den sanne effekten. Det er høy sannsynlighet for at den sanne effekten er vesentlig forskjellig fra den som er oppgitt.

⊕⊕○○ **Lav tillit til kunnskapsgrunnlaget:** Forskningen gir en viss pekepinn på den sanne effekten. Allikevel er det høy sannsynlighet for at den sanne effekten er vesentlig forskjellig fra den som er oppgitt.

⊕⊕⊕○ **Moderat tillit til kunnskapsgrunnlaget:** Forskningen gir en god pekepinn på den sanne effekten. Det er moderat sannsynlighet for at den sanne effekten er vesentlig forskjellig fra den som er oppgitt.

⊕⊕⊕⊕ **Høy tillit til kunnskapsgrunnlaget:** Forskningen gir en veldig god pekepinn på den sanne effekten. Det er lav sannsynlighet for at den sanne effekten er vesentlig forskjellig fra den som er oppgitt.

Kunnskapsoppsummering: En oversikt over den beste, tilgjengelige kunnskapen i verden på et spesifikt tema, utført på en systematisk, eksplisitt og transparent måte.

Randomiserte studier: En type studier for sammenligning av tiltak (for eksempel medisinske behandlinger) hvor deltakerne fordeles tilfeldig i grupper, for eksempel ved loddtrekning. Deretter sammenligner man gruppene som får de forskjellige tiltakene.

Begrensninger ved studien (risiko for bias): Risiko for systematiske feil (skjevheter) i resultater pga. svakheter i en studie.

Blindet: Å sørge for at deltakere, behandlere og forskere ikke vet hvilken gruppe deltakerne tilhører.

Version 6: GRADE language, margin of error shown

https://sites.google.com/view/oppsummering-1/home

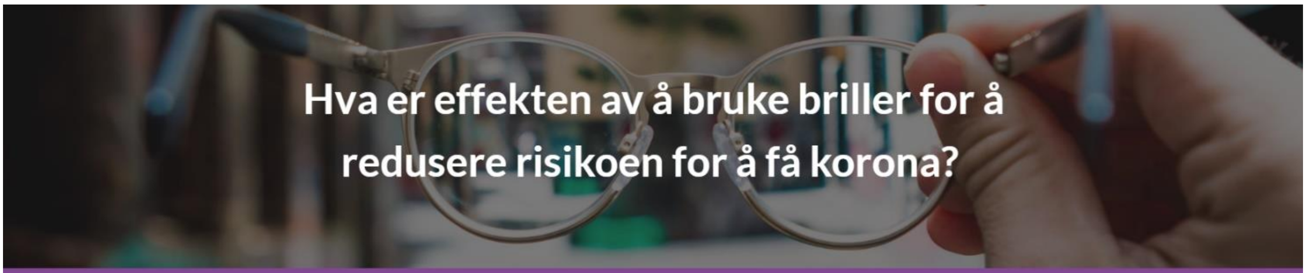

Det ligger ordforklaringer for uttrykk markert med stjerne (*) under overskriften **«Forklaringer».**

Fordeler

Å bruke briller kan muligens redusere risikoen litt for å få korona.
(⊕⊕◯◯ lav tillit til kunnskapsgrunnlaget*.)

Ulemper

Det er viktig å alltid undersøke risiko for skade i en effektstudie.
Bruk av briller øker sannsynligvis ikke risikoen for alvorlige skader, f.eks. etter fall.
(⊕⊕⊕◯ moderat tillit til kunnskapsgrunnlaget*.)

Men det kan være noen ulemper med å bruke briller.
Noen mennesker blir irritert av dugg på brilleglassene, og noen føler seg teite når de bruker briller.

Kunnskapsgrunnlaget

Dette var kjent fra før

Hva er nytt denne gangen?

Husk!

Hva betyr dette for deg?

Forklaringer

Mer informasjon om denne studien

Har du spørsmål? Skriv til oss på: info@messagelab.org
Denne undersøkelsen er en del av et forskningssamarbeid mellom Dartmouth Institute og Folkehelseinstituttet.
Folkehelseinstituttet står for finansieringen av studien.

Senter for forskning på epidemitiltak
Folkehelseinstituttet
Oslo, Norge

Dartmouth Institute
The Lisa Schwartz Foundation for Truth in Medicine
Hannover, New Hampshire, USA

Dette var kjent fra før

Noen forskere har tenkt at det å bruke briller kan beskytte mot koronainfeksjon, men kunnskapsgrunnlaget har vært begrenset.

En kunnskapsoppsummering* av studier undersøkte om øyebeskyttelse – briller inkludert – beskytter mot koronainfeksjon. Oppsummeringen viste at det muligens kan gjøre en forskjell, men disse studiene var ikke randomiserte studier*. Dette betyr at resultatene kan forklares av forskjeller mellom dem som brukte eller ikke brukt briller, snarere enn av at det var brillene som hadde en effekt.

Hva er nytt denne gangen?

En ny randomisert studie har blitt publisert.

3800 friske voksne, som ikke brukte briller fast, ble helt tilfeldig fordelt (av et dataprogram) til enten å bruke briller når de var sammen med andre, eller å ikke bruke briller, gjennom to uker. ***Dette skjedde:***

- **Fordeler:** Litt færre av dem som fikk beskjed om *å bruke briller* testet positivt for korona, sammenlignet med dem som fikk beskjed om å ***ikke*** *bruke briller*: 9.6 % mot 11.5 %. Det er en forskjell på 1.9 %.

  Med tilfeldige feil (dvs. feilmarginen*) tatt i betraktning, kan briller redusere risikoen for å få korona med så mye som 3.9 %, men briller kan også øke risikoen for å få korona med opp til 0.1 %.

- **Ulemper:** Det var ingen betydelige skader forbundet med det å bruke briller. Én person fortalte om et fall på grunn av redusert syn, og rundt 25 personer (0.2 %) fortalte at de ble irritert av dugg på brillene, spesielt når de brukte munnbind.

Husk!

Bruk av briller kan reduserer risikoen for korona litt – men dette er usikkert.

(⨁⨁◯◯ lav tillit til kunnskapsgrunnlaget*.)

Den store feilmarginen* og viktige begrensninger ved studien* er grunnene til usikkerheten:

- ***Deltakere i studien visste at de hadde blitt bedt om å bruke briller (dvs. at studien ikke var blindet*).*** Dette kan ha ført til at de oppførte seg annerledes og gjorde andre ting (som de faktisk ble rådet til ikke å gjøre) som kan ha påvirket deres risiko for å få korona. Faktisk så vet vi at flere i brillegruppen brukte munnbind – noe som kan ha redusert risikoen for å få korona. Det å bli bedt om å bruke briller kan også ha hatt noe å si for hvor ofte personene koronatestet seg selv eller meldte inn et positivt testsvar.

- ***Noen personer som ble bedt om å bruke briller brukte dem ikke*** (eller brukte dem av og til, eller mindre enn i to uker), ***og noen som ble bedt om å ikke bruke briller brukte dem allikevel.*** Det betyr at effekten av brillebruk kan være større enn hva studien fant for dem som alltid bruker briller, sammenliknet med dem som ikke bruker briller i det hele tatt.

Hva betyr dette for deg?

Er resultatene overførbare til din situasjon?

Det var mye smitte i studieperioden da 11.5 % av deltakerne i studien fikk korona i løpet av to uker. Dersom smittertrykket er lavere i dine omgivelser, vil det være mindre effekt av å bruke briller for å beskytte seg mot korona.

Hva kan du gjøre nå?

Du må gjerne prøve å bruke briller for å beskytte deg mot korona; det er muligens noen fordeler og det er ingen åpenbare ulemper. Det vil si at:

Du kan velge å bruke briller hvis koronasmittetrykket er høyt, slik som i denne studien, hvis du er en mer forsiktig person, eller hvis du er i risikogruppen for å få alvorlig sykdom av korona.

Du kan velge å ikke bruke briller, hvis brillene er ukomfortable eller hindrer munnbindbruk, eller hvis du ikke har noen tilgjengelige briller å bruke.

Forklaringer

⊕◯◯◯ **Svært lav tillit til kunnskapsgrunnlaget:** Forskningen gir ikke en pålitelig pekepinn på den sanne effekten. Det er høy sannsynlighet for at den sanne effekten er vesentlig forskjellig fra den som er oppgitt.

⊕⊕◯◯ **Lav tillit til kunnskapsgrunnlaget:** Forskningen gir en viss pekepinn på den sanne effekten. Allikevel er det høy sannsynlighet for at den sanne effekten er vesentlig forskjellig fra den som er oppgitt.

⊕⊕⊕◯ **Moderat tillit til kunnskapsgrunnlaget:** Forskningen gir en god pekepinn på den sanne effekten. Det er moderat sannsynlighet for at den sanne effekten er vesentlig forskjellig fra den som er oppgitt.

⊕⊕⊕⊕ **Høy tillit til kunnskapsgrunnlaget:** Forskningen gir en veldig god pekepinn på den sanne effekten. Det er lav sannsynlighet for at den sanne effekten er vesentlig forskjellig fra den som er oppgitt.

Kunnskapsoppsummering: En oversikt over den beste, tilgjengelige kunnskapen i verden på et spesifikt tema, utført på en systematisk, eksplisitt og transparent måte.

Randomiserte studier: En type studier for sammenligning av tiltak (for eksempel medisinske behandlinger) hvor deltakerne fordeles tilfeldig i grupper, for eksempel ved loddtrekning. Deretter sammenligner man gruppene som får de forskjellige tiltakene.

Feilmargin (konfidensintervall): Et mål på usikkerhet rundt den beregnede effekten, som følge av tilfeldige feil.

Begrensninger ved studien (risiko for bias): Risiko for systematiske feil (skjevheter) i resultater pga. svakheter i en studie.

Blindet: Å sørge for at deltakere, behandlere og forskere ikke vet hvilken gruppe deltakerne tilhører.
