# Supplementary material for: Alternative Presentations of Overall and Statistical Uncertainty for Adults’ Understanding of the Results of a Randomized Trial of a Public Health Intervention: Parallel Web-Based Randomized Trials
Source: JMIR Public Health Surveill. 2025 Mar 18;11:e62828. doi: 10.2196/62828 (PMC11962331; doi:10.2196/62828)
Supplement: Multimedia Appendix 2 [file publichealth_v11i1e62828_app2.pdf]

Multimedia appendix 2: Questionnaires in English and Norwegian

English questionnaire

Information about glasses and COVID - a study to improve health communication

Thank you in advance for helping us learn how to improve communication about health research.

If you have any questions about this study, see our webpage or contact the principal investigator, Steven Woloshin at info@messagelab.org.

1. Please provide your unique Prolific ID:

The next section is about wearing glasses and the chance of getting COVID.

Please click on the picture below or **here.**

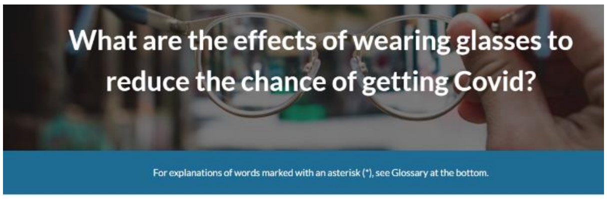

This will open another window with information about the effects of wearing glasses on the chance of getting COVID. Read the information before answering the following questions.

Keep both windows open so you can go back and forth between the information and the questions.

2. Please enter the code that you find in the top left corner of the information page above:

Based on the information you just read, we would like you to answer the following question:

3. If there were a surge of COVID cases in your area, how likely would you be to wear glasses or recommend wearing glasses to reduce the chance of getting COVID?

○ Very likely
○ Likely

○ Unlikely
○ Very unlikely

For questions 4 and 5, you will be reflecting on *the answer you made in question number 3.* Please indicate how much you agree with the following statements:

4. The answer about wearing glasses if there were a surge of COVID was hard for me to give.

○ Strongly agree
○ Agree
○ Disagree
○ Strongly disagree

5. The information in the summary helped me make an informed decision about wearing glasses if there were a surge of COVID

○ Strongly agree
○ Agree
○ Disagree
○ Strongly disagree

6. I wear glasses almost all the time

○ True
○ False

Based on the information provided in the text:

7. If there were **very few** COVID cases in your area, how likely would you be to wear glasses or recommend wearing glasses to reduce the chance of getting COVID?

○ Very likely
○ Likely
○ Unlikely
○ Very unlikely

8. What is the possible effect of wearing glasses on your chance of getting COVID?

○ Lowers the chance a lot
○ Lowers the chance a little
○ Has no effect
○ Increases the chance a little

○ Increases the chance a lot

9. How sure are you about the effect of wearing glasses on your chance of getting COVID?

○ Very sure
○ Mixed but more sure than unsure
○ Mixed but more unsure than sure
○ Very unsure

10. Which of the following statements is **most consistent** with the information provided?

Wearing glasses…
○ May ***reduce*** the chance of COVID a little, but might ***reduce it a lot***
○ May ***reduce*** the chance of COVID a little, but might ***have no effect***
○ May ***reduce*** the chance of COVID a little, but might ***increase it a little***
○ May ***increase*** the chance of COVID it a little, but might ***increase it a lot***
○ Don't know

11. **How likely** do you think it is that wearing glasses to reduce the chance of getting COVID can cause any important **harms**?

○ Very likely
○ Likely
○ Unlikely
○ Very unlikely

12. **How sure** are you about whether wearing glasses to reduce COVID can cause important **harms**?

○ Very sure
○ Somewhat sure
○ Somewhat unsure
○ Very unsure

Please indicate how much you agree with the following statements:

13. This information seems like a **trustworthy** summary of what is known about the effects of wearing glasses to reduce the chance of getting COVID.

○ Strongly agree
○ Agree
○ Disagree

○ Strongly disagree

14. The summary gives me enough information to understand what is known about the effects of wearing glasses to reduce the chance of getting COVID.

○ Strongly agree
○ Agree
○ Disagree
○ Strongly disagree

15. **Not enough is known** to be sure about the effects of wearing glasses to reduce the chance of getting COVID.

○ Strongly agree
○ Agree
○ Disagree
○ Strongly disagree

16. When making an important health decision, uncertainty makes me uneasy, anxious, or stressed.

○ Strongly agree
○ Agree
○ Disagree
○ Strongly disagree

Please indicate what you think about this information:

17. I think the information about whether wearing glasses **affects the chance** of getting COVID was...

○ Very clear
○ Clear
○ Unclear
○ Very unclear

18. I think the information about whether wearing glasses to prevent COVID **has important harms** was...

○ Very clear
○ Clear
○ Unclear
○ Very unclear

19. If you were making a decision about wearing glasses to prevent COVID, would you find the information we showed you helpful?

- Very helpful
- Helpful
- Unhelpful
- Very unhelpful

20. Say you knew someone who heard that wearing glasses might affect your chance of getting COVID. How likely would you be to share the information you just saw with them?

- Definitely yes
- Probably yes
- Probably no
- Definitely no

Please answer the following questions about yourself. This will help us make sense of the study when analyzing the results.

21. I rarely wear glasses

- True
- False

22. What is the highest education level you have completed?

- Some secondary/high school
- Secondary/High school graduate
- Some college or university
- College or university graduate
- Graduate school or professional school graduate

23. How worried are you about getting COVID?

- Extremely worried
- Very worried
- A little worried
- Not worried

24. How important is it for you to take actions to reduce your chance of getting COVID?

- Extremely important
- Very important
- Important
- A little important
- Not important

Lastly, please answer the following three questions

25. A person taking Drug A has a 1% chance of having an allergic reaction. If 1,000 people take Drug A, how many would you expect to have an allergic reaction (**enter a *number* in the box**)?

26. A person taking Drug B has a 1 in 1,000 chance of an allergic reaction. What percent of people taking Drug B will have an allergic reaction?
(**enter a *percentage* in the box**)?

27. Imagine that you flip a coin 1,000 times. What is your best guess about how many times the coin would come up heads in 1,000 flips?
(**enter a *number* in the box**)?

100%

Done

Powered by
SurveyMonkey

Norwegian questionnaire

Basert på denne informasjonen:

1. Hvis det var en økt forekomst av koronatilfeller i ditt nærområde, hvor sannsynlig er det at du ville brukt briller eller anbefalt andre å bruke briller for å minske sjansen for å få korona?

☐ Veldig sannsynlig
☐ Sannsynlig
☐ Usannsynlig
☐ Veldig usannsynlig

I de neste tre spørsmålene skal du reflektere over svaret ditt i forrige spørsmål. Angi <u>hvor enig du er</u> i de følgende påstandene:

2. Avgjørelsen om å bruke briller dersom det var økt forekomst av koronatilfeller var vanskelig for meg å ta.

☐ Helt enig
☐ Enig
☐ Uenig
☐ Sterkt uenig

3. Jeg føler jeg tok en informert beslutning om å bruke briller hvis det var økt forekomst av koronatilfeller.

☐ Helt enig
☐ Enig
☐ Uenig
☐ Sterkt uenig

4. Jeg er fornøyd med min avgjørelse om å bruke briller hvis det var økt forekomst av koronatilfeller.

☐ Helt enig
☐ Enig
☐ Uenig
☐ Sterkt uenig

Med bakgrunn i teksten du leste:

5. Hvis det bare var noen få koronatilfeller i ditt nærområde, hvor sannsynlig er det at du ville brukt briller eller anbefalt andre å bruke briller for å minske sjansen for å få korona?

☐ Veldig sannsynlig
☐ Sannsynlig
☐ Usannsynlig
☐ Veldig usannsynlig

6. Hva er effekten av å bruke briller på sjansen for å få korona?

☐ Minsker sjansen mye
☐ Minsker sjansen litt
☐ Har ingen effekt
☐ Øker sjansen litt
☐ Øker sjansen mye

7. Hvor sikker er du på effekten av brillebruk på sjansen for å få korona?

☐ Veldig sikker
☐ Ganske sikker
☐ Ganske usikker
☐ Veldig usikker

8. Hvilket av de følgende utsagnene stemmer best overens med informasjonen som ble gitt?

Bruk av briller…

		Ja	Nei	Vet ikke
a.	Minsker sjansen for korona litt, men kan også minske den mye	☐	☐	☐
b.	Minsker sjansen for korona litt, men kan også ha ingen effekt	☐	☐	☐
c.	Minsker sjansen for korona litt, men kan også øke den litt	☐	☐	☐
d.	Øker sjansen for korona litt, men kan også øke den mye	☐	☐	☐

9. Hvor sannsynlig tror du det er at brillebruk for å minske sjansen for å få korona kan forårsake betydelige skader?

☐ Veldig sannsynlig
☐ Sannsynlig
☐ Usannsynlig
☐ Veldig usannsynlig

10. Hvor sikker er du på hvorvidt brillebruk for å redusere korona kan forårsake betydelig skade?

☐ Veldig sikker
☐ Ganske sikker
☐ Ganske usikker
☐ Veldig usikker

Angi hvor enig du er i de følgende påstandene:

11. Denne informasjonen er en pålitelig oppsummering av det man vet om effekten av brillebruk for å minske sjansen for å få korona.

☐ Helt enig
☐ Enig
☐ Uenig
☐ Sterkt uenig

12. Denne informasjonen er en tilstrekkelig oppsummering av det man vet om effekten av brillebruk for å minske sjansen for å få korona.

☐ Helt enig
☐ Enig
☐ Uenig
☐ Sterkt uenig

13. Man vet ikke nok til å være sikre på effekten av brillebruk for å minske sjansen for å få korona.

☐ Helt enig
☐ Enig
☐ Uenig
☐ Sterkt uenig

14. Usikkerhet gjør meg urolig, engstelig eller stresset.

☐ Helt enig
☐ Enig
☐ Uenig
☐ Sterkt uenig

Hva synes du om denne informasjonen:

15. Jeg synes informasjonen om i hvilken grad brillebruk har noe å si for sjansen til å få korona var:

☐ Veldig tydelig
☐ Tydelig
☐ Utydelig
☐ Veldig utydelig

16. Jeg synes informasjonen om i hvilken grad brillebruk mot korona innebærer betydelige skader var:

☐ Veldig tydelig
☐ Tydelig
☐ Utydelig
☐ Veldig utydelig

17. Alt i alt synes jeg informasjonen om brillebruk mot korona var:

☐ Veldig nyttig
☐ Nyttig
☐ Unyttig
☐ Veldig unyttig

18. La oss si at du kjenner noen som hadde hørt at brillebruk kan påvirke sannsynligheten for å få korona. Hvor sannsynlig er det at du vil dele den informasjonen du nettopp fikk med dem?

☐ Helt sikkert
☐ Sannsynligvis
☐ Sannsynligvis ikke
☐ Helt sikkert ikke

Vi setter pris på om du svarer på følgende spørsmål om deg:

19. Hvor bor du?

☐ Norge
☐ USA

20. Hvor gammel er du?

☐ Mellom 18-24 år
☐ Mellom 25-34 år
☐ Mellom 35-44 år
☐ Mellom 45-54 år
☐ Mellom 55-64 år
☐ Mellom 65-74 år

21. Hva er den høyeste utdanningen du har gjennomført?

☐ Ungdomsskole og noe av videregående skole
☐ Uteksaminert fra videregående skole
☐ Noen år på universitet eller høyskole
☐ Uteksaminert fra bachelorstudium på universitet eller høyskole
☐ Uteksaminert fra master eller profesjonsstudium på universitet eller høyskole

22. Hvor bekymret er du for å få korona?

☐ Svært bekymret
☐ Ganske bekymret
☐ Ikke veldig bekymret
☐ Ikke bekymret

23. Hvor viktig er det for deg å ta forhåndsregler for å minske din sjanse for å få korona?

☐ Ekstremt viktig
☐ Ganske viktig
☐ Ikke veldig viktig
☐ Ikke viktig

Til slutt ber vi deg om å svare på de følgende tre spørsmålene:

24. En person som tar et medikament A har 1 % sannsynlighet for å få en allergisk reaksjon. Hvis 1 000 personer tar medikament A, hvor mange vil du forvente får en allergisk reaksjon?

___ av 1,000 person(er)

25. En person som tar et medikament B har en tusendels sjanse for å få en allergisk reaksjon. Hvor mange prosent av de som tar medikament B vil få en allergisk reaksjon?

__ %

26. Forestill deg at du slår mynt og kron 1 000 ganger. Hvor mange ganger tror du at du ville fått kron?

___ ganger av 1,000
