# Supplementary material for: Alternative Presentations of Overall and Statistical Uncertainty for Adults’ Understanding of the Results of a Randomized Trial of a Public Health Intervention: Parallel Web-Based Randomized Trials
Source: JMIR Public Health Surveill. 2025 Mar 18;11:e62828. doi: 10.2196/62828 (PMC11962331; doi:10.2196/62828)
Supplement: Multimedia Appendix 3 [file publichealth_v11i1e62828_app3.pdf]

Multimedia appendix 3 – Information provided to the participants in the trials

U.S. trial

In the Prolific panel:

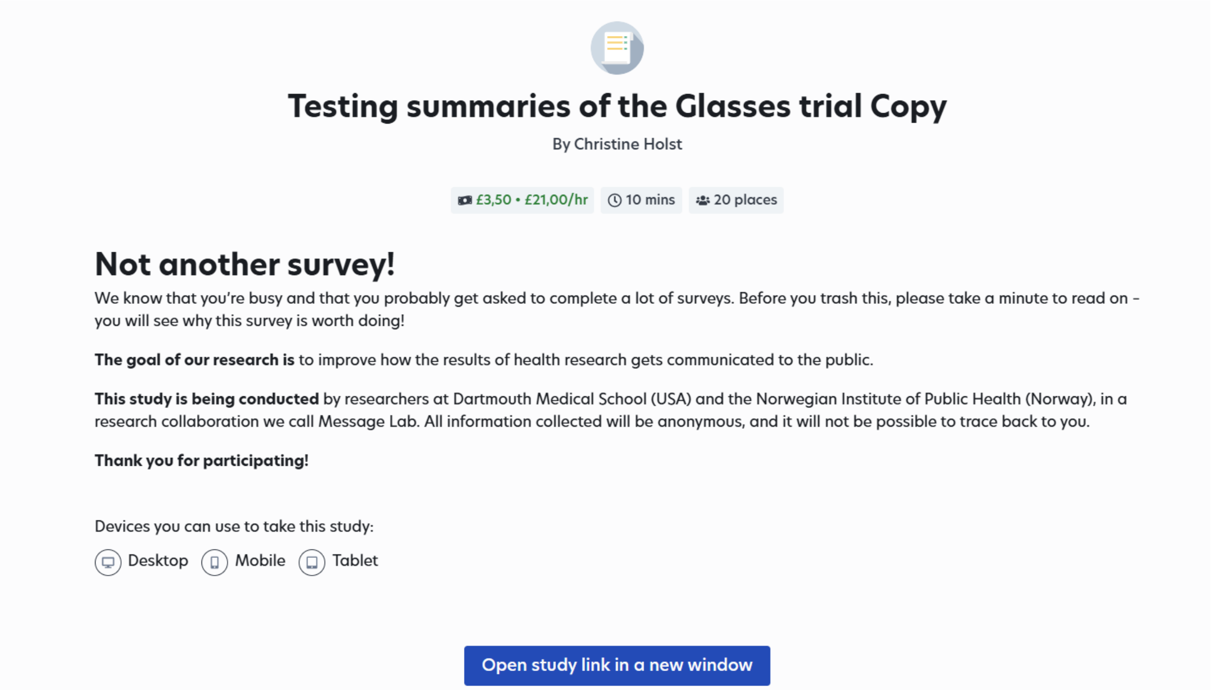

Study link (surveymonkey): https://www.surveymonkey.com/r/D6S3JDL

Information about glasses and COVID - a study to improve health communication

Thank you in advance for helping us learn how to improve communication about health research.

If you have any questions about this study, see our webpage or contact the principal investigator, Steven Woloshin at info@messagelab.org.

1. Please provide your unique Prolific ID:

The next section is about wearing glasses and the chance of getting COVID.

Please click on the picture below or **here.**

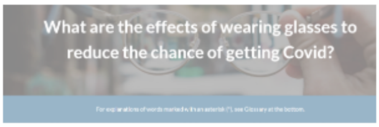

This will open another window with information about the effects of wearing glasses on the chance of getting COVID. Read the information before answering the following questions.

Keep both windows open so you can go back and forth between the information and the questions.

2. Please enter the code that you find in the top left corner of the information page above:

Information provided on the webpage link: https://www.messagelab.org/glasses-summaries-trial

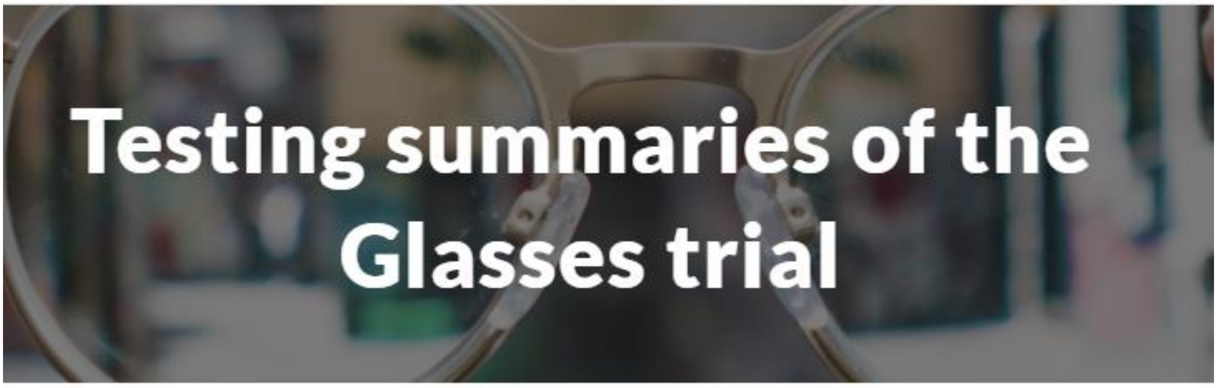

Aim of this study

We're testing ways to communicate the results of a trial of wearing glasses to reduce the chance of getting COVID.

Why is this important?

Clearly communicating the results of research can help people make informed decisions about their health.

Why glasses?

During the pandemic, some researchers were concerned that COVID might infect people through the tear ducts (parts of the eyes, near the nose). Masks don't protect the tear ducts. But glasses might.

Evidence that glasses might help reduce the chance of getting COVID was published in a systematic review. But the evidence was limited: The studies in the review tested different types of eye protection (e.g., face shields and goggles) used by healthcare workers, and were not randomized studies (i.e., true experiments), which are needed to demonstrate an effect.

To find out if wearing glasses reduces the chance of getting COVID, researchers at the Norwegian Institute of Public Health carried out a randomized study. We are using the results from that study, to test different ways of communicating them.

Researchers and funding

This study is being conducted by the Message Lab run by researchers at Dartmouth Medical School and the Norwegian Institute of Public Health.

The Norwegian Institute of Public Health is funding the study.

Questions about this study
Contact principle investigator:
Steven Woloshin
info@messagelab.org

DARTMOUTH

NIPH
CENTRE FOR EPIDEMIC INTERVENTIONS RESEARCH

Norway trial:

In Opinion panel:

Testing av forståelse av forskningsresultater

Denne undersøkelsen dreier seg om hvordan du oppfatter forskningsresultater.

Målet med denne studien er å kunne finne måter å kommunisere resultater fra helseforskning på best mulig måte til befolkningen. Vi håper du kan bidra ved å svare på denne undersøkelsen.

Forskere ved Dartmouth Medical School (USA) og Folkehelseinstituttet gjennomfører studien gjennom et forskningssamarbeid vi kaller «Message Lab».

Alle opplysninger vi samler inn om deg er anonyme, og det er ikke mulig å spore opplysningene tilbake til deg.

Vi vil vise deg en tekst, og stille deg spørsmål for å se om teksten var klar og tydelig. Hele undersøkelsen vil ta rundt 10 minutter. Du kan hoppe over spørsmål om du vil, men vi håper du har anledning til å gjennomføre hele undersøkelsen.

Du kan du lese mer om studien her. Hvis du har spørsmål, ta gjerne kontakt med forsker Christine Holst ved Senter for forskning på epidemitiltak, Folkehelseinstituttet på info@messagelab.org.

<< >>

Information provided on the webpage link:

https://www.messagelab.org/norsk-informasjon-briller-studien

Testing av forståelse av forskningsresultater

Hva gjør vi i denne studien?

Vi tester forskjellige måter å formidle forskningsresultatene fra Koronabrillestudien på. Der ble det ble undersøkt om briller og solbriller kunne beskytte mot koronasmitte.

Hvorfor er dette viktig?

God formidling av forskningsresultater kan hjelpe befolkningen til å ta informerte beslutninger om egen helse.

Hvorfor briller?

Under pandemien var noen forskere bekymret for at folk kunne bli smittet av korona gjennom tårekanalene. Munnbind beskytter ikke tårekanalene, men briller kan kanskje gjøre det. I en systematisk kunnskapsoppsummering, skrev forskerne at øyebeskyttelse, briller inkludert, kunne beskytte mot korona. Men studiene de inkluderte i oppsummeringen ikke var randomiserte studier.

Forskere ved Folkehelseinstituttet gjennomførte derfor en randomisert studie for å undersøke om briller kunne brukes i tillegg til munnbind for å beskytte mot korona. Det er formidling av disse forskningsresultatene vi tester i denne studien.

Gjennomføring og finansiering

Folkehelseinstituttet gjennomfører og finansierer denne studien.
Vi samarbeider med Dartmouth Medical School i Message Lab.

Har du spørsmål om studien?
Kontakt forsker ved FHI:
Christine Holst
info@messagelab.org

DARTMOUTH

FHI
SENTER FOR FORSKNING PÅ EPIDEMITILTAK
