# Supplementary material for: Alternative Presentations of Overall and Statistical Uncertainty for Adults’ Understanding of the Results of a Randomized Trial of a Public Health Intervention: Parallel Web-Based Randomized Trials
Source: JMIR Public Health Surveill. 2025 Mar 18;11:e62828. doi: 10.2196/62828 (PMC11962331; doi:10.2196/62828)
Supplement: Multimedia Appendix 4 [file publichealth_v11i1e62828_app4.pdf]

Multimedia appendix 4 – Additional tables and figures

Additional table S1. Characteristics of Norwegian participants

	Trial arm						
	**Overall uncertainty not shown, margin of error not shown**	**Overall uncertainty not shown, margin of error shown**	**Plain language, margin of error not shown**	**Plain language, margin of error shown**	**GRADE language, margin of error not shown**	**GRADE language, margin of error shown**	**Total**
**N**	80 (18%)	71 (16%)	76 (17%)	79 (17%)	71 (16%)	75 (17%)	452 (100%)
**Age (years)**	47 (18)	45 (18)	46 (17)	43 (17)	45 (17)	45 (15)	45 (17)
**Age group (years)**							
**18 to 29**	18 (22%)	18 (25%)	19 (25%)	22 (28%)	17 (24%)	15 (20%)	109 (24%)
**30 to 39**	11 (14%)	12 (17%)	8 (11%)	17 (22%)	11 (15%)	15 (20%)	74 (16%)
**40 to 49**	15 (19%)	12 (17%)	14 (18%)	10 (13%)	17 (24%)	13 (17%)	81 (18%)
**50 to 59**	15 (19%)	11 (15%)	20 (26%)	14 (18%)	11 (15%)	17 (23%)	88 (19%)
**60 to 69**	13 (16%)	9 (13%)	9 (12%)	9 (11%)	6 (8%)	13 (17%)	59 (13%)
**≥70**	8 (10%)	9 (13%)	6 (8%)	7 (9%)	9 (13%)	2 (3%)	41 (9%)
**Sex**							
**Female**	40 (50%)	38 (54%)	36 (47%)	38 (48%)	38 (54%)	36 (48%)	226 (50%)
**Male**	40 (50%)	33 (46%)	40 (53%)	41 (52%)	33 (46%)	39 (52%)	226 (50%)
**Administrative region[1]**							
**Agder**	6 (8%)	3 (4%)	5 (7%)	3 (4%)	6 (8%)	5 (7%)	28 (6%)
**Innlandet**	6 (8%)	6 (8%)	6 (8%)	6 (8%)	3 (4%)	4 (5%)	31 (7%)
**Møre og Romsdal**	5 (6%)	2 (3%)	3 (4%)	5 (6%)	2 (3%)	5 (7%)	22 (5%)
**Nordland**	3 (4%)	5 (7%)	3 (4%)	2 (3%)	2 (3%)	1 (1%)	16 (4%)
**Oslo**	13 (16%)	11 (15%)	10 (13%)	14 (18%)	11 (15%)	9 (12%)	68 (15%)
**Rogaland**	2 (2%)	8 (11%)	7 (9%)	5 (6%)	5 (7%)	7 (9%)	34 (8%)
**Troms og Finnmark**	6 (8%)	1 (1%)	4 (5%)	3 (4%)	1 (1%)	4 (5%)	19 (4%)
**Trøndelag**	7 (9%)	2 (3%)	7 (9%)	6 (8%)	9 (13%)	7 (9%)	38 (8%)
**Vestfold og Telemark**	7 (9%)	3 (4%)	6 (8%)	3 (4%)	4 (6%)	10 (13%)	33 (7%)
**Vestland**	5 (6%)	10 (14%)	7 (9%)	5 (6%)	12 (17%)	7 (9%)	46 (10%)
**Viken**	20 (25%)	20 (28%)	18 (24%)	27 (34%)	16 (23%)	16 (21%)	117 (26%)
**Employment status**							
**Full-time**	38 (48%)	37 (52%)	31 (41%)	31 (39%)	35 (49%)	37 (49%)	209 (46%)
**Part-time**	6 (8%)	9 (13%)	3 (4%)	7 (9%)	5 (7%)	4 (5%)	34 (8%)
**Welfare[2]**	17 (21%)	15 (21%)	22 (29%)	22 (28%)	21 (30%)	17 (23%)	114 (25%)
**Student**	9 (11%)	6 (8%)	11(14%)	11 (14%)	5 (7%)	7 (9%)	49 (11%)
**Self-employed**	6 (8%)	2 (3%)	3 (4%)	2 (3%)	2 (3%)	7 (9%)	22 (5%)
**Other/Missing**	4 (5%)	2 (3%)	6 (8%)	6 (8%)	3 (4%)	3 (4%)	24 (5%)
**Highest education**							
**Lower secondary school (ages 13–16)**	8 (10%)	2 (3%)	8 (11%)	3 (4%)	3 (4%)	9 (12%)	33 (7%)
**Upper secondary school (ages 16–19)**	22 (28%)	24 (34%)	21 (28%)	22 (28%)	17 (24%)	25 (33%)	131 (29%)
**Some university or college**	16 (20%)	13 (18%)	19 (25%)	15 (19%)	9 (13%)	19 (25%)	91 (20%)
**Bachelor's degree**	22 (28%)	23 (32%)	18 (24%)	23 (29%)	26 (37%)	12 (16%)	124 (27%)
**Master's degree or equivalent**	12 (15%)	9 (13%)	10 (13%)	16 (20%)	16 (23%)	10 (13%)	73 (16%)

Data are mean (SD) or N (%). [1]Fylke (2020 definition), [2]Includes participants in any welfare program; maternity leave, pensioner, unemployment benefits

Additional table S2. Characteristics of US participants

	Trial arm						
	No explicit language, margin of error not shown	No explicit language, margin of error shown	Plain language, margin of error not shown	Plain language, margin of error shown	GRADE language, margin of error not shown	GRADE language, margin of error shown	Total
**N**	86 (16%)	88 (16%)	99 (18%)	90 (17%)	79 (15%)	101 (19%)	543 (100%)
**Age (years)**	38 (14)	37 (13)	38 (13)	39 (13)	40 (14)	36 (12)	38 (13)
**Age group (years)**							
**18 to 29**	24 (28%)	28 (32%)	26 (26%)	24 (27%)	20 (25%)	33 (33%)	155 (29%)
**30 to 39**	34 (40%)	28 (32%)	35 (35%)	32 (36%)	28 (35%)	38 (38%)	195 (36%)
**40 to 49**	9 (10%)	17 (19%)	22 (22%)	20 (22%)	15 (19%)	15 (15%)	98 (18%)
**50 to 59**	2 (2%)	9 (10%)	3 (3%)	4 (4%)	4 (5%)	5 (5%)	27 (5%)
**60 to 69**	17 (20%)	4 (5%)	12 (12%)	8 (9%)	8 (10%)	9 (9%)	58 (11%)
**≥70**	0 (0%)	2 (2%)	1 (1%)	2 (2%)	4 (5%)	1 (1%)	10 (2%)
**Sex**							
**Female**	37 (43%)	40 (45%)	41 (41%)	42 (47%)	42 (53%)	60 (59%)	262 (48%)
**Male**	49 (57%)	48 (55%)	58 (59%)	48 (53%)	37 (47%)	41 (41%)	281 (52%)
**Born in the US**							
**No**	7 (8%)	6 (7%)	9 (9%)	7 (8%)	11 (14%)	7 (7%)	47 (9%)
**Yes**	79 (92%)	82 (93%)	90 (91%)	83 (92%)	68 (86%)	94 (93%)	496 (91%)
**Language**							
**English**	82 (95%)	84 (95%)	91 (92%)	85 (94%)	75 (95%)	96 (95%)	513 (94%)
**Other**	4 (5%)	4 (5%)	8 (8%)	5 (6%)	4 (5%)	5 (5%)	30 (6%)
**Employment status**							
**Full-time**	32 (37%)	29 (33%)	39 (39%)	40 (44%)	32 (41%)	41 (41%)	213 (39%)
**Part-time**	10 (12%)	19 (22%)	9 (9%)	8 (9%)	8 (10%)	11 (11%)	65 (12%)
**Unpaid[1]**	10 (12%)	7 (8%)	8 (8%)	11 (12%)	10 (13%)	9 (9%)	55 (10%)
**Unemployed[2]**	11 (13%)	15 (17%)	8 (8%)	7 (8%)	3 (4%)	7 (7%)	51 (9%)
**Other**	5 (6%)	5 (6%)	6 (6%)	6 (7%)	3 (4%)	5 (5%)	30 (6%)
**Missing**	18 (21%)	13 (15%)	29 (29%)	18 (20%)	23 (29%)	28 (28%)	129 (24%)
**Student**							
**Yes**	16 (19%)	11 (12%)	12 (12%)	9 (10%)	9 (11%)	10 (10%)	67 (12%)
**No**	54 (63%)	64 (73%)	64 (65%)	62 (69%)	51 (65%)	65 (64%)	360 (66%)
**Missing[3]**	16 (19%)	13 (15%)	23 (23%)	19 (21%)	19 (24%)	26 (26%)	116 (21%)
**Highest education**							
**Some secondary/high school**	5 (6%)	6 (7%)	3 (3%)	5 (6%)	3 (4%)	3 (3%)	25 (5%)
**Secondary/High school graduate**	14 (16%)	17 (19%)	18 (18%)	11 (12%)	10 (13%)	22 (22%)	92 (17%)
**Some college or university**	16 (19%)	30 (34%)	24 (24%)	23 (26%)	19 (24%)	30 (30%)	142 (26%)
**College or university graduate**	33 (38%)	24 (27%)	39 (39%)	40 (44%)	33 (42%)	35 (35%)	204 (38%)
**Graduate school or professional school graduate**	18 (21%)	11 (12%)	15 (15%)	11 (12%)	14 (18%)	11 (11%)	80 (15%)

Data are mean (SD) or N (%). [1]Includes participants who are homemakers, retired, or disabled. [2]Unemployed and seeking work. [3]One participant answered "Spanish". [4]GED/GCSE.

Understanding of overall uncertainty of the effect of wearing glasses on the chance of getting COVID

Additional table S3. Norwegian trial

How sure are you about the effect of wearing glasses on your chance of getting COVID?

Very sure
Mixed but more sure than unsure
Mixed but more unsure than sure (correct)
Very unsure

(N = 452)

		Very sure	Mixed but more sure than unsure	Mixed but more unsure than sure	Very unsure
**Overall uncertainty**	**Margin of Error**	**n/N (%)**	**n/N (%)**	**n/N (%)**	**n/N (%)**
No explicit language	Not shown	9/80 (11.3%)	22/80 (27.5%)	34/80 (42.5%)	15/80 (18.8%)
No explicit language	Shown	7/71 (9.9%)	17/71 (23.9%)	33/71 (46.5%)	14/71 (19.7%)
Plain language	Not shown	4/76 (5.3%)	10/76 (13.2%)	40/76 (52.6%)	22/76 (28.9%)
Plain language	Shown	5/79 (6.3%)	21/79 (26.6%)	29/79 (36.7%)	24/79 (30.4%)
GRADE language	Not shown	5/71 (7.0%)	18/71 (25.4%)	30/71 (42.3%)	18/71 (25.4%)
GRADE language	Shown	6/75 (8.0%)	12/75 (16.0%)	36/75 (48.0%)	21/75 (28.0%)

Additional table S4. US trial

How sure are you about the effect of wearing glasses on your chance of getting COVID?

Very sure
Mixed but more sure than unsure
Mixed but more unsure than sure (correct)
Very unsure

(N = 543)

		Very sure	Mixed but more sure than unsure	Mixed but more unsure than sure	Very unsure
**Overall uncertainty**	**Margin of Error**	**n/N (%)**	**n/N (%)**	**n/N (%)**	**n/N (%)**
No explicit language	Not shown	25/86 (29.1%)	36/86 (41.9%)	18/86 (20.9%)	7/86 (8.1%)
No explicit language	Shown	16/88 (18.2%)	38/88 (43.2%)	19/88 (21.6%)	15/88 (17%)
Plain language	Not shown	18/99 (18.2%)	30/99 (30.3%)	37/99 (37.4%)	14/99 (14.1%)
Plain language	Shown	13/90 (14.4%)	29/90 (32.2%)	25/90 (27.8%)	23/90 (25.6%)
GRADE language	Not shown	13/79 (16.5%)	30/79 (38.0%)	26/79 (32.9%)	10/79 (12.7%)
GRADE language	Shown	19/101 (18.8%)	40/101 (39.6%)	22/101 (21.8%)	20/101 (19.8%)

Understanding certainty of the harm

Additional figure S1

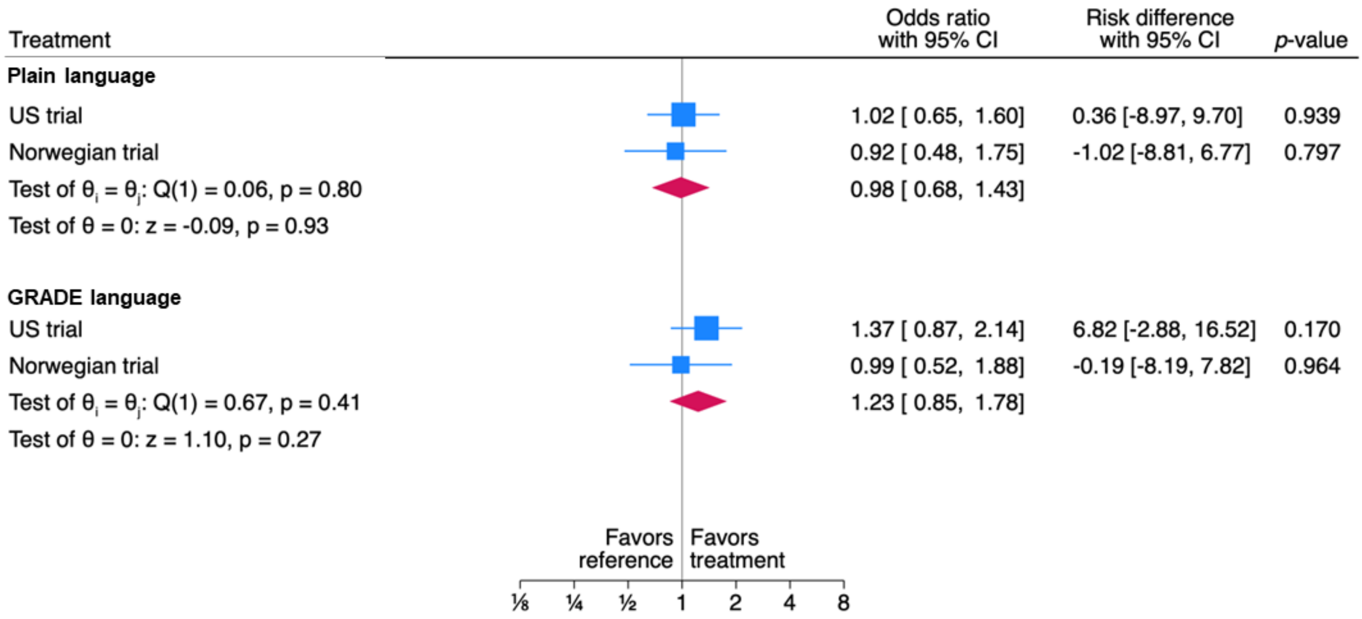

Additional table S5. Risk differences for understanding the certainty of important harms – Norwegian trial

How sure are you about whether wearing glasses to reduce COVID can cause important harms?
Very sure
Somewhat sure (correct)
Somewhat unsure
Very unsure

Overall uncertainty	n/N (%)[1]	Odds ratio [95% CI]	Risk difference [95% CI][2]	p-value
No explicit language	22/151 (14.6%)	1	0	NA
Plain language	21/155 (13.5%)	0.92 [0.48 to 1.75]	-1.02 [-8.81 to 6.77]	0.797
GRADE language	21/146 (14.4%)	0.99 [0.52 to 1.88]	-0.19 [-8.19 to 7.82]	0.964

[1]Number (n) of participants randomized to the intervention (N) answering correctly or as anticipated. [2]Risk differences account for uncertainty on the baseline odds.

Additional table S6. Risk differences for understanding the certainty of important harms – US trial

How sure are you about whether wearing glasses to reduce COVID can cause important harms?
Very sure
Somewhat sure (correct)
Somewhat unsure
Very unsure

Overall uncertainty	n/N (%)[1]	Odds ratio [95% CI]	Risk difference [95% CI][2]	p-value
No explicit language	50/174 (28.7%)	1	0	NA
Plain language	55/189 (29.1%)	1.02 [0.65 to 1.60]	0.36 [-8.97 to 9.70]	0.939
GRADE language	64/180 (35.6%)	1.37 [0.87 to 2.14]	6.82 [-2.88 to 16.52]	0.170

[1]Number (n) of participants randomized to the intervention (N) answering correctly or as anticipated. [2]Risk differences account for uncertainty on the baseline odds.

Understanding the margin of error

Additional table S7. Norwegian trial

Which of the following statements is most consistent with the information provided?

Wearing glasses…
May reduce the chance of COVID a little, but might reduce it a lot
May reduce the chance of COVID a little, but might have no effect
May reduce the chance of COVID a little, but might increase it a little (correct)
May increase the chance of COVID it a little, but might increase it a lot
Don't know

Overall uncertainty	Margin of Error	n/N (%)[1]	Odds ratio [95% CI][2]	Risk difference [95% CI][3]	p-value
No explicit language	Not shown	1/80 (1.2%)	1	0	NA
No explicit language	Shown	24/71 (33.8%)	27.34 [5.06 to 147.75]	49.69 [24.36 to 75.02]	0.001
Plain language	Not shown	2/76 (2.6%)	1.78 [0.23 to 13.80]	1.47 [-3.74 to 6.68]	0.582
Plain language	Shown	21/79 (26.6%)	19.48 [3.60 to 105.49]	34.87 [16.44 to 53.29]	0.001
GRADE language	Not shown	1/71 (1.4%)	1.13 [0.11 to 11.09]	0.24 [-4.36 to 4.84]	0.918
GRADE language	Shown	16/75 (21.3%)	14.70 [2.67 to 80.85]	25.84 [10.42 to 41.27]	0.002

[1]Number (n) of participants randomized to the intervention (N) answering correctly or as anticipated. [2]Odds ratios include the main and interaction effects. [3]Risk differences account for uncertainty on the baseline odds.

Additional table S8. US trial

Which of the following statements is most consistent with the information provided?

Wearing glasses…
May reduce the chance of COVID a little, but might reduce it a lot
May reduce the chance of COVID a little, but might have no effect
May reduce the chance of COVID a little, but might increase it a little (correct)
May increase the chance of COVID it a little, but might increase it a lot
Don't know

Overall uncertainty	Margin of Error	n/N (%)[1]	Odds ratio [95% CI][2]	Risk difference [95% CI][3]	p-value
No explicit language	Not shown	0/86 (0.0%)	1	0	NA
No explicit language	Shown	24/88 (27.3%)	65.71 [3.92 to 1100.73]	37.41 [19.67 to 55.15]	0.004
Plain language	Not shown	2/99 (2.0%)	4.44 [0.21 to 93.68]	1.99 [-1.61 to 5.58]	0.338
Plain language	Shown	32/90 (35.6%)	96.11 [5.77 to 1600.64]	54.98 [31.10 to 78.85]	0.001
GRADE language	Not shown	3/79 (3.8%)	7.92 [0.40 to 155.69]	4.00 [-1.16 to 9.16]	0.173
GRADE language	Shown	21/101 (20.8%)	46.20 [2.75 to 775.33]	26.13 [13.32 to 38.94]	0.008

[1]Number (n) of participants randomized to the intervention (N) answering correctly or as anticipated. [2]Odds ratios include the main and interaction effects. [3]Risk differences account for uncertainty on the baseline odds.

Interest in wearing glasses to reduce Covid risk during a surge in cases

Additional figure S2

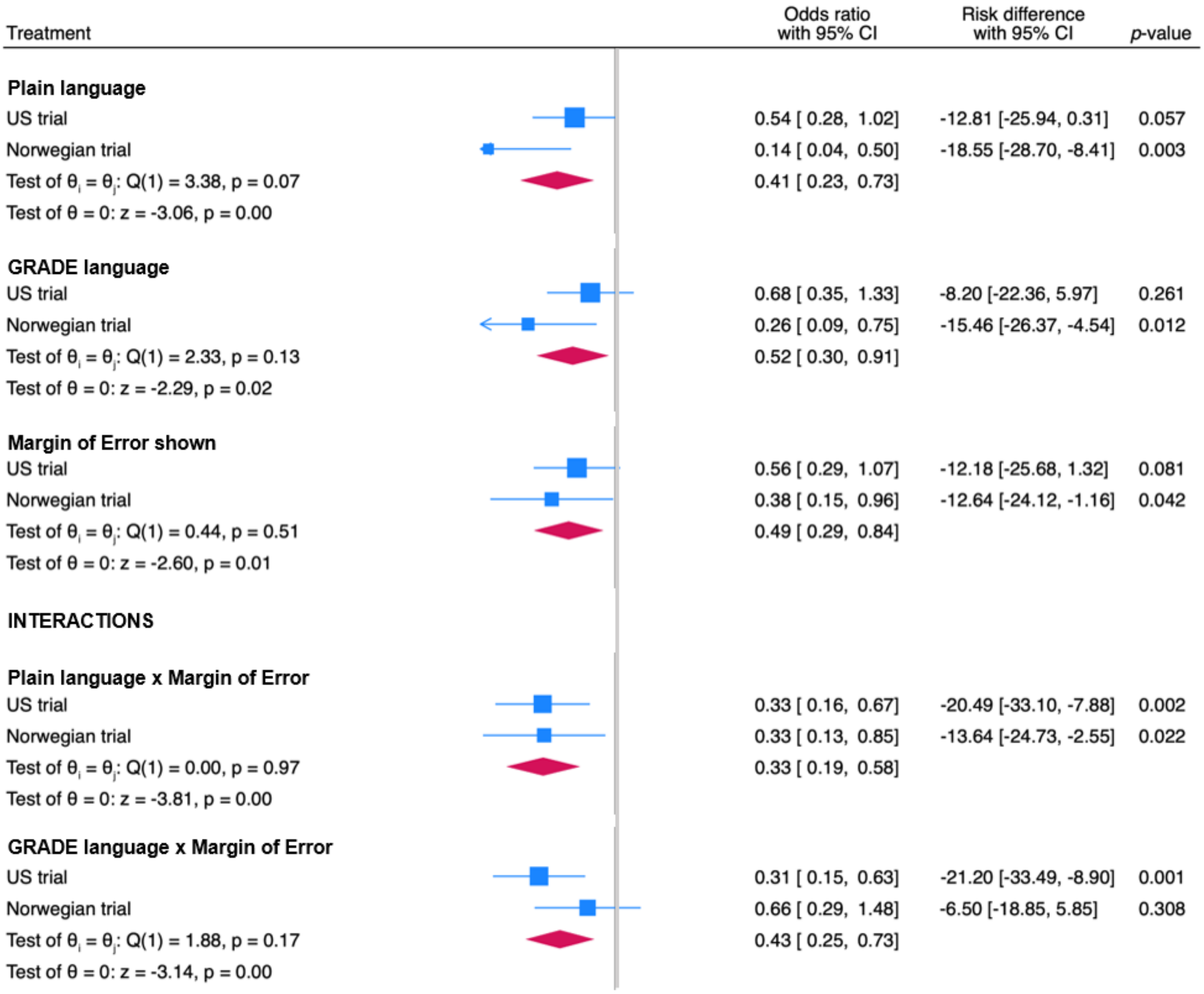

Odds ratios for answering “Very likely” or “Likely” to the question “If there were a surge of COVID cases in your area, how likely would you be to wear glasses or recommend wearing glasses to reduce the chance of getting COVID?”

Risk differences for intended behavior if there was a surge

Additional table S9. Norwegian trial

If there were a surge of COVID cases in your area, how likely would you be to wear glasses or recommend wearing glasses to reduce the chance of getting COVID?

Very likely
Likely
Unlikely
Very unlikely

Overall uncertainty	Margin of Error	n/N (%)[1]	Odds ratio [95% CI][2]	Risk difference [95% CI][3]	p-value
No explicit language	Not shown	18/80 (22.5%)	1	0	NA
No explicit language	Shown	7/71 (9.9%)	0.38 [0.15 to 0.96]	-12.64 [-24.12 to -1.16]	0.042
Plain language	Not shown	3/76 (3.9%)	0.14 [0.04 to 0.50]	-18.55 [-28.70 to -8.41]	0.003
Plain language	Shown	7/79 (8.9%)	0.33 [0.13 to 0.85]	-13.64 [-24.73 to -2.55]	0.022
GRADE language	Not shown	5/71 (7.0%)	0.26 [0.09 to 0.75]	-15.46 [-26.37 to -4.54]	0.012
GRADE language	Shown	12/75 (16.0%)	0.66 [0.29 to 1.48]	-6.50 [-18.85 to 5.85]	0.308

[1]Number (n) of participants randomized to the intervention (N) answering correctly or as anticipated. [2]Odds ratios include the main and interaction effects. [3]Risk differences account for uncertainty on the baseline odds.

Additional table S10. US trial

If there were a surge of COVID cases in your area, how likely would you be to wear glasses or recommend wearing glasses to reduce the chance of getting COVID?

Very likely
Likely
Unlikely
Very unlikely

Overall uncertainty	Margin of Error	n/N (%)[1]	Odds ratio [95% CI][2]	Risk difference [95% CI][3]	p-value
No explicit language	Not shown	31/86 (36.0%)	1	0	NA
No explicit language	Shown	21/88 (23.9%)	0.56 [0.29 to 1.07]	-12.2 [-25.7 to 1.3]	0.081
Plain language	Not shown	23/99 (23.2%)	0.54 [0.28 to 1.02]	-12.8 [-25.9 to 0.3]	0.057
Plain language	Shown	14/90 (15.6%)	0.33 [0.16 to 0.67]	-20.5 [-33.1 to -7.9]	0.002
GRADE language	Not shown	22/79 (27.8%)	0.68 [0.35 to 1.33]	-8.2 [-22.4 to 6.0]	0.261
GRADE language	Shown	15/101 (14.9%)	0.31 [0.15 to 0.63]	-21.2 [-33.5 to -8.9]	0.001

[1]Number (n) of participants randomized to the intervention (N) answering correctly or as anticipated. [2]Odds ratios include the main and interaction effects. [3]Risk differences account for uncertainty on the baseline odds.
.

Interest in wearing glasses to reduce Covid risk if no surge in cases

Additional figure S3

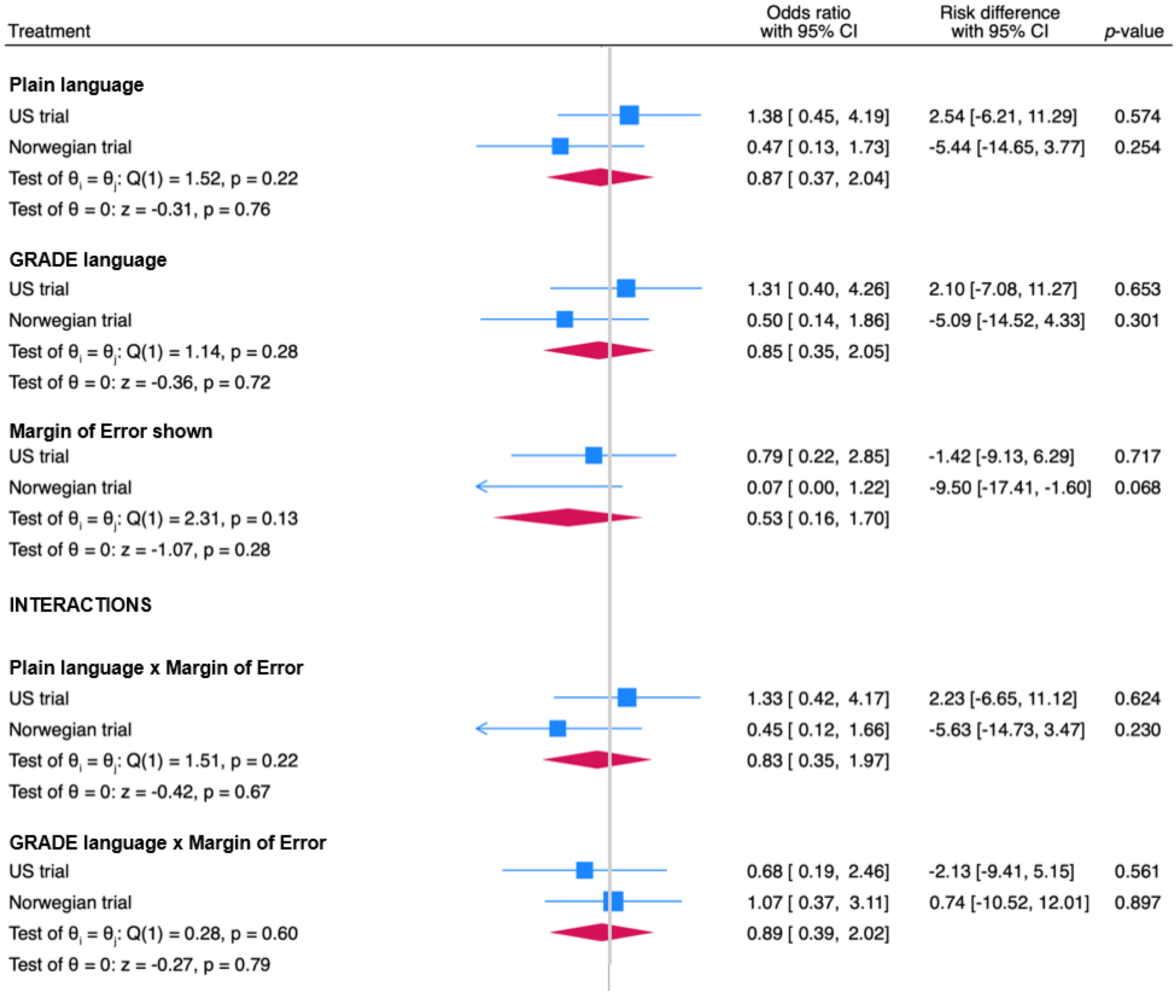

Odds ratios for answering “Very likely” or “Likely” to the question “If there were very few COVID cases in your area, how likely would you be to wear glasses or recommend wearing glasses to reduce the chance of getting COVID?”

Risk differences for perceptions of the helpfulness of the information

Additional table S11. Norwegian trial

If you were making a decision about wearing glasses to prevent COVID, would you find the information we showed you helpful?
Very helpful
Helpful
Unhelpful
Very unhelpful

**Overall uncertainty**	**Margin of Error**	**n/N (%)[1]**	**Odds ratio [95% CI][2]**	**Risk difference [95% CI][3]**	**p-value**
No explicit language	Not shown	52/80 (65.0%)	1	0	NA
No explicit language	Shown	41/71 (57.7%)	0.74 [0.38 to 1.42]	-7.25 [-22.79 to 8.28]	0.361
Plain language	Not shown	40/76 (52.6%)	0.60 [0.31 to 1.14]	-12.37 [-27.71 to 2.97]	0.118
Plain language	Shown	39/79 (49.4%)	0.52 [0.28 to 0.99]	-15.63 [-30.82 to -0.44]	0.047
GRADE language	Not shown	41/71 (57.7%)	0.74 [0.38 to 1.42]	-7.25 [-22.79 to 8.28]	0.361
GRADE language	Shown	41/75 (54.7%)	0.65 [0.34 to 1.24]	-10.33 [-25.70 to 5.03]	0.190

[1]Number (n) of participants randomized to the intervention (N) answering correctly or as anticipated. [2]Odds ratios include the main and interaction effects. [3]Risk differences account for uncertainty on the baseline odds.

Additional table S12. US trial

If you were making a decision about wearing glasses to prevent COVID, would you find the information we showed you helpful?
Very helpful
Helpful
Unhelpful
Very unhelpful

**Overall uncertainty**	**Margin of Error**	**n/N (%)[1]**	**Odds ratio [95% CI][2]**	**Risk difference [95% CI][3]**	**p-value**
No explicit language	Not shown	69/86 (80.2%)	1	0	NA
No explicit language	Shown	58/88 (65.9%)	0.48 [0.24 to 0.95]	-14.3 [-27.3 to -1.3]	0.035
Plain language	Not shown	62/99 (62.6%)	0.41 [0.21 to 0.81]	-17.6 [-30.3 to -4.9]	0.010
Plain language	Shown	60/90 (66.7%)	0.49 [0.25 to 0.98]	-13.6 [-26.4 to -0.7]	0.044
GRADE language	Not shown	55/79 (69.6%)	0.56 [0.28 to 1.15]	-10.6 [-23.8 to 2.6]	0.117
GRADE language	Shown	71/101 (70.3%)	0.58 [0.30 to 1.15]	-9.9 [-22.2 to 2.3]	0.121

[1]Number (n) of participants randomized to the intervention (N) answering correctly or as anticipated. [2]Odds ratios include the main and interaction effects. [3]Risk differences account for uncertainty on the baseline odds.

Risk differences for perceptions of the trustworthiness of the summaries

Additional table S13. Norwegian trial

This information seems like a trustworthy summary of what is known about the effects of wearing glasses to reduce the chance of getting COVID.
Strongly agree
Agree
Disagree
Strongly disagree

Overall uncertainty	Margin of Error	n/N (%)[1]	Odds ratio [95% CI][2]	Risk difference [95% CI][3]	p-value
No explicit language	Not shown	49/80 (61.3%)	1	0	NA
No explicit language	Shown	40/71 (56.3%)	0.82 [0.43 to 1.56]	-4.91 [-20.63 to 10.81]	0.540
Plain language	Not shown	41/76 (53.9%)	0.74 [0.39 to 1.40]	-7.30 [-22.78 to 8.17]	0.357
Plain language	Shown	31/79 (39.2%)	0.41 [0.22 to 0.77]	-22.01 [-37.17 to -6.85]	0.006
GRADE language	Not shown	40/71 (56.3%)	0.82 [0.43 to 1.56]	-4.91 [-20.63 to 10.81]	0.540
GRADE language	Shown	33/75 (44.0%)	0.50 [0.26 to 0.94]	-17.25 [-32.75 to -1.75]	0.032

[1]Number (n) of participants randomized to the intervention (N) answering correctly or as anticipated. [2]Odds ratios include the main and interaction effects. [3]Risk differences account for uncertainty on the baseline odds.

Additional table S14. US trial

This information seems like a trustworthy summary of what is known about the effects of wearing glasses to reduce the chance of getting COVID.
Strongly agree
Agree
Disagree
Strongly disagree

Overall uncertainty	Margin of Error	n/N (%)[1]	Odds ratio [95% CI][2]	Risk difference [95% CI][3]	p-value
No explicit language	Not shown	57/86 (66.3%)	1	0	NA
No explicit language	Shown	60/88 (68.2%)	1.09 [0.58 to 2.05]	1.9 [-12.0 to 15.9]	0.789
Plain language	Not shown	57/99 (57.6%)	0.69 [0.38 to 1.26]	-8.7 [-22.7 to 5.2]	0.226
Plain language	Shown	55/90 (61.1%)	0.80 [0.43 to 1.48]	-5.2 [-19.4 to 9.0]	0.476
GRADE language	Not shown	50/79 (63.3%)	0.88 [0.46 to 1.66]	-3.0 [-17.6 to 11.6]	0.688
GRADE language	Shown	67/101 (66.3%)	1.00 [0.55 to 1.84]	0.1 [-13.5 to 13.7]	0.993

[1]Number (n) of participants randomized to the intervention (N) answering correctly or as anticipated. [2]Odds ratios include the main and interaction effects. [3]Risk differences account for uncertainty on the baseline odds.

Risk differences for perceptions of the sufficiency of the information

Additional table S15. Norwegian trial

The summary gives me enough information to understand what is known about the effects of wearing glasses to reduce the chance of getting COVID.
Strongly agree
Agree
Disagree
Strongly disagree

Overall uncertainty	Margin of Error	n/N (%)[1]	Odds ratio [95% CI][2]	Risk difference [95% CI][3]	p-value
No explicit language	Not shown	51/80 (63.7%)	1	0	NA
No explicit language	Shown	36/71 (50.7%)	0.58 [0.30 to 1.12]	-13.05 [-28.74 to 2.65]	0.107
Plain language	Not shown	42/76 (55.3%)	0.70 [0.37 to 1.33]	-8.49 [-23.85 to 6.87]	0.281
Plain language	Shown	42/79 (53.2%)	0.65 [0.34 to 1.22]	-10.59 [-25.82 to 4.65]	0.177
GRADE language	Not shown	42/71 (59.2%)	0.82 [0.43 to 1.59]	-4.60 [-20.14 to 10.95]	0.562
GRADE language	Shown	30/75 (40.0%)	0.38 [0.20 to 0.73]	-23.75 [-39.04 to -8.46]	0.003

[1]Number (n) of participants randomized to the intervention (N) answering correctly or as anticipated. [2]Odds ratios include the main and interaction effects. [3]Risk differences account for uncertainty on the baseline odds.

Additional table S16. US trial

The summary gives me enough information to understand what is known about the effects of wearing glasses to reduce the chance of getting COVID.
Strongly agree
Agree
Disagree
Strongly disagree

Overall uncertainty	Margin of Error	n/N (%)[1]	Odds ratio [95% CI][2]	Risk difference [95% CI][3]	p-value
No explicit language	Not shown	56/86 (65.1%)	1	0	NA
No explicit language	Shown	53/88 (60.2%)	0.81 [0.44 to 1.50]	-4.9 [-19.2 to 9.5]	0.505
Plain language	Not shown	53/99 (53.5%)	0.62 [0.34 to 1.12]	-11.6 [-25.7 to 2.5]	0.111
Plain language	Shown	49/90 (54.4%)	0.64 [0.35 to 1.18]	-10.7 [-25.1 to 3.7]	0.150
GRADE language	Not shown	47/79 (59.5%)	0.79 [0.42 to 1.48]	-5.6 [-20.4 to 9.2]	0.457
GRADE language	Shown	65/101 (64.4%)	0.97 [0.53 to 1.77]	-0.8 [-14.5 to 13.0]	0.914

[1]Number (n) of participants randomized to the intervention (N) answering correctly or as anticipated. [2]Odds ratios include the main and interaction effects. [3]Risk differences account for uncertainty on the baseline odds. The correct answer was " Agree or strongly agree".

Risk differences for perceptions of the clarity of the information about benefit of wearing glasses

Additional table S17. Norwegian trial

I think the information about whether wearing glasses affects the chance of getting COVID was...
Very clear
Clear
Unclear
Very unclear

Overall uncertainty	Margin of Error	n/N (%)[1]	Odds ratio [95% CI][2]	Risk difference [95% CI][3]	p-value
No explicit language	Not shown	55/80 (68.8%)	1	0	NA
No explicit language	Shown	39/71 (54.9%)	0.55 [0.28 to 1.08]	-13.82 [-29.22 to 1.58]	0.082
Plain language	Not shown	40/76 (52.6%)	0.51 [0.26 to 0.97]	-16.12 [-31.26 to -0.98]	0.040
Plain language	Shown	50/79 (63.3%)	0.78 [0.41 to 1.51]	-5.46 [-20.16 to 9.24]	0.468
GRADE language	Not shown	46/71 (64.8%)	0.84 [0.42 to 1.65]	-3.96 [-19.01 to 11.09]	0.606
GRADE language	Shown	40/75 (53.3%)	0.52 [0.27 to 1.00]	-15.42 [-30.60 to -0.23]	0.050

[1]Number (n) of participants randomized to the intervention (N) answering correctly or as anticipated. [2]Odds ratios include the main and interaction effects. [3]Risk differences account for uncertainty on the baseline odds.

Additional table S18. US trial

I think the information about whether wearing glasses affects the chance of getting COVID was...
Very clear
Clear
Unclear
Very unclear

Overall uncertainty	Margin of Error	n/N (%)[1]	Odds ratio [95% CI][2]	Risk difference [95% CI][3]	p-value
No explicit language	Not shown	67/86 (77.9%)	1	0	NA
No explicit language	Shown	63/88 (71.6%)	0.71 [0.36 to 1.42]	-6.3 [-19.2 to 6.6]	0.339
Plain language	Not shown	65/99 (65.7%)	0.54 [0.28 to 1.05]	-12.3 [-25.1 to 0.6]	0.068
Plain language	Shown	53/90 (58.9%)	0.41 [0.21 to 0.79]	-19.0 [-32.4 to -5.6]	0.007
GRADE language	Not shown	48/79 (60.8%)	0.44 [0.22 to 0.87]	-17.1 [-31.0 to -3.3]	0.018
GRADE language	Shown	72/101 (71.3%)	0.70 [0.36 to 1.37]	-6.6 [-19.1 to 5.8]	0.303

[1]Number (n) of participants randomized to the intervention (N) answering correctly or as anticipated. [2]Odds ratios include the main and interaction effects. [3]Risk differences account for uncertainty on the baseline odds. Percentages are for those saying " Clear or very clear"

Risk differences for perceptions of the clarity of the information about important harms

Additional table S19. Norwegian trial

I think the information about whether wearing glasses to prevent COVID has important harms was...
Very clear
Clear
Unclear
Very unclear

Overall uncertainty	Margin of Error	n/N (%)[1]	Odds ratio [95% CI][2]	Risk difference [95% CI][3]	p-value
No explicit language	Not shown	49/80 (61.3%)	1	0	NA
No explicit language	Shown	46/71 (64.8%)	1.16 [0.60 to 2.26]	3.54 [-11.87 to 18.95]	0.653
Plain language	Not shown	39/76 (51.3%)	0.67 [0.35 to 1.26]	-9.93 [-25.43 to 5.57]	0.212
Plain language	Shown	31/79 (39.2%)	0.41 [0.22 to 0.77]	-22.01 [-37.17 to -6.85]	0.006
GRADE language	Not shown	43/71 (60.6%)	0.97 [0.50 to 1.87]	-0.69 [-16.28 to 14.91]	0.931
GRADE language	Shown	39/75 (52.0%)	0.69 [0.36 to 1.30]	-9.25 [-24.80 to 6.30]	0.246

[1]Number (n) of participants randomized to the intervention (N) answering correctly or as anticipated. [2]Odds ratios include the main and interaction effects. [3]Risk differences account for uncertainty on the baseline odds.

Additional table S20. US trial

I think the information about whether wearing glasses to prevent COVID has important harms was...
Very clear
Clear
Unclear
Very unclear

Overall uncertainty	Margin of Error	n/N (%)[1]	Odds ratio [95% CI][2]	Risk difference [95% CI][3]	p-value
No explicit language	Not shown	70/86 (81.4%)	1	0	NA
No explicit language	Shown	57/88 (64.8%)	0.42 [0.21 to 0.84]	-16.6 [-29.6 to -3.7]	0.015
Plain language	Not shown	69/99 (69.7%)	0.53 [0.26 to 1.05]	-11.7 [-23.9 to 0.5]	0.069
Plain language	Shown	70/90 (77.8%)	0.80 [0.38 to 1.67]	-3.6 [-15.5 to 8.3]	0.552
GRADE language	Not shown	56/79 (70.9%)	0.56 [0.27 to 1.15]	-10.5 [-23.5 to 2.5]	0.115
GRADE language	Shown	75/101 (74.3%)	0.66 [0.33 to 1.33]	-7.1 [-19.0 to 4.7]	0.245

[1]Number (n) of participants randomized to the intervention (N) answering correctly or as anticipated. [2]Odds ratios include the main and interaction effects. [3]Risk differences account for uncertainty on the baseline odds.

Risk differences for the likelihood that participants would share the information

Additional table S21. Norwegian trial

Say you knew someone who heard that wearing glasses might affect your chance of getting COVID. How likely would you be to share the information you just saw with them?
Definitely yes
Probably yes
Probably no
Definitely no

Overall uncertainty	Margin of Error	n/N (%)[1]	Odds ratio [95% CI][2]	Risk difference [95% CI][3]	p-value
No explicit language	Not shown	49/80 (61.3%)	1	0	NA
No explicit language	Shown	45/71 (63.4%)	1.09 [0.57 to 2.12]	2.13 [-13.35 to 17.61]	0.788
Plain language	Not shown	34/76 (44.7%)	0.51 [0.27 to 0.97]	-16.51 [-31.97 to -1.06]	0.040
Plain language	Shown	38/79 (48.1%)	0.59 [0.31 to 1.10]	-13.15 [-28.49 to 2.19]	0.097
GRADE language	Not shown	42/71 (59.2%)	0.92 [0.48 to 1.76]	-2.10 [-17.74 to 13.55]	0.793
GRADE language	Shown	41/75 (54.7%)	0.76 [0.40 to 1.45]	-6.58 [-22.10 to 8.94]	0.407

[1]Number (n) of participants randomized to the intervention (N) answering correctly or as anticipated. [2]Odds ratios include the main and interaction effects. [3]Risk differences account for uncertainty on the baseline odds.

Additional table S22. US trial

Say you knew someone who heard that wearing glasses might affect your chance of getting COVID. How likely would you be to share the information you just saw with them?
Definitely yes
Probably yes
Probably no
Definitely no

Overall uncertainty	Margin of Error	n/N (%)[1]	Odds ratio [95% CI][2]	Risk difference [95% CI][3]	p-value
No explicit language	Not shown	65/86 (75.6%)	1	0	NA
No explicit language	Shown	55/88 (62.5%)	0.54 [0.28 to 1.04]	-13.1 [-26.7 to 0.5]	0.064
Plain language	Not shown	66/99 (66.7%)	0.65 [0.34 to 1.23]	-8.9 [-21.9 to 4.1]	0.185
Plain language	Shown	59/90 (65.6%)	0.61 [0.32 to 1.19]	-10.0 [-23.4 to 3.3]	0.147
GRADE language	Not shown	53/79 (67.1%)	0.66 [0.33 to 1.30]	-8.5 [-22.3 to 5.3]	0.229
GRADE language	Shown	63/101 (62.4%)	0.54 [0.28 to 1.01]	-13.2 [-26.3 to -0.1]	0.054

[1]Number (n) of participants randomized to the intervention (N) answering correctly or as anticipated. [2]Odds ratios include the main and interaction effects. [3]Risk differences account for uncertainty on the baseline odds.

Risk differences for feeling that the decision about wearing glasses if there were a surge of COVID cases was hard to make

Additional table S23. Norwegian trial

The answer about wearing glasses if there were a surge of COVID was hard for me to give.
Strongly agree
Agree
Disagree
Strongly disagree

Overall uncertainty	Margin of Error	n/N (%)[1]	Odds ratio [95% CI][2]	Risk difference [95% CI][3]	p-value
No explicit language	Not shown	63/80 (78.8%)	1	0	NA
No explicit language	Shown	62/71 (87.3%)	1.86 [0.77 to 4.49]	8.57 [-3.27 to 20.42]	0.168
Plain language	Not shown	70/76 (92.1%)	3.15 [1.17 to 8.48]	13.36 [2.53 to 24.18]	0.023
Plain language	Shown	71/79 (89.9%)	2.39 [0.97 to 5.93]	11.12 [-0.04 to 22.29]	0.059
GRADE language	Not shown	59/71 (83.1%)	1.33 [0.58 to 3.01]	4.35 [-8.16 to 16.85]	0.499
GRADE language	Shown	53/75 (70.7%)	0.65 [0.31 to 1.35]	-8.08 [-21.74 to 5.57]	0.248

[1]Number (n) of participants randomized to the intervention (N) answering correctly or as anticipated. [2]Odds ratios include the main and interaction effects. [3]Risk differences account for uncertainty on the baseline odds.

Additional table S24. US trial

The answer about wearing glasses if there were a surge of COVID was hard for me to give.
Strongly agree
Agree
Disagree
Strongly disagree

Overall uncertainty	Margin of Error	n/N (%)[1]	Odds ratio [95% CI][2]	Risk difference [95% CI][3]	p-value
No explicit language	Not shown	79/86 (91.9%)	1	0	NA
No explicit language	Shown	81/88 (92.0%)	1.03 [0.34 to 3.06]	0.2 [-7.9 to 8.3]	0.964
Plain language	Not shown	86/99 (86.9%)	0.59 [0.22 to 1.54]	-5.0 [-13.8 to 3.8]	0.280
Plain language	Shown	83/90 (92.2%)	1.05 [0.35 to 3.13]	0.4 [-7.6 to 8.4]	0.929
GRADE language	Not shown	70/79 (88.6%)	0.69 [0.24 to 1.95]	-3.3 [-12.3 to 5.8]	0.482
GRADE language	Shown	88/101 (87.1%)	0.60 [0.23 to 1.58]	-4.7 [-13.5 to 4.0]	0.301

[1]Number (n) of participants randomized to the intervention (N) answering correctly or as anticipated. [2]Odds ratios include the main and interaction effects. [3]Risk differences account for uncertainty on the baseline odds.

Risk differences for feeling that the decision about wearing glasses was informed

Additional table S25. Norwegian trial

The information in the summary helped me make an informed decision about wearing glasses if there were a surge of COVID
Strongly agree
Agree
Disagree
Strongly disagree

Overall uncertainty	Margin of Error	n/N (%)[1]	Odds ratio [95% CI][2]	Risk difference [95% CI][3]	p-value
No explicit language	Not shown	48/80 (60.0%)	1	0	NA
No explicit language	Shown	48/71 (67.6%)	1.39 [0.71 to 2.72]	7.61 [-7.68 to 22.89]	0.333
Plain language	Not shown	40/76 (52.6%)	0.74 [0.39 to 1.40]	-7.37 [-22.90 to 8.16]	0.354
Plain language	Shown	47/79 (59.5%)	0.98 [0.52 to 1.85]	-0.51 [-15.75 to 14.74]	0.948
GRADE language	Not shown	41/71 (57.7%)	0.91 [0.48 to 1.74]	-2.25 [-17.98 to 13.47]	0.779
GRADE language	Shown	40/75 (53.3%)	0.76 [0.40 to 1.44]	-6.67 [-22.25 to 8.91]	0.403

[1]Number (n) of participants randomized to the intervention (N) answering correctly or as anticipated. [2]Odds ratios include the main and interaction effects. [3]Risk differences account for uncertainty on the baseline odds.

Additional table S26. US trial

The information in the summary helped me make an informed decision about wearing glasses if there were a surge of COVID
Strongly agree
Agree
Disagree
Strongly disagree

Overall uncertainty	Margin of Error	n/N (%)[1]	Odds ratio [95% CI][2]	Risk difference [95% CI][3]	p-value
No explicit language	Not shown	61/86 (70.9%)	1	0	NA
No explicit language	Shown	73/88 (83.0%)	1.99 [0.97 to 4.12]	12.02 [-0.38 to 24.43]	0.062
Plain language	Not shown	73/99 (73.7%)	1.15 [0.60 to 2.19]	2.81 [-10.13 to 15.74]	0.670
Plain language	Shown	64/90 (71.1%)	1.01 [0.53 to 1.94]	0.18 [-13.23 to 13.59]	0.979
GRADE language	Not shown	59/79 (74.7%)	1.21 [0.61 to 2.41]	3.75 [-9.81 to 17.32]	0.589
GRADE language	Shown	76/101 (75.2%)	1.25 [0.65 to 2.38]	4.32 [-8.45 to 17.08]	0.507

[1]Number (n) of participants randomized to the intervention (N) answering correctly or as anticipated. [2]Odds ratios include the main and interaction effects. [3]Risk differences account for uncertainty on the baseline odds.
